# CDK5RAP3 acts as a tumour suppressor in gastric cancer through the infiltration and polarization of tumour-associated macrophages

**DOI:** 10.1038/s41417-022-00515-9

**Published:** 2022-08-23

**Authors:** Jia-Bin Wang, You-Xin Gao, Yin-Hua Ye, Tong-Xing Lin, Ping Li, Jian-Xian Lin, Qi-Yue Chen, Long-Long Cao, Mi Lin, Ru-Hong Tu, Ju-Li Lin, Ze-Ning Huang, Hua-Long Zheng, Jian-Wei Xie, Chao-Hui Zheng, Chang-Ming Huang

**Affiliations:** 1 Department of Gastric Surgery, Fujian Medical University Union Hospital, Fuzhou, China; 2 Key Laboratory of Ministry of Education of Gastrointestinal Cancer, Fujian Medical University, Fuzhou, China; 3 Fujian Key Laboratory of Tumour Microbiology, Fujian Medical University, Fuzhou, China

**Keywords:** Gastric cancer, Tumour immunology

## Abstract

We have demonstrated that CDK5RAP3 exerts a tumour suppressor effect in gastric cancer, but its role in regulating tumour-associated macrophages (TAMs) has not yet been reported. Here, we show that CDK5RAP3 is related to the infiltration and polarization of macrophages. It inhibits the polarization of TAMs to M2 macrophages and promotes the polarization of the M1 phenotype. CDK5RAP3 reduces the recruitment of circulating monocytes to infiltrate tumour tissue by inhibiting the CCL2/CCR2 axis in gastric cancer. Blocking CCR2 reduces the growth of xenograft tumours and the infiltration of monocytes. CDK5RAP3 inhibits the nuclear transcription of NF-κB, thereby reducing the secretion of the cytokines IL4 and IL10 and blocking the polarization of M2 macrophages. In addition, the absence of CDK5RAP3 in gastric cancer cells allows macrophages to secrete more MMP2 to promote the epithelial-mesenchymal transition (EMT) process of gastric cancer cells, thereby enhancing the invasion and migration ability. Our results imply that CDK5RAP3 may be involved in the regulation of immune activity in the tumour microenvironment and is expected to become a potential immunotherapy target for gastric cancer.

## Introduction

Gastric cancer is one of the most common malignant tumours worldwide and the fourth leading cause of cancer-related deaths [1]. Although many international scholars have conducted a large number of clinical and experimental studies on gastric cancer, the prognosis of patients with advanced gastric cancer is still not optimistic. Studies have reported that the 5-year survival rate of patients with early gastric cancer is more than 90%, while for patients with advanced gastric cancer, despite radical surgical resection, their 5-year survival rate is around 45% [2, 3]. The tumour microenvironment (TME) contributes to the growth and metastasis of tumours, which makes traditional diagnosis and treatment methods fail to significantly improve the overall survival of patients with gastric cancer [4, 5]. The connection between tumour cells and the complex components of the TME plays an important role in the process of tumour progression [6–8].

Among the components of the TME, macrophages are receiving increasing attention due to their critical roles [9]. Circulating monocyte precursor cells are recruited into tumours by chemokines such as CC motif ligand 2 and differentiate into mature macrophages [10]. Macrophages in the TME are tumour-associated macrophages (TAMs), which can be polarized into two subtypes, the classical subtype of activated macrophages (M1) and the alternative subtype of activated macrophages (M2), and they usually have the opposite effect on tumour progression [11]. The polarization of TAMs to the M1 or M2 phenotype is regulated by various microenvironmental signals from tumour cells, including cytokines secreted by tumour cells [12, 13]. Macrophages are polarized into the M2 type in the presence of IL4 or IL10, which promote tumour progression through complex autocrine and paracrine pathways [14–16]. A variety of soluble cytokines secreted by TAMs can promote the epithelial-mesenchymal transition (EMT) process of tumour cells [17]. In particular, the matrix metalloproteinase (MMP) family has attracted increasing focus for its role in promoting the invasion and migration of tumour cells [18]. However, the specific mechanism by which M2 macrophages are generated in gastric cancer is still unclear and may be crucial for identifying effective immunotherapeutic targets for gastric cancer.

In recent years, we have performed multiple studies on cyclin-dependent kinase 5 (CDK5) regulatory subunit-associated protein 3 (CDK5RAP3, also known as C53/LZAP) in gastric cancer. Our preliminary studies found that CDK5RAP3 inhibited the phosphorylation of AKT and then inhibited the phosphorylation of GSK-3β, thereby degrading β-catenin by phosphorylation [19, 20]. In a recent study on the molecular mechanism of CDK5RAP3, we demonstrated that CDK5RAP3 is inhibited by ERK signalling and negatively regulates the self-renewal of gastric cancer stem-like cells (CSCs) and EMT [21]. Recent studies have shown that the TME provides shelter for CSCs through the presence of immune cells, increasing the proliferation, tumourigenicity and drug resistance of CSCs [22, 23]. However, the role of CDK5RAP3 in regulating the TME has not been reported, and the immunomodulatory role of CDK5RAP3 urgently needs to be explored.

In the present study, we proved that the expression of CDK5RAP3 in gastric cancer cells not only inhibited the infiltration of macrophages but also inhibited the polarization of TAMs to the M2 phenotype. In addition, the absence of CDK5RAP3 in gastric cancer cells induces macrophages to secrete high levels of MMP2, thereby promoting the migration and invasion of gastric cancer cells. These findings suggest that CDK5RAP3 might become an effective therapeutic target for antitumour immunotherapy.

## Results

### CDK5RAP3 is associated with better prognosis and low expression of M2-like macrophages in gastric cancer

We obtained the gastric cancer mRNA expression matrix from the TCGA database, and CIBERSORT was used to calculate the relative proportion of CDK5RAP3-related immune cell types. CDK5RAP3 levels impacted the infiltration of memory B cells (up in CDK5RAP3 high), CD4^+^ memory-activated T cells (up in CDK5RAP3 high), follicular helper T cells (up in CDK5RAP3 high), gamma delta T cells (up in CDK5RAP3 high), M2 macrophages (down in CDK5RAP3 high) and neutrophils (down in CDK5RAP3 high) in tumour tissues (Figure S1). Next, the TIMER database was used to visualize the correlation between CDK5RAP3 and the levels of immune infiltration in gastric cancer. Among the six main immune infiltrating cells, CDK5RAP3 only has a significant negative correlation with the level of macrophage infiltration in gastric cancer (Fig. S2A). Higher macrophage infiltration is related to worse clinical prognosis, and high expression of CDK5RAP3 is related to better clinical prognosis (Fig. S2B). CDK5RAP3 was significantly positively correlated with M1-like macrophages and negatively correlated with M2-like macrophages in several analyses (Fig. S3).

**Fig. 1:**
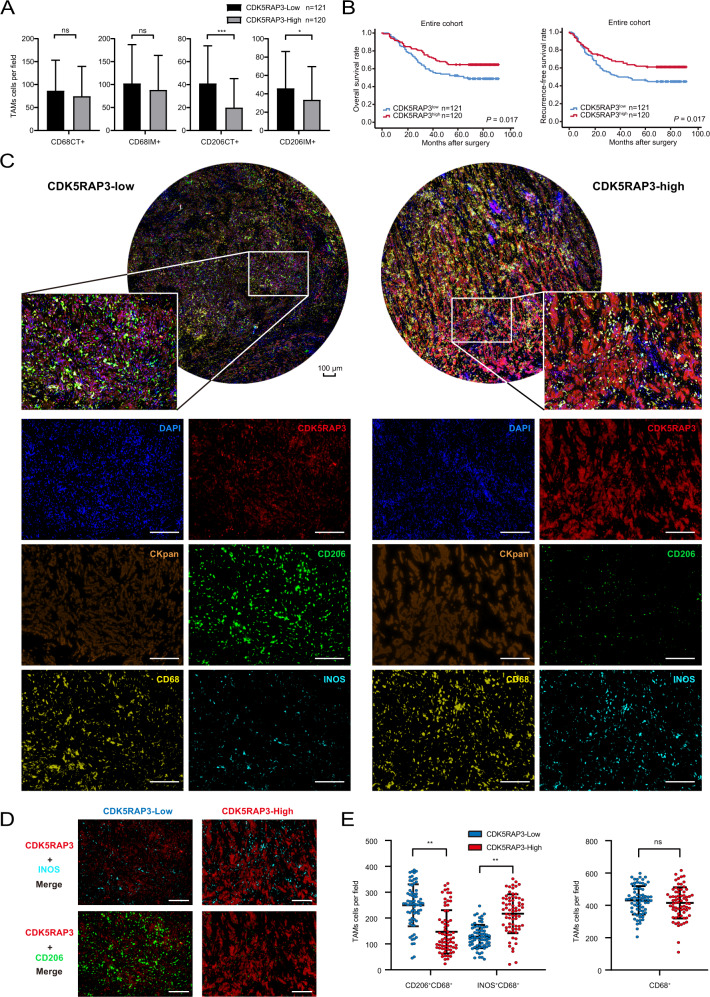
In human gastric cancer, low expression of CDK5RAP3 is related to poor clinical prognosis and higher M2 content. **A** The number of CD68+ and CD206+ cells in centre of the tumour (CT) and invasive margin (IM) sections of gastric cancer tissues in gastric cancer patients (*n* = 241) with different CDK5RAP3 expression levels. Data are presented as the mean ± SD and were analysed using Student’s *t*-test. **B** Kaplan–Meier analysis of RFS and OS in gastric cancer patients based on the expression of CDK5RAP3 in gastric cancer (*n* = 241). **C** Multiplex fluorescent immunohistochemistry of gastric cancer tissues. CKpan, DAPI, CD68, INOS, CD206 and CDK5RAP3. Scale bar = 100 μm. **D** Merged fluorescent immunohistochemical images showing the expression of CD206 and INOS in gastric cancer tissues with different expression levels of CDK5RAP3. Scale bar = 100 μm. **E** In 140 random fields of view, the number of tumour-associated macrophages (TAMs) subgroups with different expression levels of CDK5RAP3 is presented in the scatter plot. Error bars indicate estimated 95% CI (mean ± SD, *n* = 140). *P < 0.05; **P < 0.01; ***P < 0.001. *P*-values for all survival analyses were calculated using the log-rank test.

Tumour tissues from 241 patients with gastric cancer were included in the study, and the clinicopathological characteristics are shown in Table 1. In this study, low expression of CDK5RAP3 was associated with poor differentiation. IHC was performed to examine the expression of CDK5RAP3, CD68 (macrophage marker) and CD206 (M2 marker) in the centre of the tumour (CT) and invasive margin (IM) (Fig. S4A, B), and the clinicopathological characteristics are shown in Table S1. In gastric cancer tissues with high CDK5RAP3 expression, the number of CD206 CT and CD206 IM cells decreased (Fig. 1A). The high expression of CDK5RAP3 was significantly related to the low expression of CD206 IM and CD206 CT (Table S2). CDK5RAP3 is associated with a better prognosis in patients with gastric cancer (Fig. 1B). Then, we divided gastric cancer cases into 4 groups based on the expression of CDK5RAP3 and the number of TAM-positive markers (Fig. S5A, B). Statistically significant differences were present in RFS and OS among the groups with combined CDK5RAP3 and CD206 CT, but the results were not observed in the other groups of joint stratified analyses (Fig. S5A, B). We further performed multiple immunofluorescence analysis on 28 gastric cancer specimens to reveal the distribution landscape of CDK5RAP3 and TAMs in gastric cancer. Figure 1C, D shows the expression and distribution of CDK5RAP3 and macrophage markers CD68, CD206, and INOS (M1 marker). We randomly took 5 visions for each gastric cancer specimen, a total of 140 vision to calculate the correlation between the number of TAMs and the expression of CDK5RAP3. CDK5RAP3 was negatively correlated with the number of CD206^+^CD68^+^ cells and positively correlated with the number of INOS^+^CD68^+^ cells (Fig. 1E).

**Table 1:** Relationship between CDK5RAP3 expression and baseline characteristics of patients.

Variables	Total	CDK5RAP3 expression			
		Low	High	*χ*2	*P*
Gender				0.397	0.529
Male	180	93	87		
Female	61	28	33		
Age at surgery (years)				0.359	0.549
>65	100	53	47		
≤65	141	68	73		
BMI				0.451	0.502
≤25	202	22	17		
>25	39	99	103		
Tumour size (mm)				1.198	0.274
>45	122	66	56		
≤45	119	55	64		
Location of primary tumour				3.869	0.276
Lower 1/3	113	59	54		
Middle 1/3	39	18	21		
Upper 1/3	57	24	33		
More than 1/3	32	20	12		
Chemotherapy				1.589	0.207
No	97	54	43		
Yes	144	67	77		
Degree of differentiation				8.854	**0.003**
Well/moderate	74	26	48		
Poor and not	167	95	72		
Depth of invasion				0.748	0.862
T1	31	15	16		
T2	23	11	12		
T3	99	53	46		
T4	88	42	46		
Lymph node metastasis				4.1450	0.246
N0	63	32	31		
N1	41	15	26		
N2	51	26	25		
N3	86	48	38		
TNM stage				2.297	0.317
I	39	21	18		
II	62	26	36		
III	140	74	66		

*P* < 0.05 marked in bold font shows statistical significance.

An IHC scores of 4 points were used to stratify tumours with low versus high CDK5RAP3 expression.

Based on the correlation result between CDK5RAP3 and TAMs, we hypothesized that CDK5RAP3 may affect the infiltration and polarization of TAMs in the microenvironment, thereby affecting the prognosis of gastric cancer patients.

### CDK5RAP3 in gastric cancer inhibits M2-like macrophage polarization in vivo

**Fig. 2:**
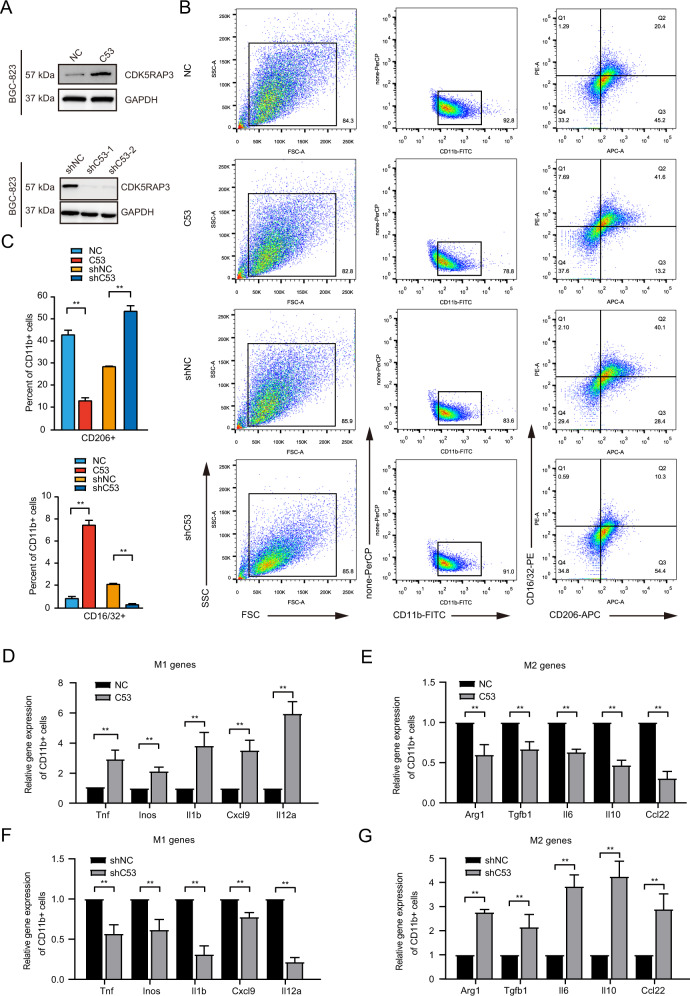
The xenograft tumour model proves that the high expression of CDK5RAP3 in gastric cancer inhibits the polarization of macrophages towards the M2 type. **A** Western blotting was used to detect the protein expression of CDK5RAP3 in the protein extract of the gastric cancer cell line BGC-823. **B** Flow cytometry was used to detect the expression of CD16/32 and CD206 on the surface of CD11b + macrophages and to determine the percentage of CD16/32+ and CD206+ cells in CD11b + macrophages. **C** The bar graphs present the difference of Q1 quadrant (representing the percentage of CD16/32-PE staining positive cells) and Q3 quadrant (representing the percentage of CD206-APC staining positive cells) in different treatment groups in FACS plots. **D**, **E** Relative gene expression of M1 markers (Tnf, Inos, Il1b, Cxcl9 and Il12a) and M2 markers (Arg1, Tgfb1, Vegfa, Il6, Il10 and Ccl22) (**F**, **G**) in the tumour tissues of mice. *P < 0.05; **P < 0.01.

BGC-823 gastric cancer cells with stable overexpression or knockdown of CDK5RAP3 were created (Fig. 2A). We then performed tumour transplantation experiments on nude mice. The results of F4/80 staining indicated an increased number of F4/80^+^ cells in xenograft tumours with low CDK5RAP3 expression. The number of F4/80^+^ cells in xenograft tumours with high CDK5RAP3 expression was significantly reduced (Fig. S6A, S6B). The number of CD206^+^ cells in xenograft tumours with high CDK5RAP3 expression was significantly less than the number of CD206^+^ cells in the control group, while CD206^+^ cells in xenograft tumours with low CDK5RAP3 expression increased significantly (Fig. S6A, S6B). Conversely, high expression of CDK5RAP3 increased CD86^+^ cell infiltration, while low expression of CDK5RAP3 decreased infiltration of CD86^+^ cells (Fig. S6A, S6B). Magnetic beads were used to separate tumour cells and TAMs from mouse tumour tissues at the time of sacrifice (Fig. S6C). The expression of CD16/32 and CD206 was used to quantify M1-like and M2-like macrophages, and the expression of both was quantified by flow cytometry. The low expression of CDK5RAP3 led to a significant increase in CD206^+^ cells and a decrease in CD16/32^+^ cells in tumour tissues, while the high expression of CDK5RAP3 led to a decrease in CD206^+^ cells and an increase in CD16/32^+^ cells (Fig. 2B). We used bar graphs to quantify the difference in the number of Q1 quadrants (which represents the percentage of CD16/32-PE staining positive cells) and Q3 quadrants (which represents the percentage of CD206-APC staining positive cells) in different groups in FACS plots (Fig. 2C). We further constructed AGS gastric cancer cells with stable overexpression or knockdown of CDK5RAP3, and verified the above results (Fig. S7). We further determined the phenotype of these macrophages and explored the gene expression of typical M1 markers (Tnf, Inos, Il1b, Cxcl9 and Il12a) and M2 markers (*Arg1, Tgfb1, Vegfa, Il6, Il10* and *Ccl22*). Compared with the control group, the expression of *Tnf, Inos, Il1b, Cxcl9 and Il12a* in macrophages in xenograft tumours with high CDK5RAP3 expression was significantly increased (Fig. 2D), while the expression of *Arg1, Tgfb1, Vegfa, Il6, Il10* and *Ccl22* was significantly reduced (Fig. 2E), indicating a predominant M1 macrophage phenotype. Compared with the control group, the expression of *Tnf, Inos, Il1b, Cxcl9* and *Il12a* in macrophages in xenograft tumours with low CDK5RAP3 expression was significantly reduced (Fig. 2F), and the expression of *Arg1, Tgfb1, Vegfa, Il6, Il10* and *Ccl22* was significantly increased (Fig. 2G), illustrating a predominant M2 macrophage phenotype.

These results indicated that CDK5RAP3 can promote the polarization of TAMs to M1 macrophages and inhibit the polarization of TAMs to M2 macrophages in vivo.

### CDK5RAP3 in gastric cancer promotes polarization of M1-like macrophages in vitro

**Fig. 3:**
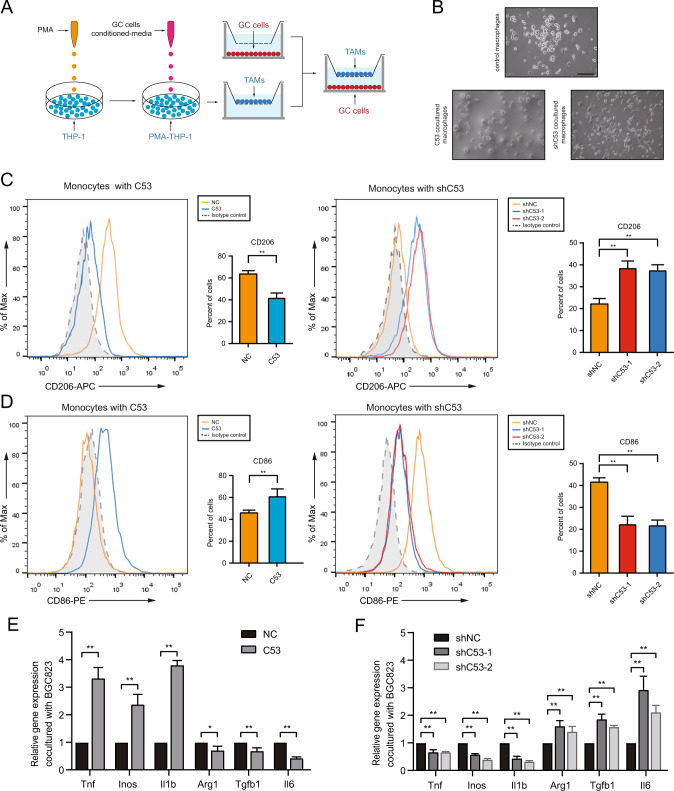
CDK5RAP3 in gastric cancer cells induces macrophages to differentiate into M1 polarized macrophages in vitro. **A** Schematic diagram showing the experimental process of coculture of gastric cancer cells and macrophages. **B** Typical bright-field images of macrophages processed by each coculture system are shown. (Magnification, ×200). Scale bar = 100 μm. **C** Flow cytometry was used to detect the expression of CD206 on the surface of differentiated macrophages. *P < 0.05; **P < 0.01, compared with Vector. **D** Flow cytometry was used to detect the expression of CD86 on the surface of differentiated macrophages. *P < 0.05; **P < 0.01, compared with Vector. **E** Under the method shown in **A**, the gene expression of TNF, INOS, IL1B, ARG1, TGFB1 and IL6 in monocytes was detected. *P < 0.05; **P < 0.01, compared with Lenti-emp. **F** Under the method shown in **A**, the gene expression of TNF, INOS, IL1B, ARG1, TGFB1 and IL6 in monocytes was detected. *P < 0.05; **P < 0.01, compared with Lenti-scr.

To further verify the result of CDK5RAP3 inhibiting M2 polarization in vitro, TAMs and BGC-823 cells were cocultured in a coculture Transwell system (Fig. 3A). The morphological changes of TAMs were observed under a microscope (Fig. 3B). Flow cytometry analysis showed that the overexpression of CDK5RAP3 in gastric cancer cells significantly reduced the number of CD206^+^ TAMs differentiated from THP-1, while the low expression of CDK5RAP3 significantly increased the proportion of CD206^+^ TAMs (Fig. 3C). The proportion of CD86^+^ TAMs was increased under coculture conditions with gastric cancer cells overexpressing CDK5RAP3. Low expression of CDK5RAP3 resulted in lower levels of the M1-like macrophage marker CD86 (Fig. 3D). We also validated the above results in AGS gastric cancer cells stably overexpressed or knocked down CDK5RAP3 (Fig. S8). Similar patterns of TAMs markers were observed by RT-PCR. Increased expression of CDK5RAP3 significantly increased the mRNA levels of typical M1 markers (*Tnf, Inos* and *Il1b*) in macrophages and decreased in the mRNA levels of M2 markers (*Arg1, Tgfb1* and *Il6*) (Fig. 3E). In contrast, decreased expression of CDK5RAP3 promoted the mRNA levels of M2 markers (*Arg1, Tgfb1* and *Il6*) while decreased the mRNA levels of M1 markers (*Tnf, Inos* and *Il1b*) (Fig. 3F).

These results further prove that the expression of CDK5RAP3 in gastric cancer inhibits the differentiation of macrophages to the M2-like phenotype and promotes the differentiation of the M1-like phenotype.

### Low expression of CDK5RAP3 in gastric cancer recruits monocytes by promoting the secretion of CCL2

**Fig. 4:**
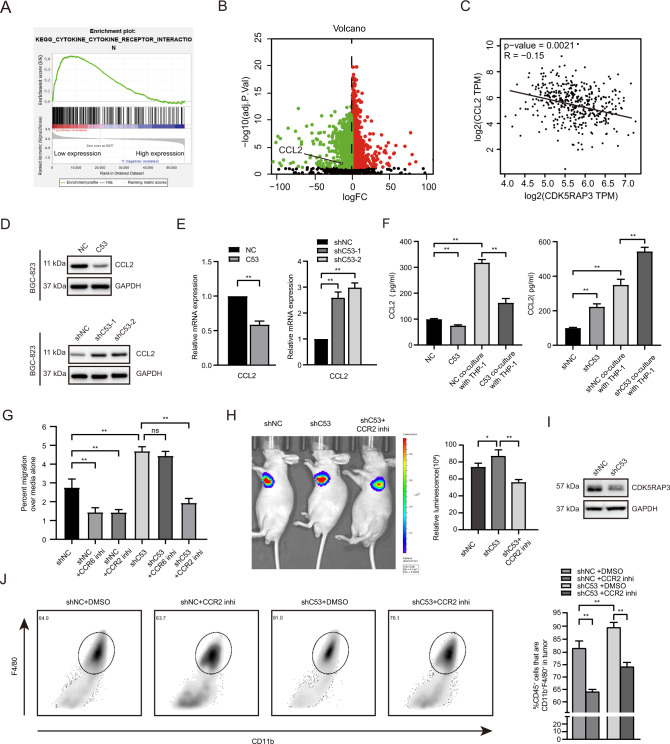
CDK5RAP3 in gastric cancer affects the infiltration of monocytes by regulating the secretion of CCL2. **A** Gene set enrichment analysis (GSEA) revealed an enrichment of ‘cytokine-cytokine receptor interaction’ signalling involved in tumours with low CDK5RAP3 expression (NES = 1.482, *p* = 0.000). **B** Volcano plot shows differentially expressed genes between CDK5RAP3high tumours relative to CDK5RAP3low tumours. **C** Spearman correlation analysis of CCL2 and CDK5RAP3 mRNA (TCGA database) levels. **D** Western blotting was used to detect the protein expression of CCL2 in the protein extract of the gastric cancer cell line BGC-823. **E** CCL2 expression in stably transfected BGC-823 cells was determined by RT-PCR. **F** CCL2 expression in the supernatant of gastric cancer cell and macrophage coculture system was determined by ELISA. **G** Small molecule antagonists of the CCL2-CCR2 interaction that specifically inhibit the chemotaxis of monocytes were determined by Transwell chemotaxis chamber in vitro. **H** Representative bioluminescence images of mice 4 weeks after tail vein injection of BGC-823 cells, and the images were quantified (*n* = 4 per group). **I** Western blotting was used to detect the expression of CDK5RAP3 in xenograft tumours. **J** Flow cytometry showed and quantified the proportion of CD11b and F4/80 cells in the total macrophages of gated single tumours (*n* = 6 per group). *P < 0.05; **P < 0.01.

To detect how CDK5RAP3 affects macrophage infiltration, GSEA was used to determine the pathways activated in CDK5RAP3 low-expressing tumours compared with CDK5RAP3 high-expressing tumours based on the TCGA database. We identified KEGG ‘cytokine-cytokine receptor interaction’ as the significantly upregulated pathway (Fig. 4A). We identified that CCL2, one of the CC motif chemokine ligands, was significantly enriched in the KEGG ‘cytokine-cytokine receptor interaction’ pathway (Table S3). CCL2 is overexpressed in gastric cancer with low CDK5RAP3 expression, and the expression of the two is significantly negatively correlated (Fig. 4B, C). Overexpression of CDK5RAP3 inhibited the secretion of CCL2, while downregulation of CDK5RAP3 promoted the secretion of CCL2 (Fig. 4D). The same pattern was validated in AGS gastric cancer cells with stable overexpression or knockdown of CDK5RAP3 (Fig. S9A). The mRNA levels of CCL2 were decreased in gastric cancer cells overexpressing CDK5RAP3 but increased in gastric cancer cells with downregulation of CDK5RAP3 (Fig. 4E). The coculture supernatants of BGC-823 cells expressing different CDK5RAP3 and monocytes were confirmed by ELISA. Overexpressed CDK5RAP3 significantly reduced the secretion of CCL2 (Fig. 4F). In contrast, the cell culture supernatant of the CDK5RAP3-low group had significantly increased levels of CCL2. In addition, coculture of gastric cancer cells with monocytes increased the secretion of CCL2 (Fig. 4F). Considering that both gastric cancer cells and monocytes secrete the chemokine CCL2, we used small molecular inhibitors to explore the chemotactic function of monocytes. We pre-treated macrophages with a small molecule inhibitor of CCR, and then plated them in the upper chamber for co-culture experiments. Monocyte migration towards the lower chamber of the control group was specifically inhibited by small molecular inhibitors of CCL2-CCR2 and CCL20-CCR6 interactions (Fig. 4G). Low expression of CDK5RAP3 significantly increased the migration rate of monocytes in the upper chamber, but only a small molecule inhibitor of the CCL2-CCR2 interaction significantly reversed this phenomenon (Fig. 4G). A xenograft tumour model was established to verify the role of CDK5RAP3 in the recruitment of monocytes. In vivo bioluminescence imaging showed that downregulation of CDK5RAP3 enhanced cell proliferation in situ, while the small molecule inhibitor of the CCL2-CCR2 interaction significantly alleviated this impairment (Fig. 4H). Western blotting was used to verify the expression of CDK5RAP3 in different xenograft tumour model groups (Fig. 4I). To precisely analyze the number of monocytes in the xenograft tumour model, we identified them by CD11b^+^F4/80^+^ gated flow cytometry. It showed that low expression of CDK5RAP3 significantly increased the proportion of CD11b^+^F4/80^+^ macrophages in the total macrophages, while the small molecule inhibitor of the CCL2-CCR2 interaction significantly reversed this phenomenon (Fig. 4J).

In general, the low expression of CDK5RAP3 in gastric cancer can promote the secretion of CCL2, which recruits monocytes to infiltrate the tumour microenvironment through CCL2/CCR2 signalling.

### CDK5RAP3 reduces the expression of IL4 and IL10 by inhibiting the phosphorylation of NF-κB, thereby inhibiting M2-like polarization

**Fig. 5:**
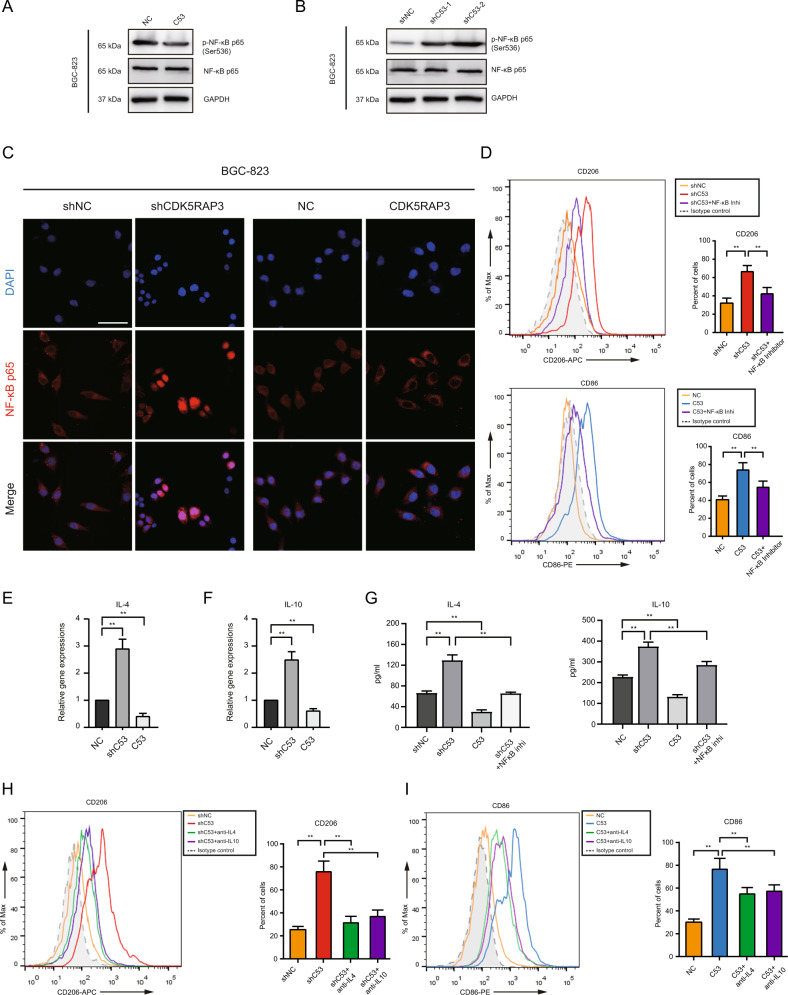
CDK5RAP3 negatively regulates the production of IL4 and IL10 by gastric cancer cells by inhibiting NF-κB nuclear transcription. **A**, **B** The expression of p-NF-κB p65 and NF-κB p65 in stably transfected BGC-823 cells was detected using Western blotting. **C** Immunofluorescence (IF) staining showed the localization of NF-κB p65 (red) and DAPI (blue) in stably transfected BGC-823 cells. Scale bar = 50 μm. **D** Flow cytometry was used to detect the expression of CD206 and CD86 on the surface of differentiated macrophages. **P < 0.01, compared with Vector. **E**, **F** Relative gene expression of IL4 and IL10 in stably transfected BGC-823 cells was determined by RT-PCR. **G** IL4 and IL10 expression in the culture supernatant of transfected BGC-823 cells was determined by ELISA. **H**, **I** Flow cytometry was used to detect the expression of CD206 and CD86 on the surface of differentiated macrophages. **P < 0.01, compared with Vector.

To explore the polarization effect of CDK5RAP3 on TAMs, we present the combination of CDK5RAP3 and NF-κB p65 (RELA) using the STRING database based on their gene expression data. We found that CDK5RAP3 was related to the transcription factor NF-κB (Fig. S9B). The phosphorylation of NF-κB p65 at Ser536 was increased in gastric cancer cells with low CDK5RAP3 expression but decreased in cells overexpressing CDK5RAP3 (Fig. 5A, B). Immunofluorescence microscopy experiments showed the localization of NF-κB p65 in cells, and low expression of CDK5RAP3 promoted the nuclear transcription of NF-κB p65 (Fig. 5C). PDTC (an NF-κB inhibitor) attenuated the upregulation of CD206^+^ macrophages induced by low-expressing CDK5RAP3 gastric cancer cells (Fig. 5D). PDTC also reduced the upregulation of CD86^+^ macrophages induced by gastric cancer cells overexpressing CDK5RAP3 (Fig. 5D). The same pattern was validated in AGS gastric cancer cells (Figs. S9C, S10A, S10B). We identified that IL4 and IL10 were significantly enriched in the KEGG ‘cytokine-cytokine receptor interaction pathway’, which is significantly activated in gastric cancer with low CDK5RAP3 expression (Table S3). We hypothesized that the low expression of CDK5RAP3 regulates the transcription factor NF-κB to initiate the transcription of cytokines IL4 and IL10 and then regulate the polarization of macrophages. We identified the potential binding sites of NF-κB in the IL4 and IL10 promoters through JASPAR and PROMO databases (Fig. S11A). Low expression of CDK5RAP3 significantly increased the mRNA levels of IL4 and IL10, while overexpression of CDK5RAP3 significantly reduced them (Fig. 5F). The levels of IL4 and IL10 were significantly reduced in gastric cancer cells overexpressing CDK5RAP3 (Fig. 5G). In contrast, the levels of IL4 and IL10 in the culture supernatant increased significantly in CDK5RAP3-depleted gastric cancer cells, while PDTC reduced the upregulation of IL4 and IL10 levels induced by gastric cancer cells with low CDK5RAP3 expression (Fig. 5G). Rescue studies have shown that neutralizing antibodies to IL4 and IL10 can attenuate the upregulation of CD206^+^ macrophages induced by gastric cancer cells with low CDK5RAP3 expression (Fig. 5H). The neutralizing antibodies against IL4 and IL10 also reduced the upregulation of CD86^+^ macrophages induced by the overexpression of CDK5RAP3 in gastric cancer cells (Fig. 5I). Similar rescue results were validated in AGS gastric cancer cells (Fig. S11B).

These results support that CDK5RAP3 reduces the secretion of IL4 and IL10 by inhibiting the phosphorylation of NF-κB p65, thereby reducing the polarization of TAMs to M2-like macrophages.

### Deletion of CDK5RAP3 in gastric cancer enhances the malignant behaviour of gastric cancer cells by promoting the secretion of MMP2 from TAMs

**Fig. 6:**
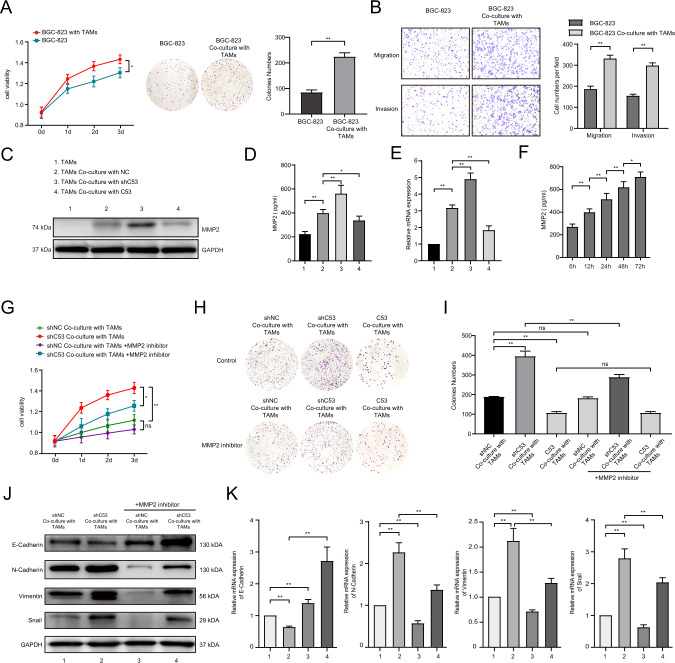
CDK5RAP3 deletion in gastric cancer mediates the tumour-promoting phenotype of macrophages and the secretion of MMP2. **A** MTT assay and colony formation assay of BGC-823 cells cocultured with macrophages or single cultures. Days = 8. **B** Transwell assays of stably transfected BGC-823 cells were performed, and the quantification of the results is presented. Days = 8. **C** Western blotting were used to determine MMP2 expression of macrophages in different coculture systems. Coculture time = 48 h. **D** ELISA were used to determine MMP2 secretion by macrophages. **E** Relative gene expression of MMP2 expression in macrophages was determined by RT-PCR. **F** ELISA were used to determine MMP2 secretion by macrophages at different cocultivation time points. **G** MTT assay of BGC-823 cells in different treatment coculture systems. **H**, **I** Colony formation assay of BGC-823 cells in different treatment coculture systems. Days = 8. **J** Western blotting was used to detect the expression of E-cadherin, N-cadherin, vimentin and Snail in gastric cancer in different treatment coculture systems. **K** Relative gene expression of E-cadherin, N-cadherin, vimentin and Snail in gastric cancer tissues was determined by RT-PCR. *P < 0.05; **P < 0.01.

The aforementioned Transwell co-culture system of TAMs and BGC-823 cells were constructed. We collected BGC-823 cells with and without co-culture with TAMs from Transwell co-culture system. Compared with BGC-823 cells not cocultured with TAMs, TAMs significantly promoted the proliferation of BGC-823 cells (Fig. 6A). The invasion and migration ability of the coculture group with TAMs was also significantly enhanced (Fig. 6B). We found that the level of the secreted protein MMP2 in TAMs cocultured with gastric cancer cells was significantly increased compared with the level of the secreted protein MMP2 in normal TAMs. The downregulation of CDK5RAP3 in gastric cancer cells further increased the expression of MMP2 in macrophages, while overexpression of CDK5RAP3 significantly reduced the expression of MMP2 (Fig. 6C). Similar expression patterns were also observed when using ELISA to detect the culture supernatant and RT-PCR to detect the mRNA expression of MMP2 in macrophages (Fig. 6D, E). Under the culture conditions of BGC-823 cells with downregulation of CDK5RAP3, the level of MMP2 secreted by macrophages increased with prolonged cocultivation time (Fig. 6F). Under the conditions of cocultivation with TAMs, the low expression of CDK5RAP3 significantly promoted the proliferation of gastric cancer cells (Fig. 6G). In addition, MMP2 inhibitor attenuated the increased proliferation of gastric cancer cells caused by low expression of CDK5RAP3, while the use of MMP2 inhibitor on gastric cancer cells in the normal control group did not show this change (Fig. 6G). We applied the supernatant of pre-cultured TAMs to the gastric cancer cell plate cloning experiment. The use of an MMP2 inhibitor attenuated colony formation caused by gastric cancer cells with low expression of CDK5RAP3 (Fig. 6H, I). MMP2 inhibitor also attenuated the increased invasion and migration ability of gastric cancer cells caused by low expression of CDK5RAP3 (Fig. S12). In order to explore the effect of MMP2 inhibitor on gastric cancer cells with downregulation of CDK5RAP3, we set up a group of gastric cancer cells with downregulation of CDK5RAP3 without macrophage co-culture. The addition of MMP2 inhibitor had no significant effect on the proliferation of gastric cancer cells with downregulation of CDK5RAP3 (Fig. S13). Under the condition of coculture of macrophages, the low expression of CDK5RAP3 significantly reduced the expression of the epithelial marker E-cadherin and increased the expression of N-cadherin, vimentin, and Snail (Fig. 6J). After adding the MMP2 inhibitor to the coculture system, the EMT process of the low-expressing CDK5RAP3 gastric cancer group was restored (Fig. 6J). Under the condition of coculture of macrophages, the downregulation of CDK5RAP3 resulted in a significant upregulation of the expression levels of N-cadherin, vimentin and Snail mRNA in gastric cancer, while the expression level of E-cadherin was significantly reduced (Fig. 6K). The addition of an MMP2 inhibitor to the coculture system significantly inhibited the EMT process of gastric cancer induced by low expression of CDK5RAP3 (Fig. 6K). We further verified the above results in AGS gastric cancer cells (Fig. S14).

Consequently, these results indicated that the lack of CDK5RAP3 in gastric cancer enhances the EMT process of gastric cancer cells by promoting the secretion of MMP2 by TAMs in vitro, thereby enhancing the proliferation, invasion, and migration ability of gastric cancer cells.

### Macrophage-derived MMP2 plays an important role in the invasion and metastasis of gastric cancer cells mediated by low-expressing CDK5RAP3 gastric cancer cells

**Fig. 7:**
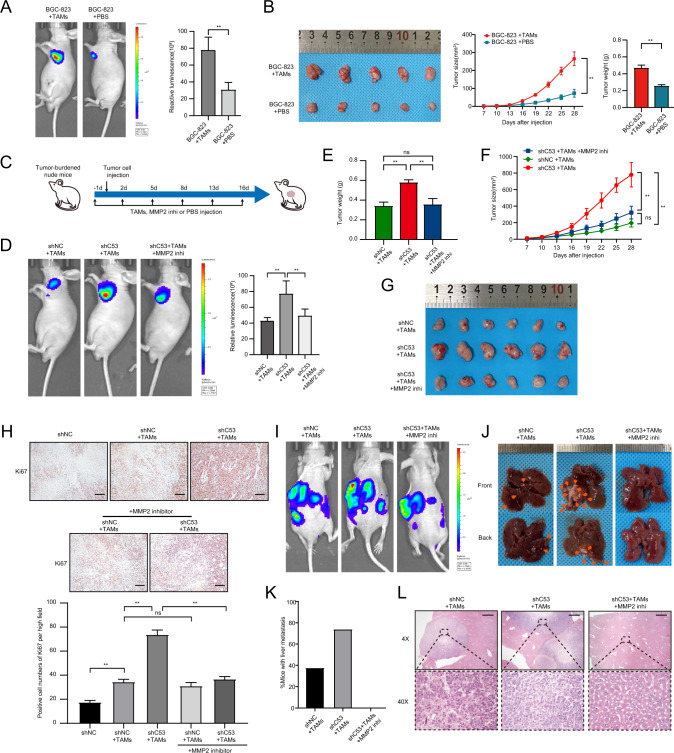
The absence of CDK5RAP3 in gastric cancer cells mediates the cancer-promoting phenotype of macrophages in a xenograft tumour model. **A** Representative bioluminescence images of mice 4 weeks after subcutaneous injection of BGC-823 cells and/or macrophages, and the images were quantified. Representative photos and tumour growth of each group of mouse tumours are shown in (**B**). The size of the xenograft was measured every 3 days until the mice were sacrificed. The average tumour weights of the three different groups were compared. (*n* = 5 per group). **C** MMP2 inhibitors were applied to the experimental design of BALB/c nude mice in xenograft tumour models. **D** Representative bioluminescence images of mice 4 weeks after subcutaneous injection of gastric cancer cells and TAMs, and the images were quantified. **E** The average tumour weights of the three different groups were compared. **F** The size of the xenograft was measured every 3 days until the mice were sacrificed. **G** Representative pictures of tumours in each group of mice are shown. (*n* = 6 per group). **H** Ki67 pathological images of tumours collected from Balb/c nude mice injected with BGC-823 cells and/or TAMs. IHC staining of Ki67 in tumour tissues in mice xenograft model and positive cell numbers per high field were counted. Scale bar = 50 μm. **I** Representative bioluminescence images of mice 4 weeks after spleen injection of BGC-823 cells and TAMs, and the images were quantified. **J** Representative images of liver metastasis. **K** The percentage of tumour metastasis in the Lenti-scr + TAMs groups, Lenti-shC53 + TAMs/PBS groups and Lenti-shC53 + TAMs/MMP2 inhi groups (*n* = 8 per group). **L** Representative haematoxylin-eosin-stained sections of liver metastases in mice 28 days after implantation in different groups. Magnification: ×4 and ×40. Scale bar = 200 µm. *P < 0.05; **P < 0.01.

Then, we injected BGC-823 + TAMs, BGC-823 + PBS and TAMs + PBS subcutaneously into nude mice to construct xenograft models. In vivo bioluminescence imaging showed that coinjection with TAMs significantly increased the in situ proliferation of BGC-823 cells compared with BGC-823 cell injection alone (Fig. 7A). There was no tumour formation after injection of TAMs + PBS (data not shown). This suggests that the tumour burden in the xenograft tumour model is at least not caused by TAMs alone, but may be influenced by some effect of TAMs on gastric cancer cells. In addition, the growth rate of these xenograft tumours in the coinjection group with TAMs was much faster than the growth rate of xenograft tumours in the coinjection group of BGC-823 cells alone, and the tumour size and weight also increased significantly (Fig. 7B). Similar results were validated in AGS gastric cancer cells (Fig. S15A). We established a xenograft tumour model by injecting TAMs, MMP2 inhibitor, and PBS for 2 weeks (Fig. 7C). In vivo bioluminescence imaging showed that low expression of CDK5RAP3 significantly increased the in-situ proliferation of gastric cancer cells compared with the control group, while the MMP2 inhibitor reversed the increase in proliferation induced by low CDK5RAP3 expression (Fig. 7D). The results showed that low expression of CDK5RAP3 significantly promoted tumour proliferation in vivo, while the MMP2 inhibitor reversed this phenomenon (Fig. 7E–G). We further validated in a xenograft tumour model of AGS gastric cancer cells (Fig. S15B). After the mice were sacrificed, western blotting was used to verify the expression of CDK5RAP3 in the xenograft tumours with BGC-823 and AGS gastric cancer lines with different treatments (Fig. S15C). Xenograft tumours were taken for Ki67 staining and counting. The results of Ki67 staining showed that compared with the control group, the BGC-823 + TAMs group had increased intratumoural proliferation compared with the BGC-823-alone group, and the low-expressing CDK5RAP3 more significantly promoted intratumoural proliferation (Fig. 7H). However, after using MMP2 inhibitor, the intratumoural proliferation of the control group was not significantly different than before, while the intratumoural proliferation of the low-expression CDK5RAP3 group was significantly lower (Fig. 7H). Next, we established a liver metastasis model and a lung metastasis model in nude mice. The three groups were all injected with BGC-823 cells and TAMs, and then the presence and rate of liver metastases were examined. The analysis results showed that three out of eight mice in the control group had liver metastases, and six out of eight mice in the low-expressing CDK5RAP3 group had liver metastases. However, on the basis of low expression of CDK5RAP3, there was no liver metastasis in 8 mice injected with MMP2 inhibitor (Fig. 7I–L). The lungs of the nude mouse lung metastasis model were taken for HE staining and lung nude mouse counts. Coinjection with macrophages significantly increased the number of lung metastases in vivo compared with injection of gastric cancer cells alone. Low expression of CDK5RAP3 further promoted the number of lung metastases in vivo, while the MMP2 inhibitor reversed lung metastasis promoted by low expression of CDK5RAP3 (Fig. S16).

Our research illustrates the crosstalk between the expression of CDK5RAP3 in gastric cancer and TAMs in TME. The low expression of CDK5RAP3 in gastric cancer cells promotes the secretion of MMP2 by macrophages, thereby enhancing the proliferation, invasion and migration ability of gastric cancer cells. We summarized our findings in a schematic diagram (Fig. S17).

## Discussion

Our discovery expands the knowledge system about the role of CDK5RAP3 in gastric cancer and is different from previous studies that were limited to gastric cancer cells themselves [19–21, 24], but aimed at its relationship with the TME of gastric cancer. Although research on TAMs has made significant progress in recent years, scholars still have controversies about the impact of TAMs on clinical outcomes [25–27]. The result of these controversies may be because most researchers ignore the distribution and different phenotypic characteristics of TAMs. Therefore, we confirmed both in CT and IM that CDK5RAP3 has a significant negative correlation with M2-like macrophages. After combining the hierarchical analysis of the expression levels of CDK5RAP3 and macrophages in different locations, only the combination of centrally infiltrated M2-like macrophages and CDK5RAP3 showed significant differences in survival. This result may be consistent with some previous studies showing that TAMs can be recruited and aggregated in tumour tissues and then interact with tumour cells [28]. These results prompted us to further explore the role of CDK5RAP3 expression in macrophage infiltration and polarization.

Our study demonstrated that low expression of CDK5RAP3 promotes CCL2/CCR2 signalling to regulate the recruitment of monocytes and macrophages to tumour tissues. Signalling between CCR2 and its ligand CCL2 is related to the disease progression of various cancers, such as breast cancer and melanoma [29, 30]. In view of the multiple roles of CCL2/CCR2 signalling in cancer, a number of anticancer treatment studies have been carried out on this signalling axis. However, the efficacy of several clinical trials using monoclonal antibodies against CCL2 has not been significant [31–33]. For example, treatment with the anti-CCL2 monoclonal antibody Carlumab in patients with prostate cancer does not have a long-lasting effect [34]. We used xenograft tumour models to evaluate the anticancer properties of CDK5RAP3 in vivo and the potential to regulate the tumour microenvironment. Therefore, the expression of CCL2 induced by low expression of CDK5RAP3 may play an important role in the recruitment of macrophages in gastric tumourigenesis. Studies have shown that TAM-infiltrated tumour tissues can be tumour-promoting or antitumour in response to different environmental cues [35, 36], implying that macrophages change their physiological functions according to environmental changes and produce different populations of cells with different functions. The loss of CDK5RAP3 expression in gastric cancer cells induces the recruitment of TAMs into tumour tissues and might cause them to evolve into a cancer-promoting phenotype.

The polarization of macrophages is a complex process that is accomplished by the secretion of multiple cytokines and pathways by macrophages and cancer cells. Previous research reports indicated that CDK5RAP3 inhibits NF-κB by reducing the phosphorylation of RELA [37]. Our current findings help to further understand the molecular mechanism by which NF-кB regulates specific immune cell-related cytokines in gastric cancer. Studies have shown that the polarization of TAMs to M2-like macrophages is activated by the cytokines IL4 and IL10 [38, 39]. Consistently, we demonstrated that cytokines mediated by low CDK5RAP3 expression in gastric cancer promoted the polarization of M2-like macrophages. It is worth mentioning that we also identified other cytokines that are significantly related to CDK5RAP3. Some cytokines that play an important role in anti-tumour immunity are significantly enriched in low CDK5RAP3 expression. Among them, CSF1/CSF1R, VEGFs, and IL13 are essential for the differentiation and survival of macrophages [40], suggesting that the retention of CDK5RAP3 can weaken the survival and cancer-promoting function of M2-like macrophages, and even make newly infiltrated macrophages revert to the anti-tumour phenotype. Some cytokines that can stimulate immature myeloid cells to differentiate into myeloid-derived suppressor cells, including TGFβ, IL1β and IL6, have also been identified [41]. In short, it is necessary to develop immunotherapy targets for CDK5RAP3 targeting macrophages to provide a broader vision for cancer immunotherapy with more personalized treatment methods.

There is current controversy over whether TAMs have cancer-promoting or cancer-suppressing functions in gastric cancer [11, 27, 38]. Considering that the functional changes of TAMs are driven by tumour environmental factors, TAMs may not be purely polarized to M1-like or M2-like macrophages when gastric cancer releases different mediators but exhibit cancer-promoting and anticancer properties. Our results demonstrated that mixed phenotypic TAMs, especially TAMs mediated by low expression of CDK5RAP3 in gastric cancer, are an important functional mediator of maintaining the progression of gastric cancer. Increasing clinical evidence has shown the role of infiltrating TAMs in promoting tumour progression and metastasis by producing soluble factors [25, 26, 42]. Our previous studies have demonstrated that CDK5RAP3 negatively regulates the EMT process of gastric cancer cells [21]. Accordingly, we speculate that part of the potential mechanism by which CDK5RAP3 mediates gastric cancer cell migration, invasion and EMT is at least accomplished by regulating the expression of MMP2 in macrophages. In addition, M2-like macrophages can secrete inhibitory cytokines to limit the immune response, promote tumour progression, and prevent T cells from exerting effective antitumour effects [43]. Here, we found that macrophages mediated by low CDK5RAP3 expression have higher expression of IL6, IL10 and TGFβ1, which have been reported to drive self-renewal of CSCs by activating the STAT3/NF-κB signalling pathway [44]. In view of the fact that the TME can provide a protective impact for CSCs through the existence and function of immune cells [45], in-depth exploration of the crosstalk between CDK5RAP3-mediated immune cells and CSCs is expected to provide new candidate targets and intervention methods for CSCs. In short, the method of restoring the expression of CDK5RAP3 in gastric cancer to regulate MMPs from macrophages is expected to provide a new perspective for immunotherapy to reduce the progression and metastasis of gastric cancer.

The present study obviously has limitations that must be acknowledged. The specific mechanism by which CDK5RAP3 inhibits CCL2 secretion needs to be further explored. Second, the classification of TAMs into M1-like and M2-like macrophages is an artificial result in vitro, and this classification is inevitably somewhat limited. The evidence for the immunosuppression of gastric cancer with a specific phenotype associated with CDK5RAP3 still needs to be further explored. In summary, CDK5RAP3 is involved in the regulation of the immune activity state of the TME, and it is expected to become a promising biomarker for the treatment and prognosis of gastric cancer.

## Materials and methods

### Patients and gastric tissue samples

This research included a total of 250 gastric cancer tissues collected from January 2010 to October 2015; nine samples were excluded due to missing data. The remaining 241 patients were employed for the internal cohort. Gastric tissue specimens were fixed with formalin and embedded in paraffin. The inclusion criteria were as follows: (a) histological identification of gastric cancer; (b) availability of follow-up data and clinicopathological characteristics; and (c) TNM staging of gastric cancer tumours according to the 2010 International Union Against Cancer (UICC) guidelines. The exclusion criteria were as follows: (1) patients with no formalin-fixed paraffin-embedded (FFPE) tumour sample, including the centre of the tumour (CT) and the invasive margin (IM), from initial diagnosis; and (2) patients who received chemotherapy or radiotherapy before surgery. All participating patients with advanced GC routinely received fluorine-based chemotherapy. Comprehensive information of the internal cohort is listed (Table 1). All procedures performed in studies involving human participants were in accordance with the Helsinki declaration. And all patients whose tissue samples were used in this research provided written informed consent. This study was approved by the Ethics Committee of Fujian Medical University Union Hospital (Ethics approval number of scientific research project: 2021KJT031).

### Immunohistochemistry and evaluation

The serial sections of the FFPE sample were 4 μm and mounted on a glass slide for IHC analysis. The sections were deparaffinized with xylene and rehydrated with alcohol. We blocked endogenous peroxidase by immersing the slices in a 3% H_2_O_2_ aqueous solution for 10 min and microwaved the slices in 0.01 mol/L sodium citrate buffer (pH 6.0) for 10 min for antigen retrieval. The slides were then washed in phosphate-buffered saline (PBS) and incubated with 10% normal goat serum (Zhongshan Biotechnology Co., Ltd., China) to eliminate nonspecific reactions. Subsequently, the sections were incubated overnight with the primary antibody at 4 °C. The negative control was processed with the same methods, but the primary antibody was omitted. After rinsing three times with PBS, the secondary antibody was diluted and incubated on slides for 30 min at room temperature, and the staining was developed with diaminobenzidine (DAB) solution. Finally, the slides were counterstained with haematoxylin, dehydrated, and fixed with a cover glass and neutral resin.

We performed CDK5RAP3 (ab157203, Abcam, 1:100) immunohistochemical staining on tumour tissue of gastric cancer patients. The staining intensity and average percentage of positive cells in 5 randomly selected regions were evaluated to represent the protein expression level. The scoring criteria (Fig. S4A) were as follows: staining intensity was divided into 0 (negative staining), 1 (weak staining, light yellow), 2 (medium staining, yellow-brown), or 3 (strong staining, brown). The proportion of positively stained tumour cells was categorized according to the following thresholds: 0 (≤5% positive cells), 1 (6–25% positive cells), 2 (26–50% positive cells), and 3 (≥51% positive cells). The final expression was calculated by multiplying the staining intensity score by the proportional staining score (total 0–9). Patients with final scores of <4 points were classified as the low expression group, and patients with scores ≥4 were classified as the high expression group.

CD68 (ab213363, Abcam, 1:300) and CD206 (ab64693, Abcam, 1:500) were subjected to immunohistochemical staining. To evaluate the infiltration of immune cells, five representative and independent fields at ×200 magnification were captured at the centre of the tumour (CT) and invasive margin (IM), respectively, on each tissue. Next, we used the ‘measurement’ plug-in in Image-Pro Plus software (6.0, Media Cybernetics Inc.) to assist in marker counting to obtain the number of positive cells in the field. The average number of positive cells in five fields was divided by the area of the field (0.27 mm^2^) to obtain the infiltration density of immune cells in CT and IM. All the percentages/numbers of positive cells were expressed as the average of five randomly selected microscopic fields.

The IHC results were evaluated by two independent gastroenterology pathologists who were blinded to the clinical prognosis of the patients. Approximately 90% of the scoring results were the same. When the scores of the two independent pathologists diverged, another pathologist checked the results again and selected one of the scores proposed by the first two doctors, or the three pathologists discussed the decision together.

### Multiplexed immunofluorescence staining

We performed multiplexed immunofluorescence staining to identify the expression of CDK5RAP3 (ab157203, Abcam, 1:50), CD68 (M0876, DAKO, 1:500), CD206 (CL488-60143, Proteintech, 1:300) and INOS (ER1706-89, HuaAn, 1:100) in 28 gastric cancer tissues. Tumour cytokeratin was stained with CKpan. All nuclei were stained with DAPI. Briefly, formalin-fixed paraffin-embedded tissue sections were cut into 4-mm thick sections, thawed at 70 °C for 45 min, deparaffinized and fixed with formaldehyde: methanol (1:10). Then, in a pH 8.0 EDTA buffer and heat-induced antigen recovery was performed at 100% power in an 800 W standard microwave until the boiling point, and then 30% power was used for 15 min. The tissue sections were then cooled and washed in 0.02% Tris-buffered saline-Tween 20 (TBST) with gentle stirring. Then, the sections were blocked with blocking buffer (Dako, X0909) for 10 min at room temperature and then incubated with the primary antibody at 4 °C overnight. Then, the horseradish peroxidase (HRP)-conjugated secondary antibody (PerkinElmer) was incubated at room temperature for 1 h, and then the tyramide-based HRP was activated at 37 °C for 20 min. The stained signal was further amplified using Opal 540 Acetamide Signal Amplification (TSA) reagent (PerkinElmer) and incubated with TSA dilution at room temperature. Using TSA, HRP-conjugated secondary antibodies mediate the covalent binding between the Pax-5 protein and different fluorophores. After this covalent reaction, additional antigen recovery (pH 6.0 citrate buffer) was performed for 20 min to remove the bound antibody. Note: Repeat all steps in sequence for each primary antibody. Then, after counterstaining with 4′,6-diamidino-2-phenylindole (Life Technologies) at room temperature, all sections were washed five times in 0.02% TBST for 5 min each for 2 min and stored in a 4 °C lightproof box C until imaging.

### Establishment of cell lines

The cell lines were established as previously described [19].

### Cell culture and reagents

The human gastric cancer cell lines BGC-823 and AGS were obtained from the Cell Line Bank, Chinese Academy of Sciences. All the cell lines were confirmed to be free of mycoplasma contamination by PCR and culture. The species origin was confirmed with PCR. The identity of the cell line was authenticated with short tandem repeat (STR) profiling. These cell lines were cultured in 1640 (Gibco, Grand Island, NY) supplemented with 10% foetal bovine serum (FBS) (Gibco, Grand Island, NY) and incubated at 37 °C in a humidified atmosphere containing 5% CO_2_. The human monocyte line THP-1 was purchased from Guangzhou Cellcook Biotechnology Co., Ltd. To obtain macrophages, 3 × 10^5^ THP-1 cells were seeded in 0.4 μm pore inserts treated with 200 nM PMA (Sigma-Aldrich, CN) for 24 h and polarized into macrophages. To obtain TAMs, THP-1 macrophages were cultured by the addition of conditioned media from a gastric cancer cell line (BGC-823) for 24 h. Macrophage and gastric cancer cell cocultivation was conducted using a noncontact coculture Transwell system (Corning, CN). Inserts containing TAMs or THP-1 macrophages were transferred to 6-well plates seeded with gastric cancer cells (1 × 10^5^ cells per well) in advance and cocultured. After 48 h of coculture, TAMs or gastric cancer cells were harvested for further analyses.

PDTC (ab141406, ammonium pyrrolidinedithiocarbamate, Abcam), a selective NF-κB inhibitor, was purchased from Abcam Co., Ltd., Shanghai, China. RS 102895 hydrochloride (ab120812, Abcam), a selective CCR2 receptor antagonist, was purchased from Abcam Co., Ltd., Shanghai, China. MMP-2 inhibitor (ab145190, Abcam) was purchased from Abcam Co., Ltd., Shanghai, China. PMA (P1585, Sigma-Aldrich) was dissolved in PBS containing 0.1% BSA and used at a final concentration of 100 ng/ml.

### Cell coculture system

BGC-823 cells were seeded into the lower chamber, and THP-1 cells or primary monocytes (1:1 ratio) were added into the upper chamber of a 6-well Transwell apparatus (Corning Costar). A membrane with a pore size of 0.4 μm separated the cell lines, preventing physical interaction between monocytes and BGC-823 cells while allowing signalling and crosstalk to take place. Coculture times are indicated in the text.

### Mouse xenograft models

All male BALB/c nude mice (4–5 weeks old) used in our study were purchased from Beijing Vital River Laboratory Animal Technology Co., Ltd. For tumour growth experiments, BGC-823 cells (5 × 10^5^), TAMs (5 × 10^5^), BGC-823 cells (5 × 10^5^) with TAMs (5 × 10^5^) in 200 μl were subcutaneously injected into the right axillary fossa of nude mice. Tumour volume was measured every 3 days and calculated with the following formula: *V* = (*L* × *W*^2^)/2 cm^2^ (*V*, tumour volume; *L*, length; *W*, width). The mice were sacrificed at 3–4 weeks after injection, and the tumours were weighed. For the lung or liver metastasis model, BGC-823 cells (5 × 10^5^), TAMs (5 × 10^5^), BGC-823 cells (5 × 10^5^) with TAMs (5 × 10^5^) in 200 μl were injected into the tail veins or spleen of nude mice. Forty-five days later, the mice were sacrificed, and the lungs or livers were dissected to examine the histopathological metastatic loci. All animal experiments were performed according to the Animal Protection Committee of Fujian Medical University (Fuzhou, China) and approved by the Ethics Committee of Fujian Medical University/Laboratory Animal Center (Fuzhou, China). The tumour tissues and liver or lung tissues of mice were further examined by H&E, IHC staining, or RT-PCR assay.

All animal experiments were performed according to the Animal Protection Committee of Fujian Medical University (Fuzhou, China) and approved by the Ethics Committee of Fujian Medical University/Laboratory Animal Center (Fuzhou, China).

### Flow cytometry

For flow cytometry, cells were washed twice with PBS and blocked with FcR Blocking Reagent (human/mouse) (Miltenyi Biotec GmbH). Cells were then stained with CD206-APC (mouse), CD16/32-PE. Cy7 (mouse), CD11b-FITC (mouse), F4/80-APC (mouse), CD206-APC (human), CD86-PE (human), CD11b-PE. Cy7 (human) antibodies (Invitrogen eBioscience, Thermo Fisher Scientific Co., Ltd., China). Separated mouse macrophages were permeated and fixed using a Cytofix/CytoPerm Plus™ kit (BD Biosciences) following the manufacturer’s instructions and then stained with CD206 and CD16/32 antibodies. Cells were resuspended in FACS buffer (PBS with 10% FBS) and run on a BD Flow Cytometer LSRFortessaX-20 (BD Biosciences). Data were analysed using FlowJo Software v10.0 (Ashland, OR). All tests were controlled by homologous isotype control antibodies.

### RNA isolation and RT-PCR

Total RNA of cells was extracted with TRIzol reagent (Invitrogen, USA), and cDNA was synthesized using a Reverse Transcription Reagent Kit (Takara, Dalian, China) according to the manufacturer’s instructions. Quantitative PCR (qPCR) was conducted using a SYBR® Premix DimerEraser kit (Takara) with a Stratagene MX3005P cycler according to the manufacturer’s instructions. The relative quantification of the expression of each gene was normalized to the relative quantification of the expression of β-actin mRNA. The sequences of specific primers are listed in Table S4.

### Isolation of macrophages and tumour cells from mouse tumour tissue

Single-cell suspensions were prepared from fresh tumours using a Tumour Dissociation Kit (Miltenyi Biotec GmbH). Cells were then immediately separated using a negative magnetic bead-assisted sorting assay (Mouse Cell Depletion Kit). TAMs were separated from the positive cell suspensions using CD11b + magnetic beads. All operations were performed according to the manufacturer’s protocol.

### Western blot assay

Cells were plated into 60-mm dishes and cultured to 80% confluence. The cells were then scraped and lysed in RIPA buffer and the lysates were centrifuged at 10,000 × *g* (4 °C for 10 min). Protein concentrations were determined using a BCA Protein Assay Kit (Thermo) according to the manufacturer’s instructions. A total of 40 µg protein from each sample was denatured, loaded into a well in a polyacrylamide gel, separated by SDS-PAGE, and transferred to a polyvinylidene difluoride membrane (Millipore, Billerica, MA). The membranes were blocked with 5% nonfat milk at room temperature for one hour and incubated overnight with primary antibodies in PBST (1:1000). After the membranes were washed with PBST, they were incubated for 1 h at room temperature with the corresponding horseradish peroxidase-conjugated secondary antibody at the appropriate dilution and then washed three times with PBST. The protein bands on the membranes were detected using enhanced chemiluminescence (Amersham Corporation, Arlington Heights, IL, USA). Western blot analysis was performed using the following antibodies: CDK5RAP3 (ab157203, Abcam, 1:1000), CCL2 (MCP1) (ab214819, Abcam, 1:1000), CCR2 (ab203128, Abcam, 1:1000), NF-κB p65 (ab16502, Abcam, 0.5 µg/ml), NF-kB p65 (phospho S536) (ab16502, Abcam, 1:1000), MMP2 (ab92536, Abcam, 1:2000), E-cadherin (ab40772, Abcam, 1:2000), N-cadherin (ab76011, Abcam, 1:5000), vimentin (ab92547, Abcam, 1:1000), Snail (ab216347, Abcam, 1:1000), β-ACTIN (ab8226, Abcam, 1:2000), and GAPDH (ab8245, Abcam, 1:1000).

### Immunofluorescence assay

Cells grown on coverslips were rinsed with PBS and fixed with ice-cold 4% paraformaldehyde for 5 min at RT. Subsequently, the cells were blocked with 0.2% Triton X-100 for 30 minutes followed by 5% BSA for 1 h, washed for 30 min, and incubated with primary monoclonal antibodies against CDK5RAP3 and NF-κB p65 overnight at 4 °C. The next day, the coverslips were incubated for 1 h in a dark room with fluorescently conjugated secondary antibody (1:200). Furthermore, the coverslips were stained with DAPI (Vector Laboratories, Burlingame, CA, USA) for 5 min at 4 °C. Finally, a laser scanning confocal microscope (Leica, Germany) was used to observe the expression in cells.

### ELISA

ELISA was conducted according to the instructions. Concentrations of CCL2 (human), IL4 (human), IL10 (human), and MMP2 (human) in the culture supernatant of treated cells were measured with the use of a commercially available kit (CUSABIO).

### MTT assay

BGC-823 cells were seeded into 96-well plates (5 × 10^3^ cells per well) and cultured overnight. Subsequently, 10 μL of MTT solution (5 mg/ml; Solarbio, China) was added to each well at 24, 48, 72 and 96 h. Following incubation for 2 h, the absorbance was measured at 450 nm using a spectrophotometer (FLX800, Bio - TEK).

### Colony formation assay

BGC-823 cells were seeded into well plates (500 cells per well) and cultured for 14 days. The cells in every well were fixed with 4% paraformaldehyde for 30 min and then stained with 0.2% crystal violet at room temperature for 30 min. The number of cell colonies was counted.

### Transwell migration and invasion assay

The cell migration test was performed in 24-well Transwells (pore size 8 μm; Corning, USA) without Matrigel coating. Twenty-four-well Transwells precoated with Matrigel (Falcon 354,480; BD Biosciences, USA) were used for the cell invasion test. A total of 1 × 10^5^ cells in 500 μl RPMI 1640 containing 1% foetal bovine serum were added to the upper chamber, and 750 μl RPMI 1640 containing 10% foetal bovine serum was added to the lower chamber. After 48 h of incubation, Matrigel and the cells remaining in the upper chamber were removed with a cotton swab. The cells on the lower surface of the membrane were fixed with 4% paraformaldehyde and stained with 0.5% crystal violet. The cells were counted and photographed in five microscopic fields (200×). All experiments were performed in triplicate.

### Processing of genomic data and gene set enrichment analysis

We used publicly available data from TCGA, which was downloaded from the Genomic Data Commons (https://portal.gdc.cancer.gov) on 15 June 2020, and this download included clinical information and mRNA expression data. The mRNA expression data were presented as counts and were normalized with R software (version 4.0.0) and the “limma” package. Gene set enrichment analysis (GSEA) performed by the Molecular Signature Database (MSigDB) was used to identify the pathways that were significantly enriched in CDK5RAP3^low^ tumour samples. If a gene set had a positive enrichment score, the majority of its members had higher expression accompanied by a higher risk score, and the set was considered ‘enriched’. Specific information on the method used for the analysis can be found in Table S5.

### Statistical analysis

All data were processed using SPSS 25.0 (SPSS Inc. Chicago, IL) and R software (version 4.0.0). The cut-off value for CDK5RAP3 expression was the median value. Data are presented as the mean ± SD and were analysed using Student’s *t*-test or one-way ANOVA. We used the *χ*² test or Fisher’s exact test to compare categorical variables of clinical characteristics. The Kaplan–Meier method was used for overall survival analysis, and a log-rank test was used to compare differences. We defined the survival time of patients who were lost to follow-up as the time from surgery to the last follow-up time, and the survival time of patients who were still alive at the end of the study was defined as the time from surgery to the database deadline. Two-tailed *P* values < 0.05 indicated significant differences.

## Supplementary information

Figures S1-S17 and Tables S1-S5

Supplemental figure legends

Descriptions of Supplementary Material

Data S1

Predicted binding sites for IL4

Predicted binding sites for IL10

GSEA report for low CDK5RAP3

## Data Availability

The dataset analysed for this study is available from the corresponding author upon reasonable request.
