# Supplementary material for: CDK5RAP3 acts as a tumour suppressor in gastric cancer through the infiltration and polarization of tumour-associated macrophages
Source: Cancer Gene Ther. 2022 Aug 23;30(1):22–37. doi: 10.1038/s41417-022-00515-9 (PMC9842504; doi:10.1038/s41417-022-00515-9)
Supplement: Supplementary file 1 — Figures S1-S17 and Tables S1-S5 [file 41417_2022_515_MOESM1_ESM.pdf]

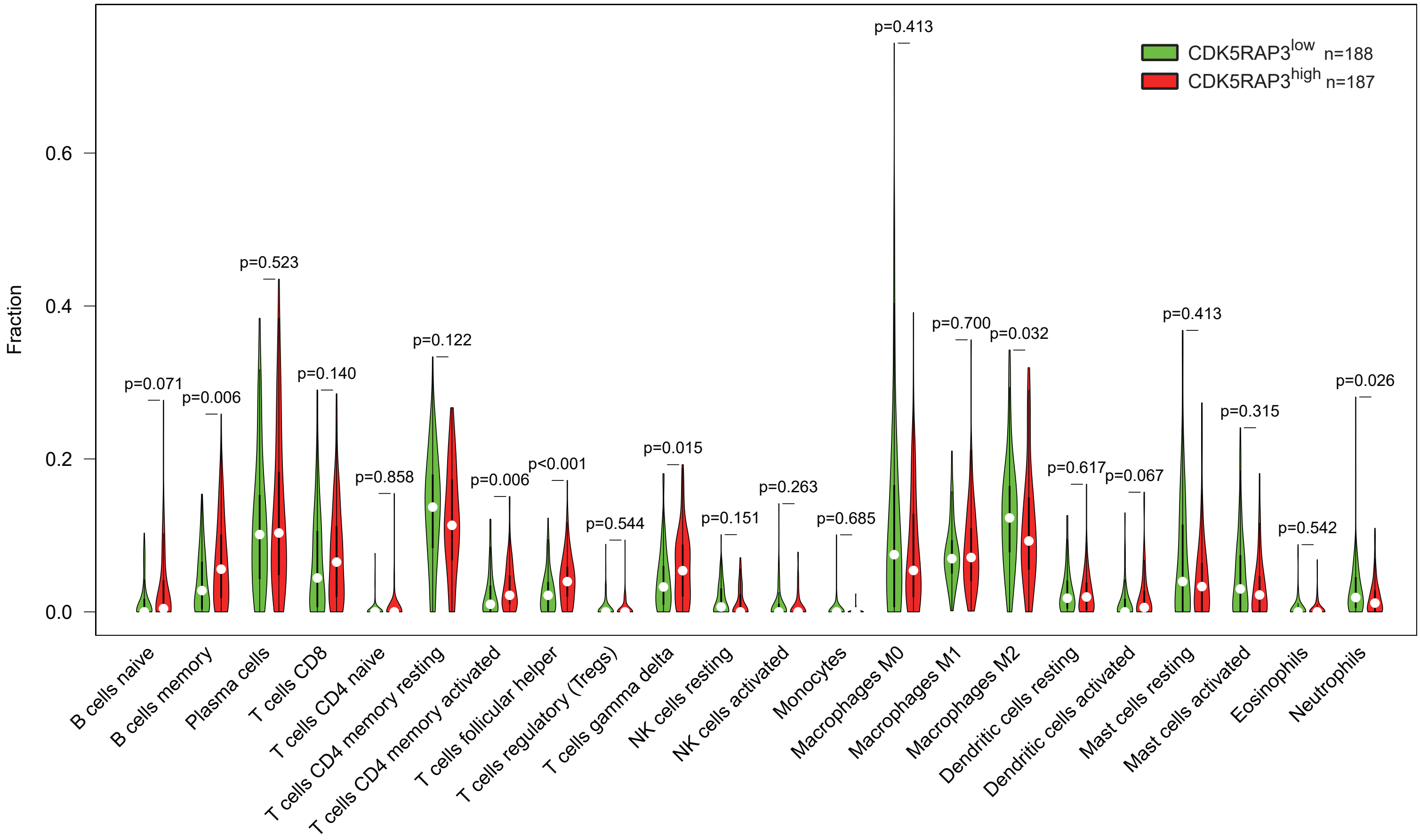

Figure S1 CIBERSORT analysis detects the infiltration of 22 kinds of immune cells in the tumour between the low subgroup of CDK5RAP3 and the high subgroup of CDK5RAP3. Gene expression level (9.97) of CDK5RAP3 was used to stratify tumors with low versus high CDK5RAP3 expression. Data are presented as the mean ± SD and were analysed using Wilcoxon's test. *P*-values were not adjusted for multiple comparisons.

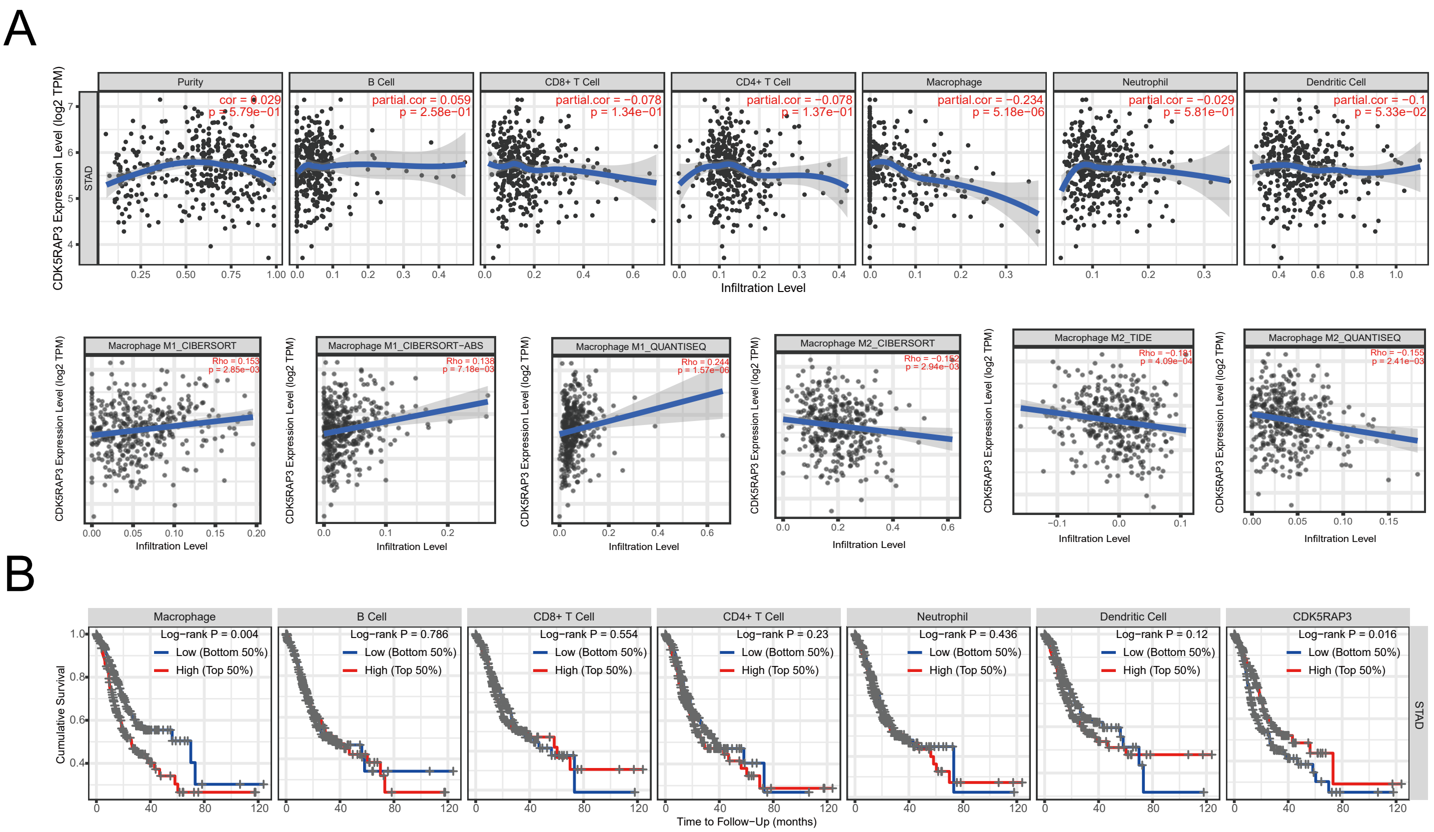

Figure S2 (A) The scatter plot shows the correlation between the proportion of infiltrating immune cells in STAD and the expression of CDK5RAP3. (B) Kaplan-Meier survival analysis of different immune infiltrating cells and CDK5RAP3 in the TIMER database. STAD, Stomach adenocarcinoma.

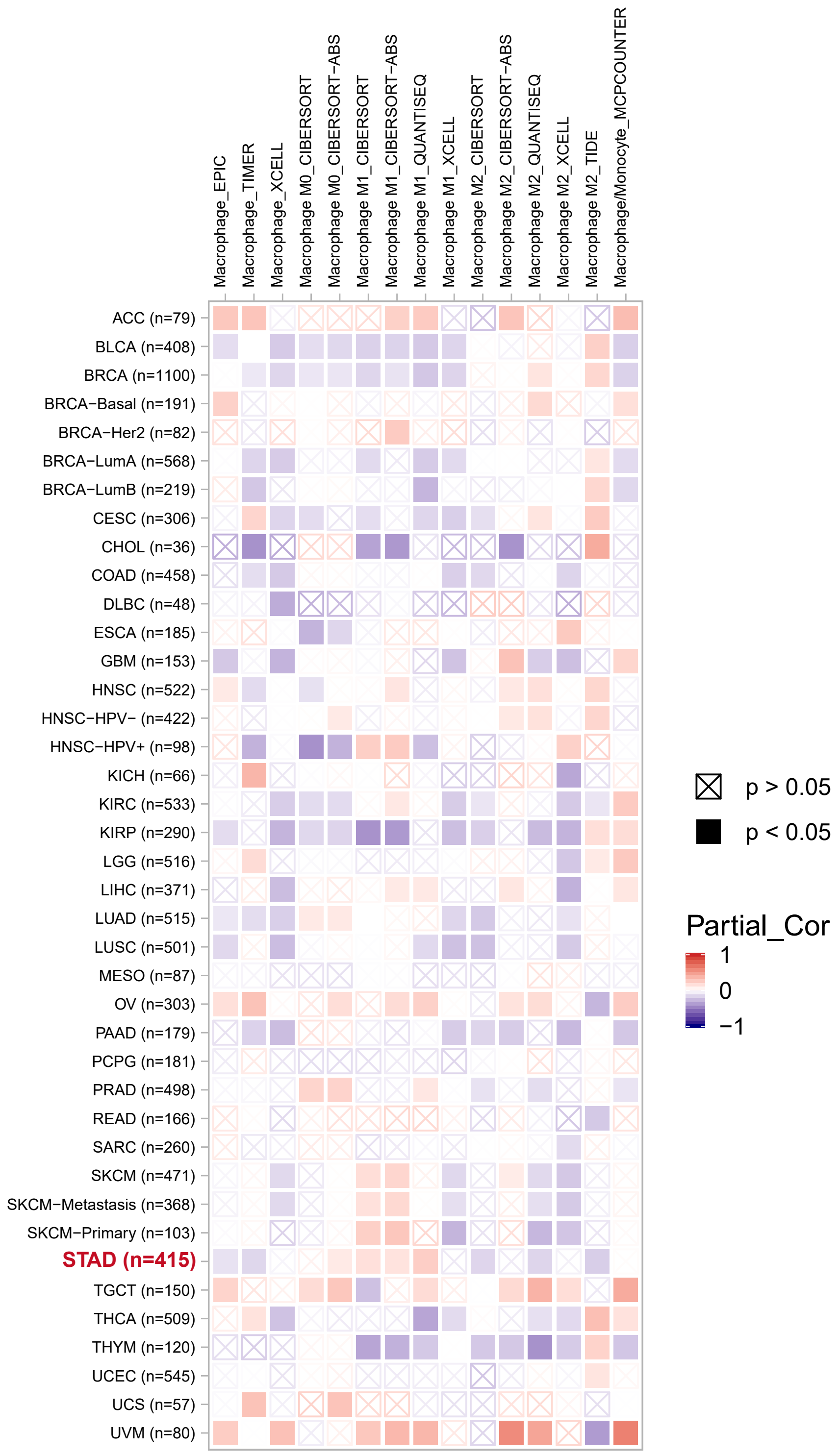

Figure S3 Visualize the correlation between the expression of CDK5RAP3 and the infiltration level of various types of macrophages in different cancer types in the TIMER database. The heatmap with numbers showed the purity-adjusted spearman's rho across various cancer types.

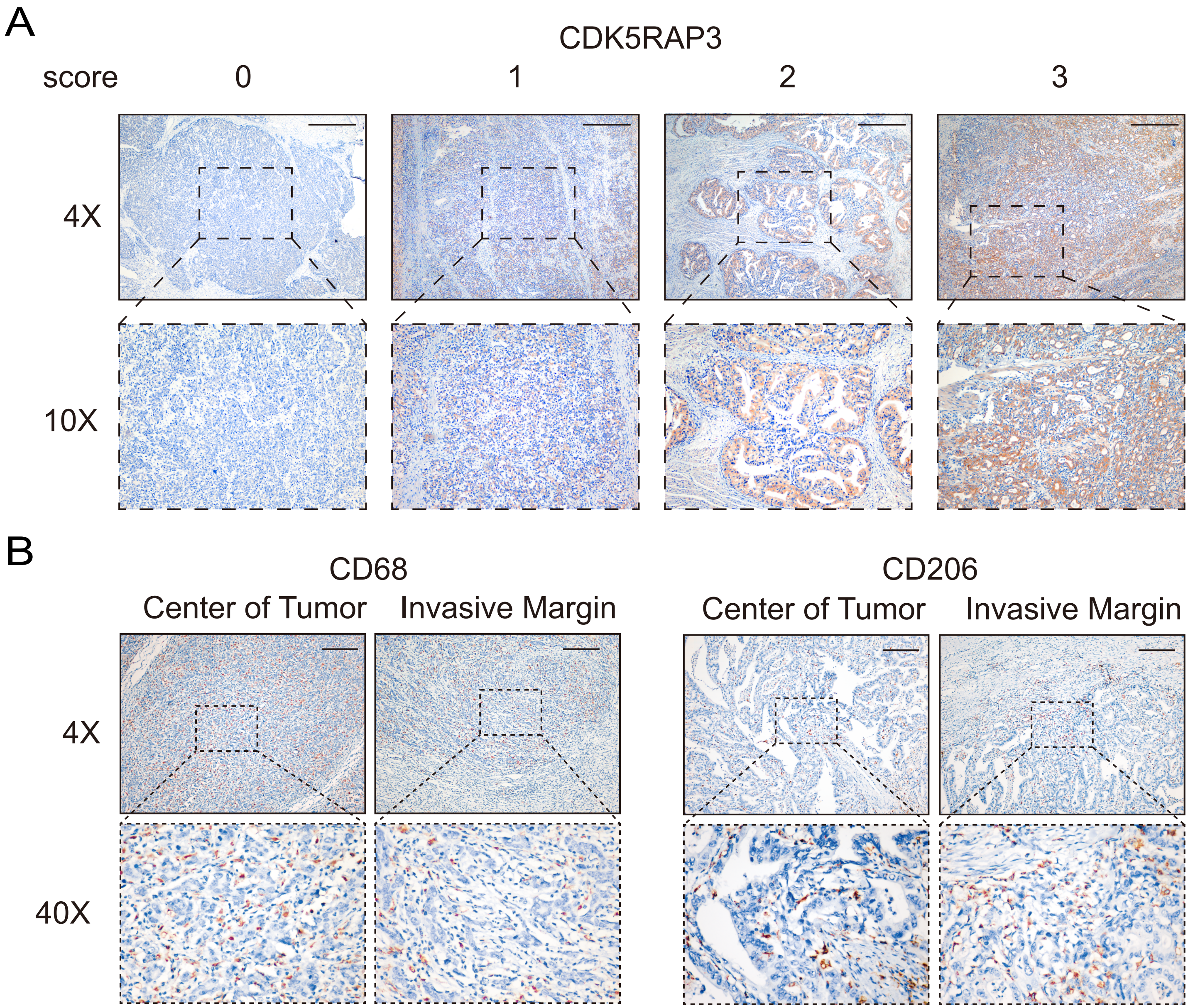

Figure S4 (A)Staining intensity scoring criteria for CDK5RAP3 IHC staining results in gastric tissue. Magnification: X4 and X10. Scale bar = 400 μm. (B) Immunohistochemical methods were used to detect the protein expression of CD68, and CD206 in CT and IM sections of gastric cancer tissues. The Representative photos are shown. Magnification: X4 and X40. Scale bars = 400 μm.

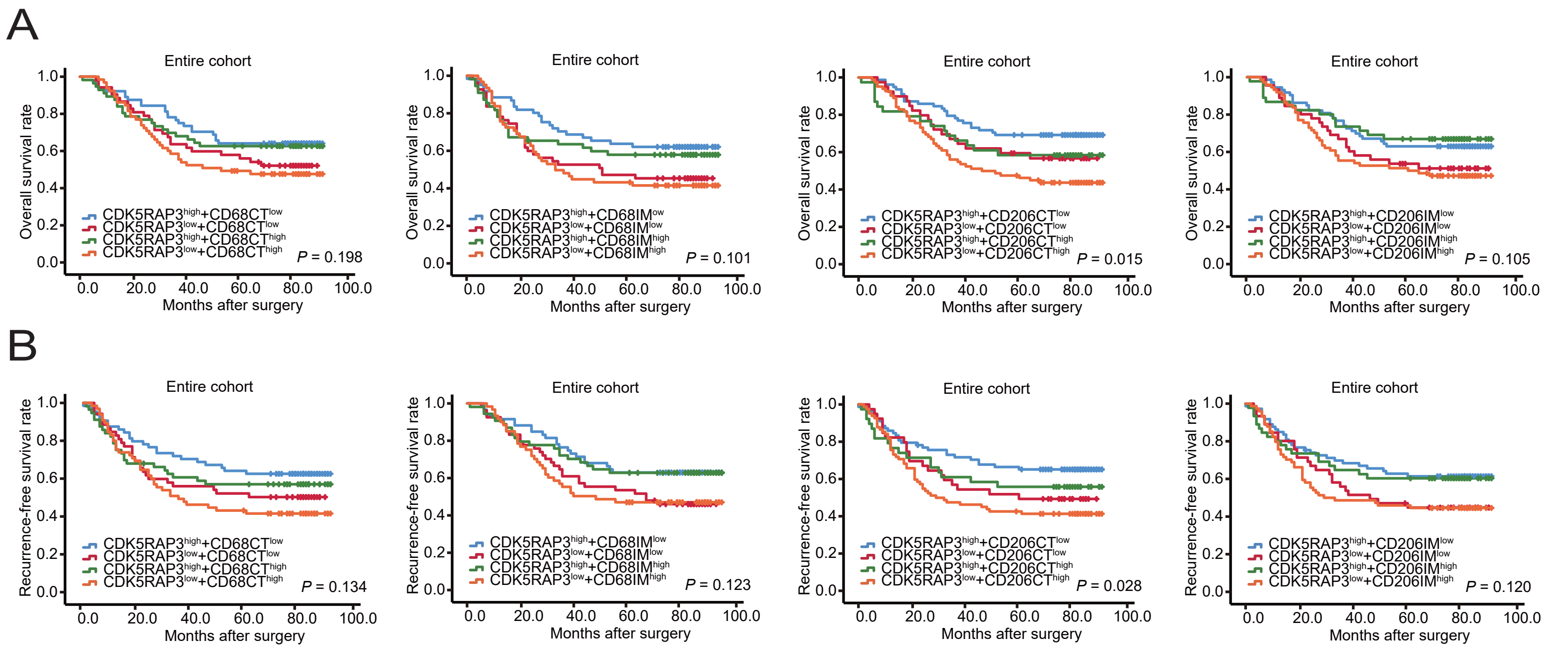

Figure S5 (A) Kaplan-Meier analysis of OS in gastric cancer patients based on the expression of CDK5RAP3 combined with CD68 and CD206 in gastric cancer tissues on CT and IM (n = 241). (B) Kaplan-Meier analysis of RFS in gastric cancer patients based on the expression of CDK5RAP3 combined with CD68 and CD206 in gastric cancer tissues on CT and IM (n = 241). *P*-values for all survival analyses were calculated using the log-rank test. *P*-values were used to compare statistical differences between all 4 patient subgroups.

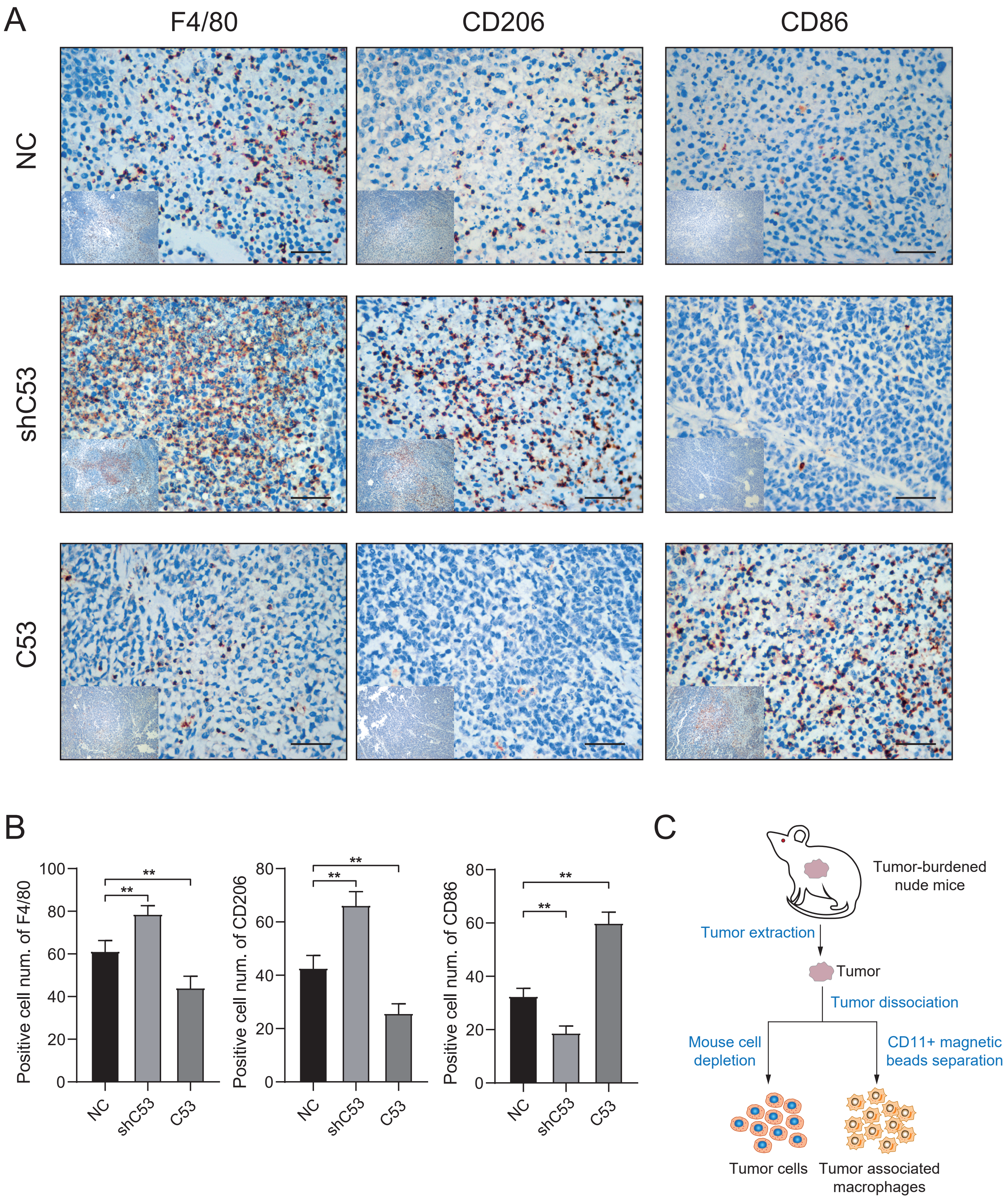

Figure S6 (A) F4/80, CD206 and CD86 immunohistochemical (IHC) images of tumours collected from Balb/c nude mice injected with stably transfected BGC-823 cells. Scale bar = 200 μm. N = 5 per group. (B) Count the results of IHC staining of F4/80, CD206 and CD86. *, *P* <0.05; **, *P* <0.01. (C) Schematic picture of the procedure for the separation of tumour cells and TAMs.

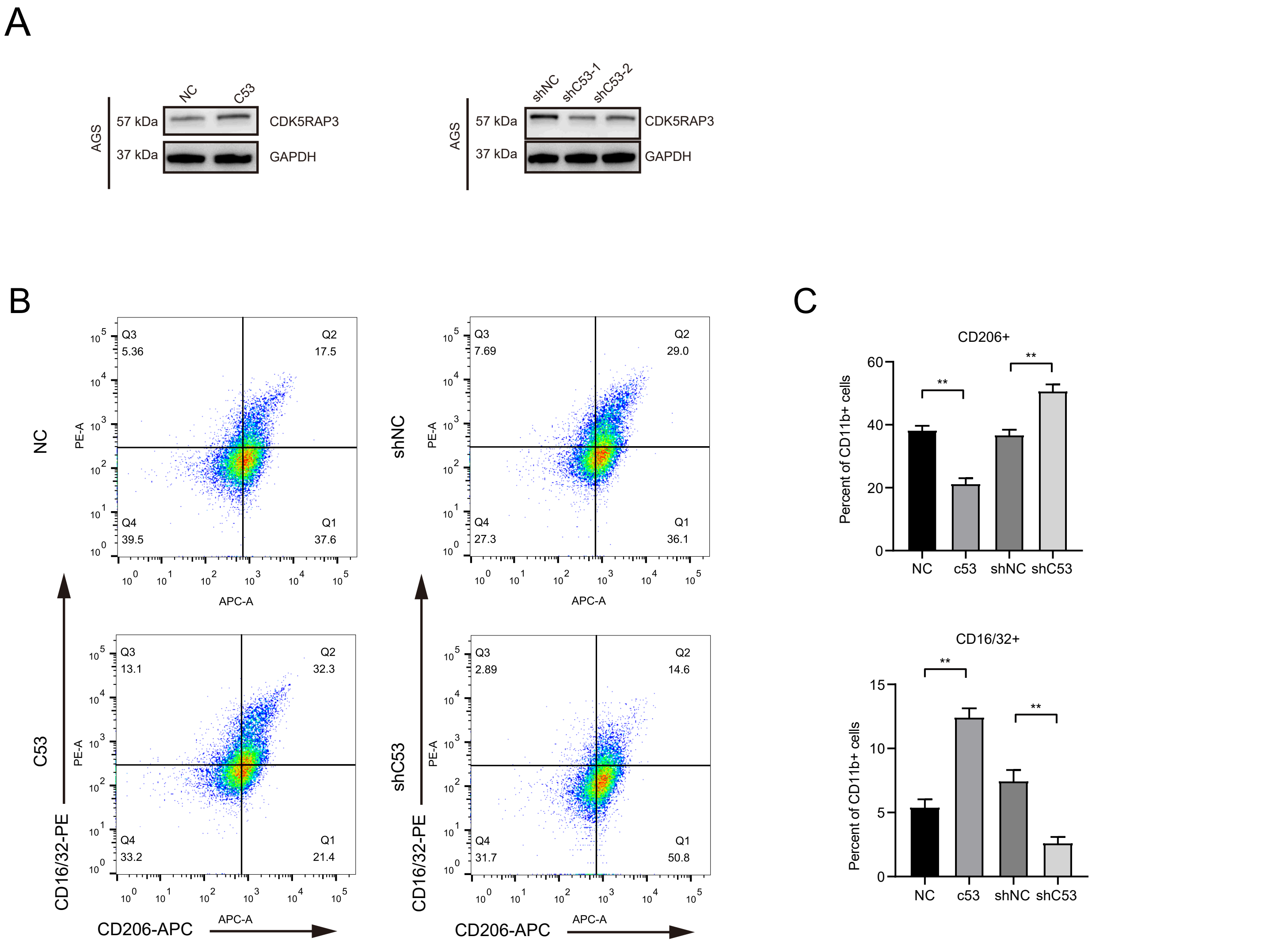

Figure S7 (A) Western blotting was used to detect the protein expression of CDK5RAP3 in AGS gastric cancer cell line. (B) Flow cytometry was used to detect the expression of CD16/32 and CD206 on the surface of CD11b+ macrophages and to determine the percentage of CD16/32+ and CD206+ cells in CD11b+ macrophages. (C) The bar graphs present the difference of Q3 quadrant (representing the percentage of CD16/32-PE staining positive cells) and Q1 quadrant (representing the percentage of CD206-APC staining positive cells) in different treatment groups in FACS plots.

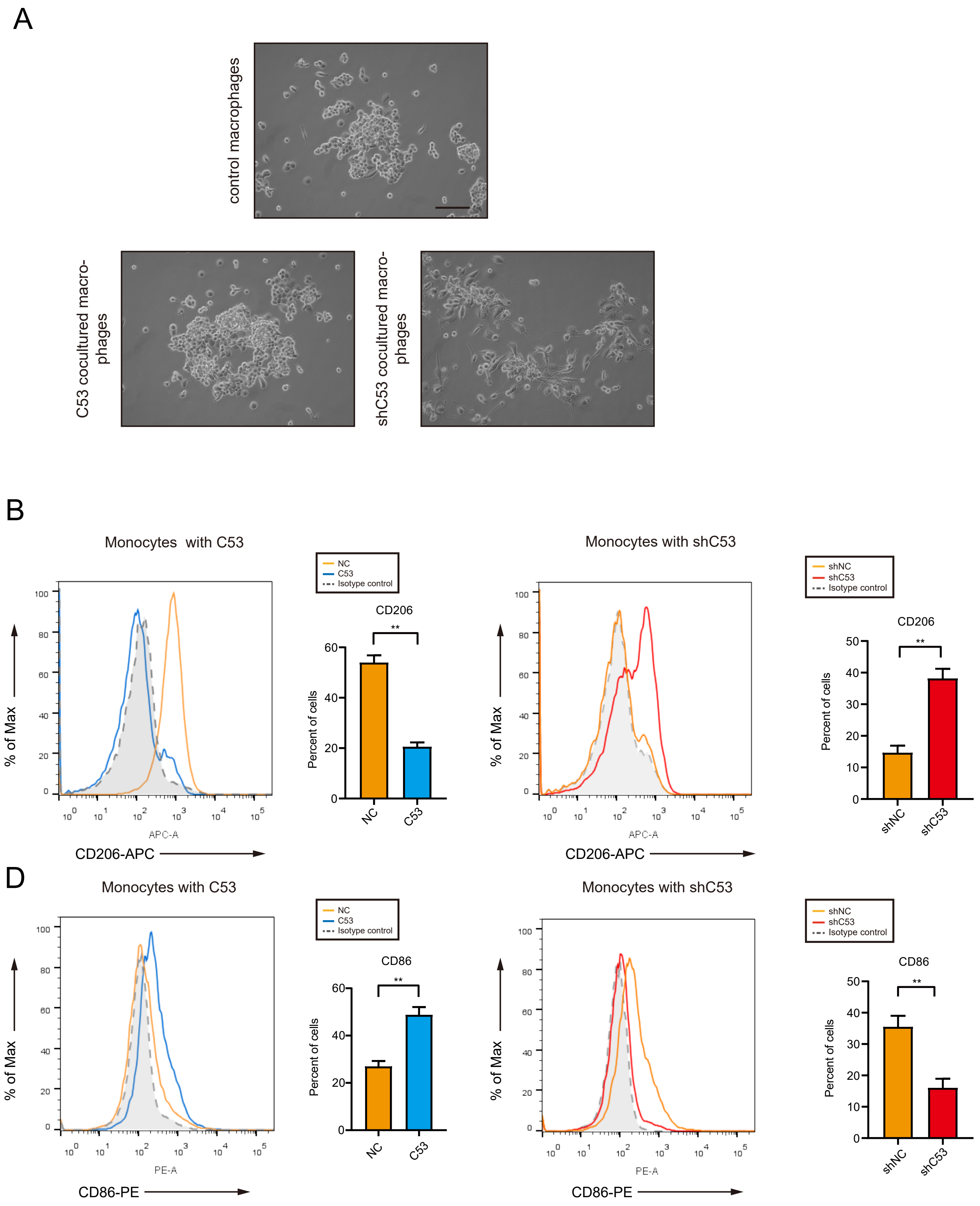

Figure S8 (A) Representative brightfield images of macrophages from each co-culture with the differently treated AGS gastric cancer cell lines are shown. Scale bar = 100 μm. (B) Flow cytometry was used to detect the expression of CD206 and CD86 on the surface of differentiated macrophages. *, P <0.05; **, P <0.01, compared with Vector.

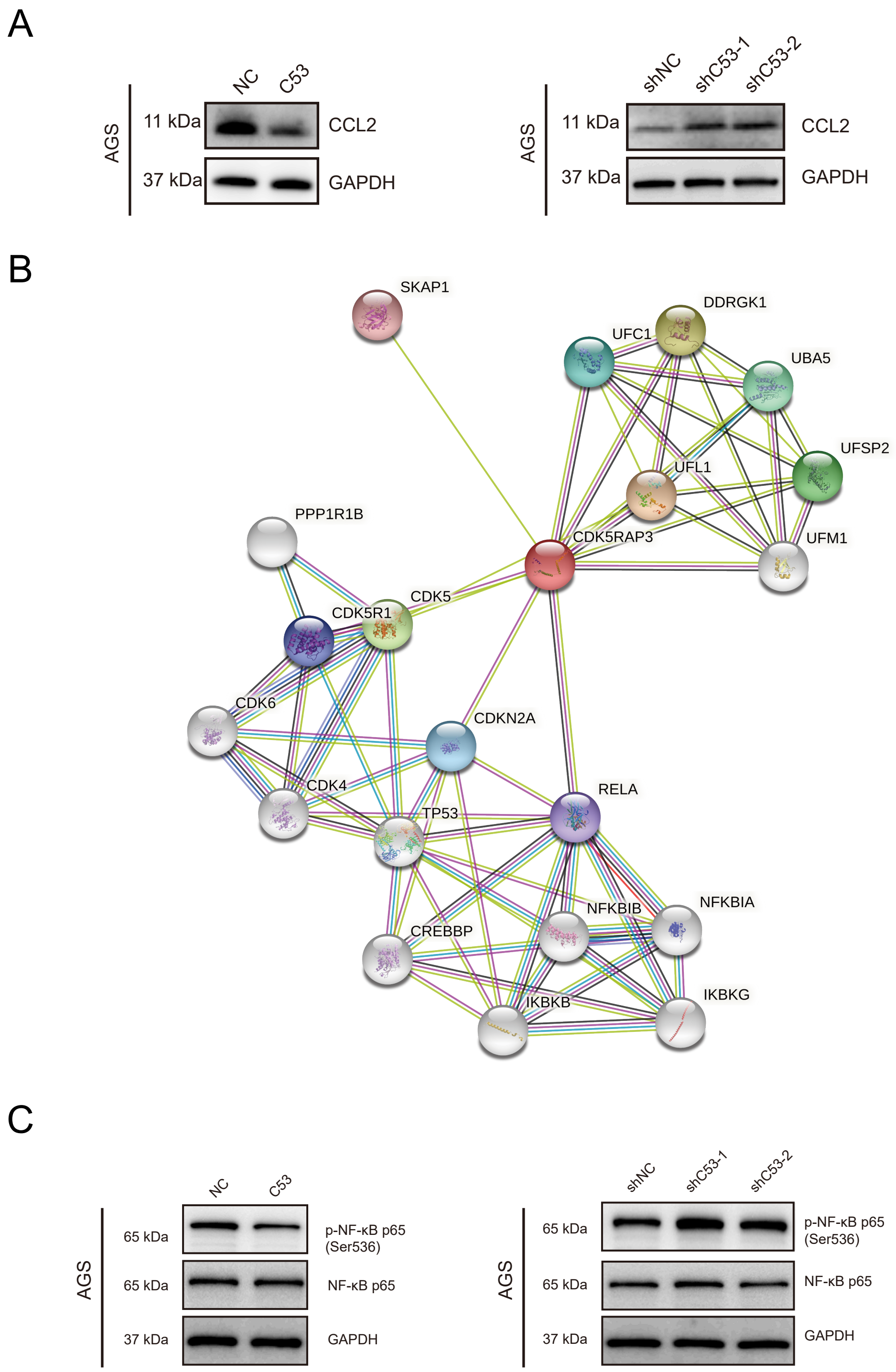

Figure S9 (A) Western blotting was used to detect the protein expression of CCL2 in the protein extract of the gastric cancer cell line AGS. (B) Combinations of CDK5RAP3 and NF-κB p65 (RELA) were presented using the STRING database based on gene expression data. (C) The expression of p-NF-κB p65 and NF-κB p65 in stably transfected AGS cell line was detected using Western blotting.

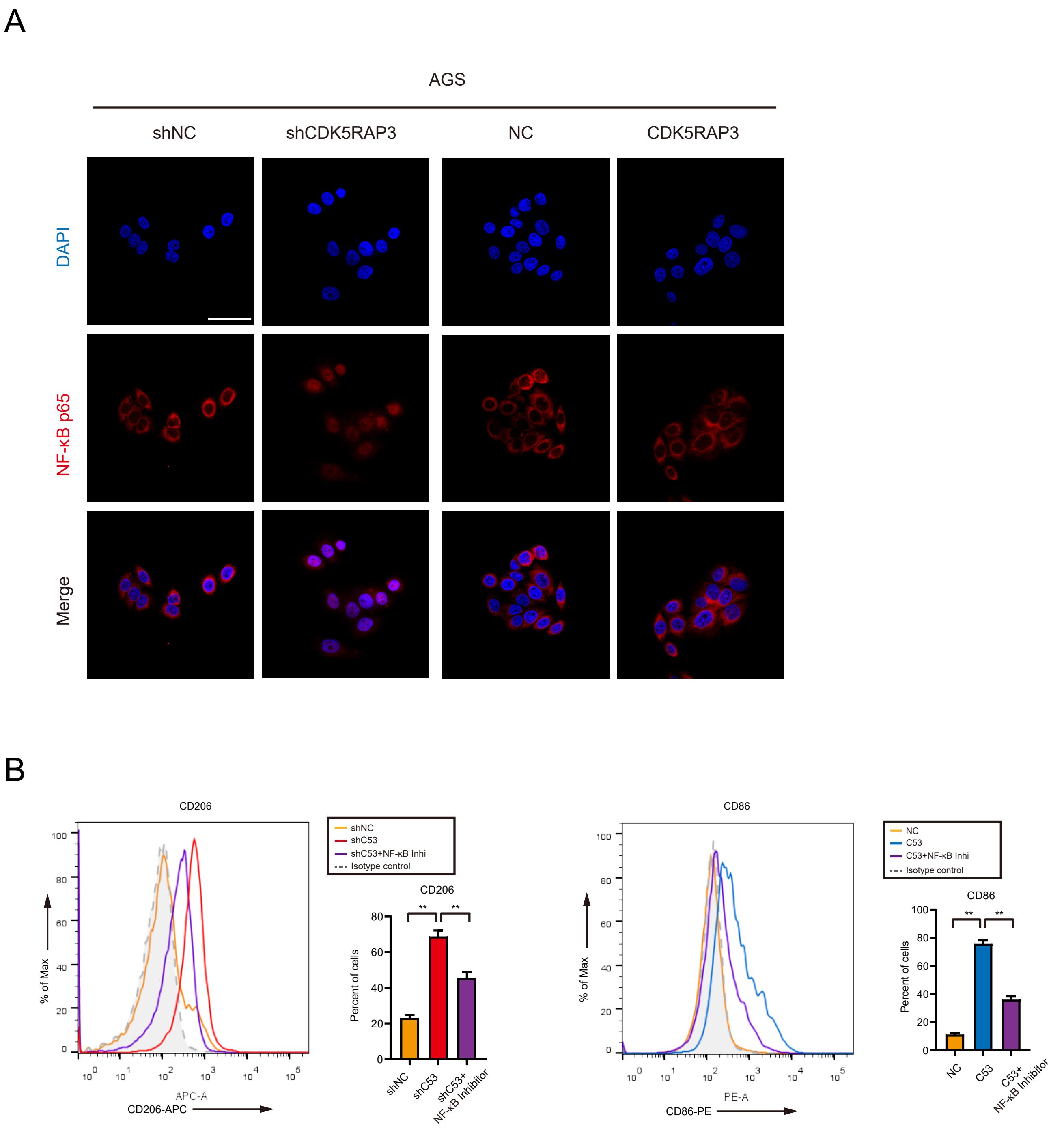

Figure S10 (A) Immunofluorescence (IF) staining showed the localization of NF-κB p65 (red) and DAPI (blue) in stably transfected AGS cells. Scale bar = 50μm. (B) Flow cytometry was used to detect the expression of CD206 and CD86 on the surface of differentiated macrophages. **, P <0.01, compared with Vector.

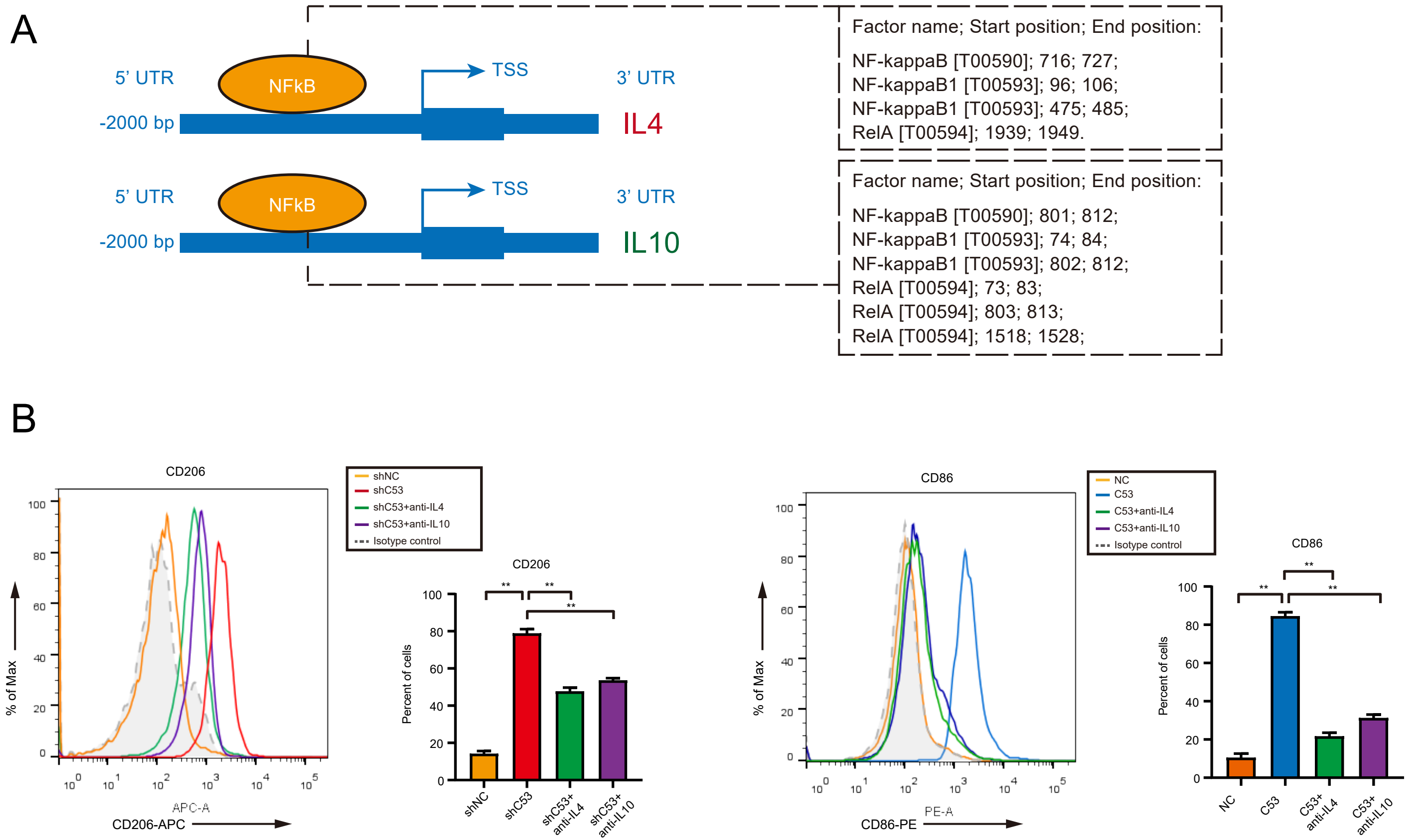

Figure S11 (A) Diagram shows the predicted binding site of NF-κB in the -2000 bp ~ +1 bp promoter regions of IL4 and IL10. (B) Flow cytometry was used to detect the expression of CD206 and CD86 on the surface of differentiated macrophages. **, P <0.01, compared with Vector.

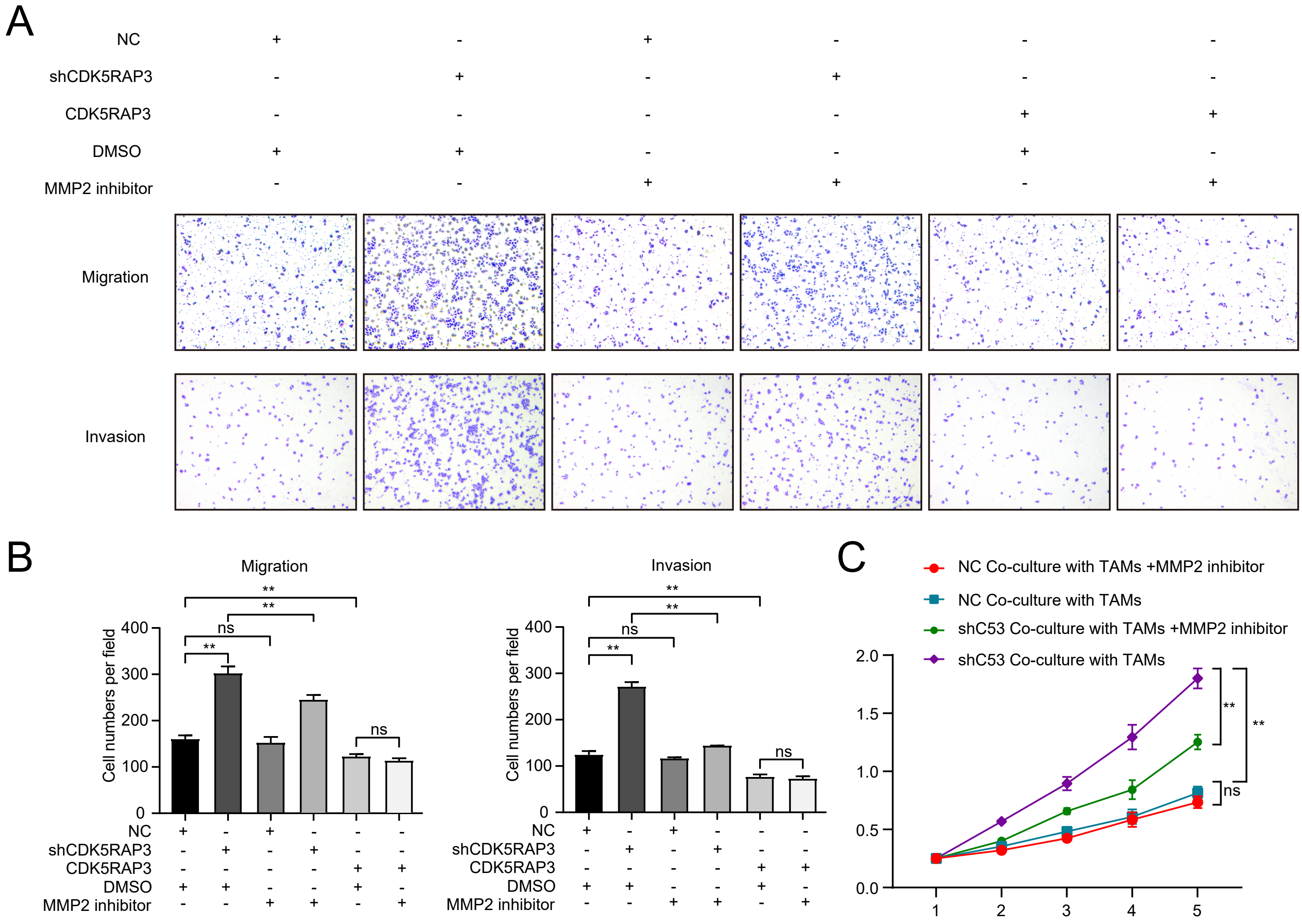

Figure S12 The invasion and migration of BGC-823 cells were observed in the Transwell system. All cells were tested after cocultivation with TAMs. A representative image is shown in (A), and the quantification is represented by the mean ± SD (*, *P* <0.05; **, *P* <0.01; ns, no significance) in (B). (C) The proliferative ability of BGC-823 cells in different groups was investigated via CCK-8 assays.

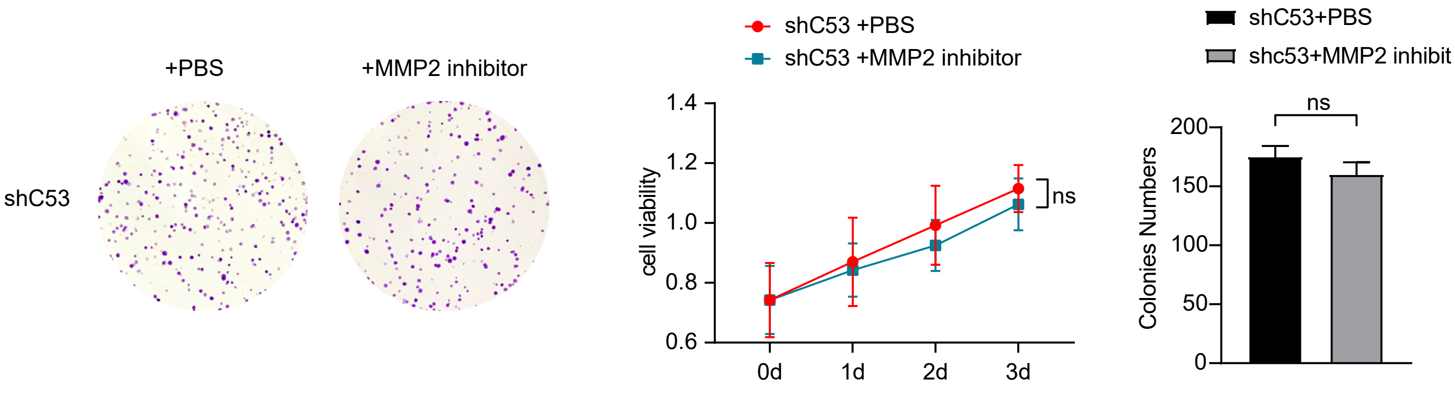

Figure S13 Colony formation assay and MTT assay of shCDK5RAP3 cells with MMP2 inhibitor applied or not.

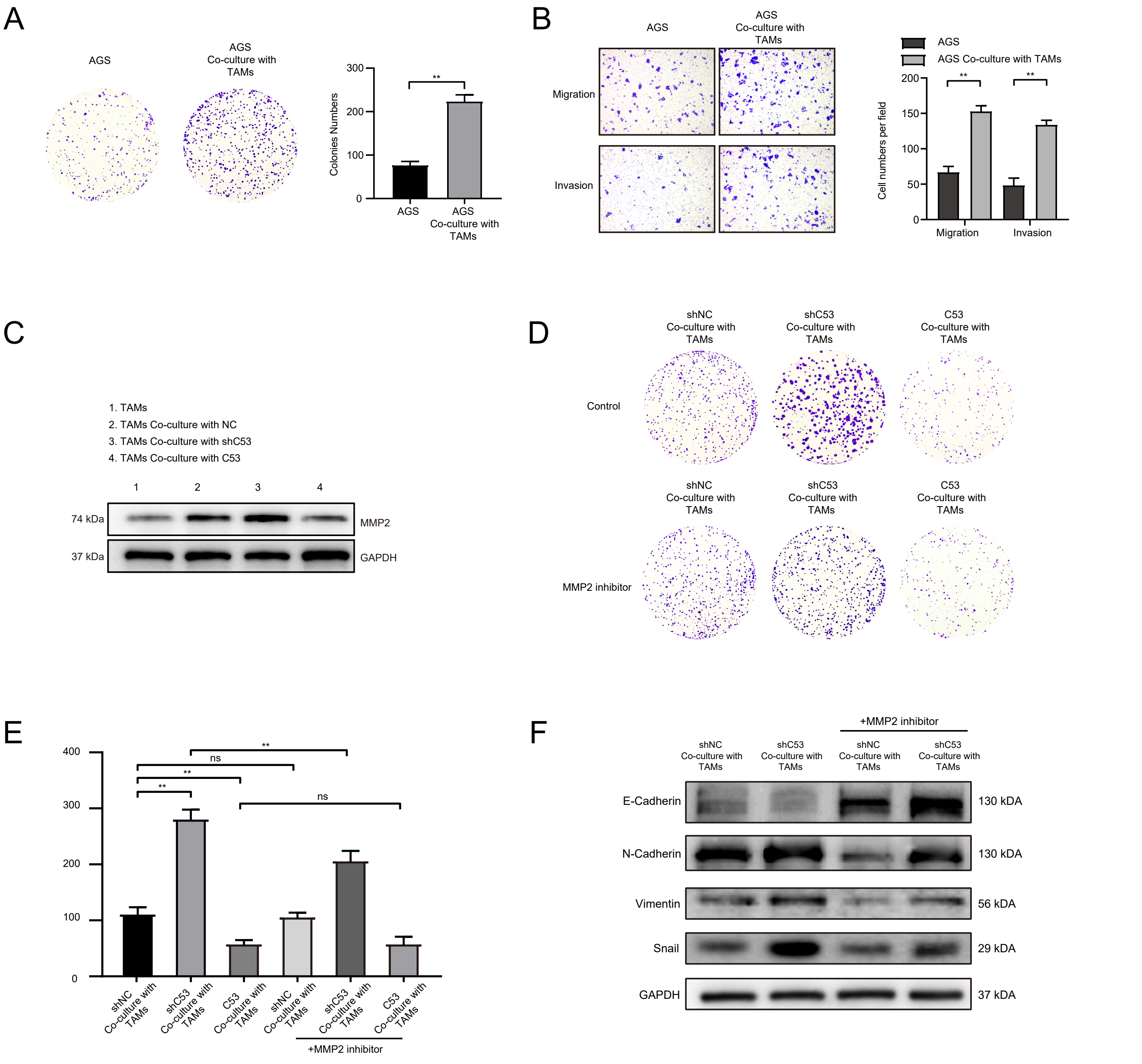

Figure S14 (A) Colony formation assay of AGS cells cocultured with macrophages or single cultures. Days = 8. (B) Transwell assays of stably transfected AGS cells were performed, and the quantification of the results is presented. Days = 8. (C) Western blotting were used to determine MMP2 expression of macrophages in different coculture systems. Coculture time = 48 hours. (D, E) Colony formation assay of AGS cells in different treatment coculture systems. Days = 8. (F) Western blotting was used to detect the expression of E-cadherin, N-cadherin, vimentin and Snail in AGS cells in different treatment coculture systems.

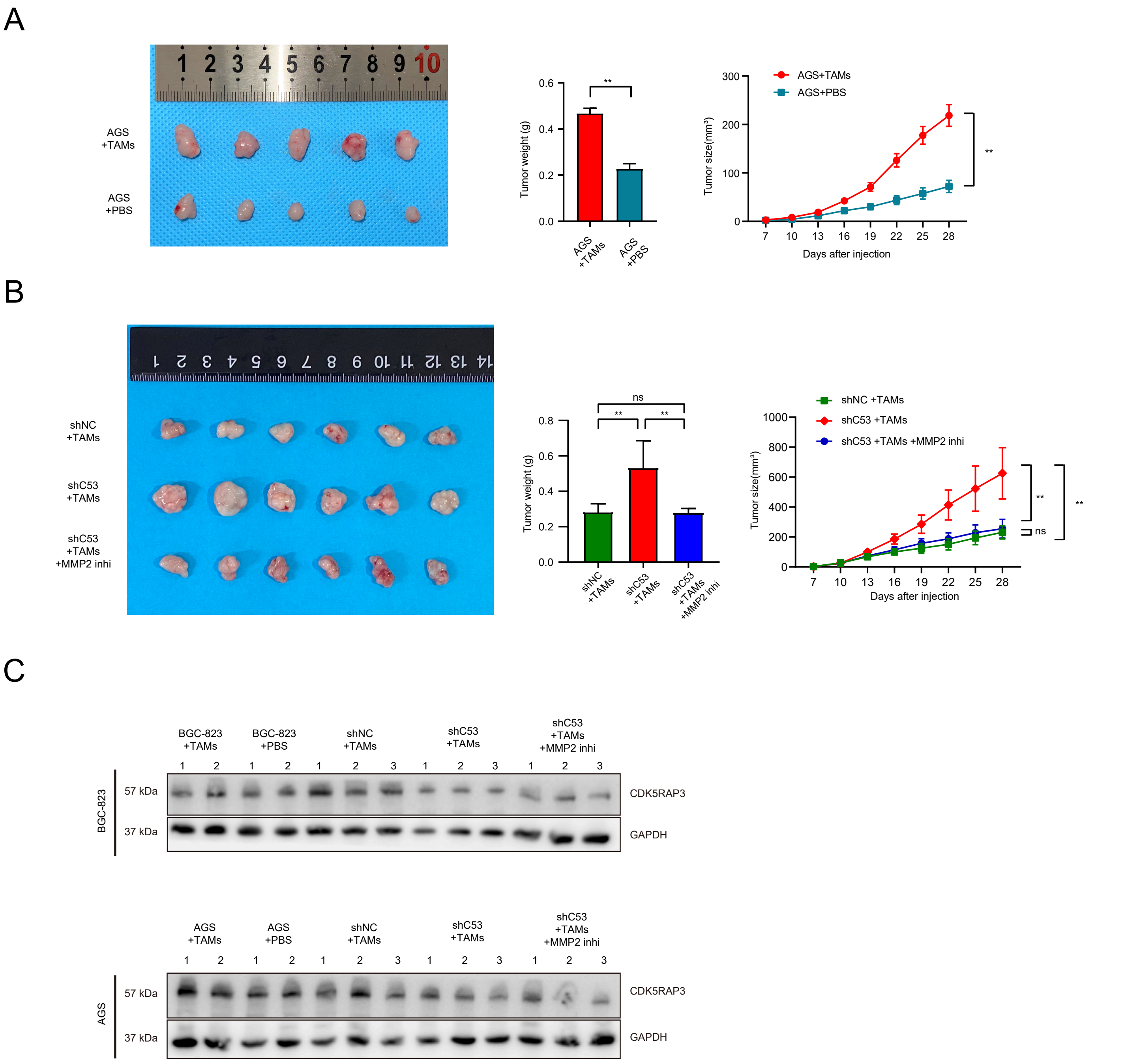

Figure S15 (A, B) Representative pictures of tumours in each group of mice are shown. (n = 6 per group). The average tumour weights of the three different groups were compared. The size of the xenograft was measured every 3 days until the mice were sacrificed. The xenograft tumour model used the AGS gastric cancer cell line. (C) Western blotting was used to detect the expression of CDK5RAP3 in xenograft tumours in different groups.

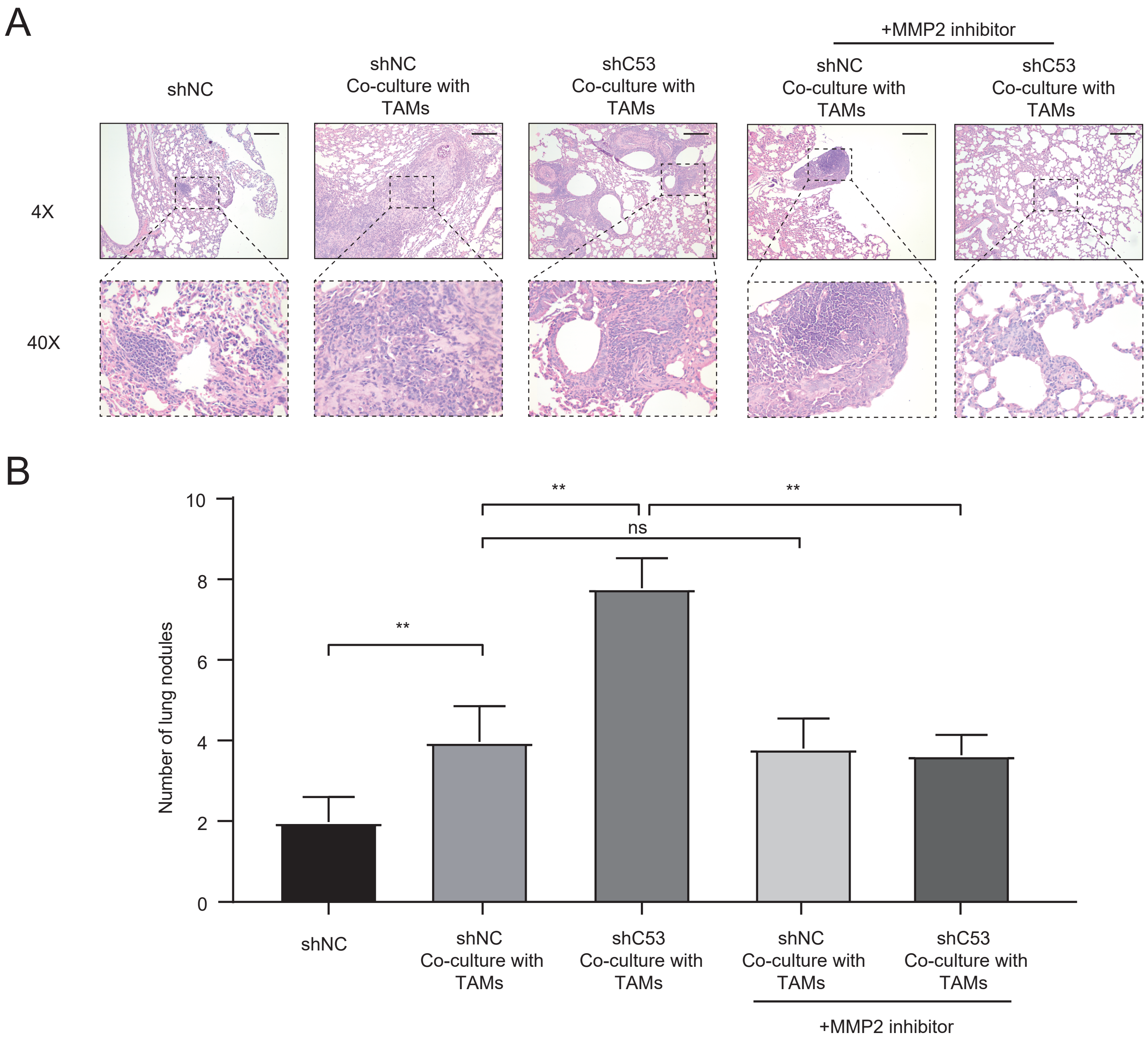

Figure S16 (A) Representative images of lung metastasis and haematoxylin-and-eosin staining. Scale bars = 200 μm. The lung metastasis model used the BGC-823 gastric cancer cell line. (B) Comparison of the number of lung nodules in lung metastasis models in different groups (n=6 per group).

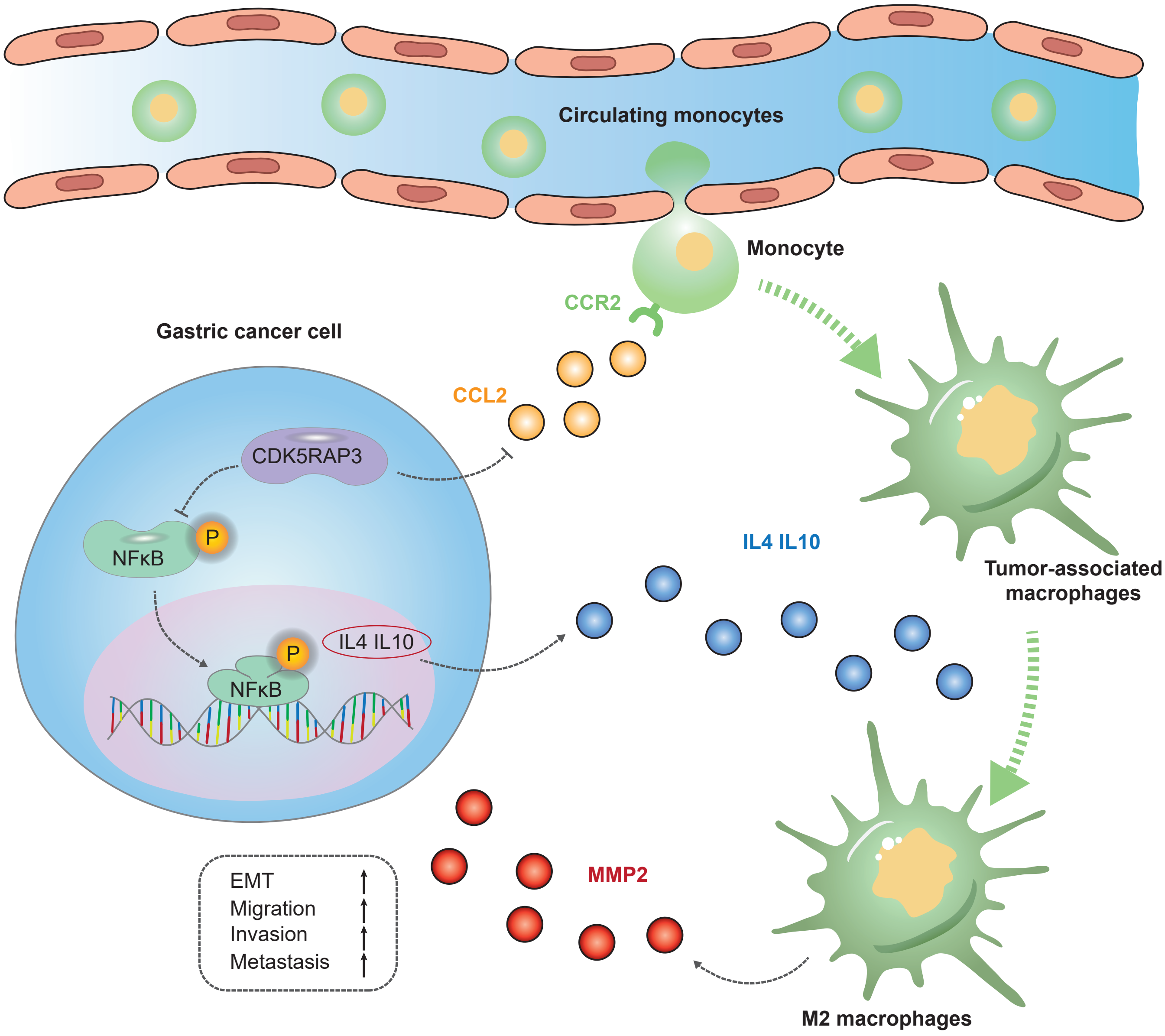

Figure S17 Graphical model of the relationship between CDK5RAP3 expression in gastric cancer and its immunoregulatory effect on tumour-associated macrophages in tumour microenvironment.

Table S1. Relationship Between TAMs Expression and Baseline Characteristics of Patients

variables	CD68 IM			CD68 CT			CD206 IM			CD206 CT		
	low	high	*P*	low	high	*P*	low	high	*P*	low	high	*P*
**Gender**			1.000			1.000			0.221			0.579
Male	90	90		90	30		85	95		92	88	
Female	30	31		30	31		35	26		28	33	
**Age at surgery**			0.166			0.549			0.262			1.000
>65	44	56		47	53		45	55		50	50	
≤65	76	65		73	68		75	66		70	71	
**BMI**			**0.033**			0.153			0.170			0.977
>25	26	13		24	15		15	24		20	19	
≤25	94	108		96	106		105	97		100	102	
**Tumor size (mm)**			0.176			0.107			1.000			1.000
>45	55	54		54	68		61	61		61	61	
≤45	65	67		66	53		59	60		59	60	
**Location of**			0.240			0.469			0.246			0.696
Lower 1/3	25	32		26	31		28	29		32	25	
Middle 1/3	18	21		16	23		17	22		20	19	
Upper 1/3	64	49		61	52		63	50		53	60	
More than 1/3	13	19		17	15		12	20		15	17	
**Chemotherapy***			0.637			1.000			0.462			0.637
No	46	51		48	49		45	52		46	51	
Yes	74	70		72	72		75	69		74	70	
**Differentiation**			0.923			**0.016**			0.114			**0.016**
Well/moderate	36	38		46	28		43	31		46	28	
Poor and not	84	83		74	93		77	90		74	93	
**Depth of invasion**			**0.022**			**0.008**			0.246			0.803
T1	23	8		23	8		14	17		14	17	
T2	12	11		12	11		8	15		12	11	
T3	48	51		51	48		48	51		47	52	
T4	37	51		34	54		50	39		47	41	
**Lymph node**			0.219			0.732			0.961			0.246
N0	37	26		34	29		31	32		33	30	
N1	16	25		22	19		22	19		23	18	
N2	23	28		23	28		25	26		25	26	
N3	44	42		41	45		42	44		39	47	
**TNM stage**			**0.027**			0.066			0.694			0.469
I	27	12		26	13		17	22		18	21	
II	27	35		30	32		32	30		35	27	
III	66	74		64	76		71	69		67	73	

IM: invasive margin; CT: center of tumour.

$P < 0.05$ marked in bold font shows statistical significance.

*Adjuvant chemotherapy after surgery. No radiotherapy was administered to anyone of the patients.

Table S2. Relationship Between CDK5RAP3 Expression and TAMs Expression of Patients

variables	Total	CDK5RAP3 expression			
		low	high	*χ2*	***P***
**CD68 invasive margin**				0.502	0.529
Low expression	120	57	63		
High expression	121	64	57		
**CD68 center of tumor**				1.500	0.549
Low expression	120	55	65		
High expression	121	66	55		
**CD206 invasive margin**				15.55	**0.000**
Low expression	120	46	74		
High expression	121	75	46		
**CD206 center of tumor**				25.894	**0.000**
Low expression	120	40	80		
High expression	121	81	40		

$P < 0.05$ marked in bold font shows statistical significance.

Table S3. GSEA details of KEGG_CYTOKINE_CYTOKINE_RECEPTOR_INTERACTION interaction pathway

NAME	PROBE	RANK IN GENE LIST	RANK METRIC SCORE	RUNNING ES	CORE ENRICHMENT
row_0	ACVRL1	45	0.376545	0.01556	Yes
row_1	GHR	277	0.322436	0.025386	Yes
row_2	HGF	367	0.30878	0.037199	Yes
row_3	CXCL12	406	0.302708	0.049675	Yes
row_4	IL1R1	422	0.301109	0.062499	Yes
row_5	TNFSF12	441	0.298498	0.075156	Yes
row_6	IL17B	472	0.294041	0.0874	Yes
row_7	VEGFC	585	0.281252	0.097598	Yes
row_8	TGFB1	592	0.280423	0.109686	Yes
row_9	PDGFRB	702	0.26741	0.119336	Yes
row_10	CCL11	722	0.264949	0.130515	Yes
row_11	LEPR	750	0.261941	0.141418	Yes
row_12	PDGFRA	753	0.261684	0.152764	Yes
row_13	TGFB3	887	0.248855	0.16117	Yes
row_14	TGFBR1	1003	0.236861	0.169382	Yes
row_15	IL3RA	1201	0.220724	0.175401	Yes
row_16	CX3CR1	1216	0.219483	0.184694	Yes
row_17	PLEKHO2	1265	0.215307	0.193186	Yes
row_18	IL4	1368	0.206593	0.200318	Yes
row_19	CSF1R	1574	0.191784	0.204933	Yes
row_20	NGFR	1742	0.181129	0.209776	Yes
row_21	VEGFB	1772	0.179474	0.217055	Yes
row_22	CCL14	1780	0.179041	0.224715	Yes
row_23	TSLP	1806	0.177299	0.231973	Yes
row_24	BMPR1A	1816	0.17654	0.239488	Yes
row_25	TPO	1884	0.172547	0.245775	Yes
row_26	PDGFB	1919	0.17111	0.2526	Yes
row_27	KIT	2036	0.165469	0.257688	Yes
row_28	FLT4	2080	0.163669	0.264026	Yes
row_29	BMPR1B	2157	0.159751	0.269593	Yes
row_30	INHBA	2208	0.157136	0.275519	Yes
row_31	IL6	2224	0.156261	0.282043	Yes
row_32	TGFBR2	2356	0.152069	0.286276	Yes
row_33	MPL	2418	0.149435	0.291667	Yes
row_34	IL10	2490	0.146354	0.296742	Yes
row_35	TGFB2	2594	0.142542	0.301069	Yes
row_36	BMPR2	2603	0.142227	0.30711	Yes
row_37	CCL2	2644	0.140843	0.312509	Yes

row_38	CCR4	2855	0.134203	0.314529	Yes
row_39	CXCR1	2903	0.132919	0.319456	Yes
row_40	IL1A	3014	0.129422	0.323085	Yes
row_41	OSM	3106	0.126965	0.326954	Yes
row_42	CXCR2	3160	0.125346	0.331442	Yes
row_43	CSF1	3217	0.123634	0.335802	Yes
row_44	PRL	3337	0.120237	0.338868	Yes
row_45	IL24	3370	0.119514	0.343485	Yes
row_46	TNFRSF4	3392	0.119092	0.348283	Yes
row_47	CCL3	3420	0.118257	0.352936	Yes
row_48	PDGFC	3430	0.117991	0.357905	Yes
row_49	CCL23	3472	0.117017	0.362249	Yes
row_50	GDF5	3648	0.113093	0.363987	Yes
row_51	CSF3R	3744	0.111131	0.367094	Yes
row_52	CSF2RB	3773	0.110419	0.371388	Yes
row_53	CCL26	3952	0.107083	0.372809	Yes
row_54	KDR	3967	0.106814	0.377201	Yes
row_55	FLT3LG	4036	0.105591	0.380557	Yes
row_56	EDA2R	4151	0.10368	0.382995	Yes
row_57	IL1B	4163	0.103527	0.387298	Yes
row_58	CSF2	4311	0.101246	0.389029	Yes
row_59	CCL25	4395	0.099707	0.391857	Yes
row_60	IL9R	4466	0.098641	0.394875	Yes
row_61	CXCR3	4532	0.097653	0.397941	Yes
row_62	CCR9	4578	0.096889	0.401337	Yes
row_63	CCL4	4841	0.093312	0.400633	Yes
row_64	FLT1	4910	0.092216	0.403407	Yes
row_65	TNFSF11	4999	0.091076	0.405769	Yes
row_66	CCL22	5113	0.089479	0.407607	Yes
row_67	CSF3	5178	0.088667	0.4103	Yes
row_68	CCL7	5484	0.084854	0.408446	Yes
row_69	IL5	5763	0.082096	0.406962	Yes
row_70	IL13	5793	0.081787	0.409993	Yes
row_71	CCL19	5898	0.080528	0.411605	Yes
row_72	TNFRSF1A	5935	0.080214	0.414439	Yes
row_73	AMHR2	6292	0.076709	0.411304	Yes
row_74	CCL16	6336	0.076358	0.413843	Yes
row_75	CCL17	6407	0.07577	0.415866	Yes
row_76	CTF1	6613	0.07395	0.415356	Yes
row_77	CCR10	6632	0.073773	0.418237	Yes
row_78	PRLR	6961	0.071136	0.415369	Yes
row_79	CCR3	7001	0.070867	0.417742	Yes
row_80	LEP	7078	0.070277	0.419417	Yes
row_81	IL5RA	7110	0.07007	0.421901	Yes

row_82	CCR1	7259	0.068906	0.422208	Yes
row_83	IL4R	7454	0.067501	0.421617	Yes
row_84	CCL8	7516	0.067109	0.423427	Yes
row_85	GH2	7774	0.065042	0.421584	Yes
row_86	CSF2RA	7855	0.064449	0.422933	Yes
row_87	CCL3L1	7891	0.064109	0.425085	Yes

Table S4. Sequence of primers for qRT-PCR.

Names	Sequences
Tnf (mouse)	Forward: 5′-CCTCTCATGCACCACCATCA-3′
	Reverse: 5′-GCATTGCACCTCAGGGAAGA-3′
Inos (mouse)	Forward: 5′-GCAGAATGTGACCATCATGG-3′
	Reverse: 5′-GCAGAATGTGACCATCATGG-3′
Il1b (mouse)	Forward: 5′-AAGGGGACATTAGGCAGCAC-3′
	Reverse: 5′-ATGAAAGACCTCAGTGCGGG-3′
Cxcl9 (mouse)	Forward: 5′-GCAGTGTGGAGTTCGAGGAA-3′
	Reverse: 5′-AGTCCGGATCTAGGCAGGTT-3′
Il12a (mouse)	Forward: 5′-CTCAGTTTGGCCAGGGTCAT-3′
	Reverse: 5′-TCTTCAGCAGGTTTCGGGAC-3′
Arg1(mouse)	Forward: 5′-AACCATCTGGGGCATCACAG-3′
	Reverse: 5′-ACCAGAAAGGAACTGCTGGG-3′
Tgfb1 (mouse)	Forward: 5′-GTCCAAACTAAGGCTCGCCA-3′
	Reverse: 5′-ATAGATGGCGTTGTTGCGGT-3′
Il6 (mouse)	Forward: 5′-GACTGGGGATGTCTGTAGCTC-3′
	Reverse: 5′-CACCAGCATCAGTCCCAAGA-3′
Il10 (mouse)	Forward: 5′-GCATGGCCCAGAAATCAAGG-3′
	Reverse: 5′-AATCGATGACAGCGCCTCAG-3′
Ccl22 (mouse)	Forward: 5′-CCCTATGGTGCCAATGTGGA-3′
	Reverse: 5′-GCAAGGCTCTTGCTGGAATG-3′
TNF (human)	Forward: 5′-AGAACTCACTGGGGCCTACA-3′
	Reverse: 5′-GCTCCGTGTCTCAAGGAAGT-3′
INOS (human)	Forward: 5′-GAGCGAGTTGTGGATTGTC-3′
	Reverse: 5′-CTCCTTTGAGCCCTTTGT-3′
IL1B (human)	Forward: 5′-GAGCTCGCCAGTGAAATGAT-3′
	Reverse: 5′-CCTGAAGCCCTTGCTGTAGT-3′
ARG1(human)	Forward: 5′-GTCTGTGGGAAAAGCAAGCG-3′
	Reverse: 5′-CACCAGGCTGATTCTTCCGT-3′
TGFB1 (human)	Forward: 5′-TGAGCCAGAGGCGGACTACT-3′
	Reverse: 5′-TGAGCCAGAGGCGGACTACT-3′
IL6 (human)	Forward: 5′-TCTCAACCCCCAATAAATAT-3′
	Reverse: 5′-GATGCCGTCGAGGATGTACC-3′
CCL2	Forward: 5′-TTCCCCTAGCTTTCCCCAGA-3′
	Reverse: 5′-TCCCAGGGGTAGAACTGTGG-3′
E-cadherin	Forward: 5′-CTGCAGGTCTCATCATGGA-3′
	Reverse: 5′-ACCTGTAGACCTCGGCACTG-3′
N-cadherin	Forward: 5′-CCGTGAATGGGCAGATCACT-3′
	Reverse: 5′-TAGGCGGGATTCCATTGTCA-3′
Vimentin	Forward: 5′-GAGAACTTTGCCGTTGAAGC-3′
	Reverse: 5′-GCTTCCTGTAGGTGGCAATC-3
Snail	Forward: 5′-TCAGACGAGGACAGTGGGAAAG-3′
	Reverse: 5′-GCTTGTGGAGCAGGGACATTC-3′

MMP2	Forward: 5′-CTTCCAAGTCTGGAGCGATGT-3′
	Reverse: 5′-TACCGTCAAAGGGGTATCCAT-3′
IL4	Forward: 5′-ACACAACTGAGAAGGAAACC-3′
	Reverse: 5′-ATGA TCGTCTTTAGCCTTTC-3′
IL10 (human)	Forward: 5′-ATGCCCCAAGCTGAGAACCA-3′
	Reverse: 5′-TCTCAAGGGGCTGGGTCAGC-3′
β-actin	Forward: 5′-CTGTCCCTGTATGCCTCTG-3′
	Reverse: 5′-ATGTCACGCACGATTTCC-3′

Table S5. The tools used in our study

TOOLS	SOURCE	WEBSITE LINKS
**CIBERSORT**	Newman et al[1]. (2015)	https://cibersort.stanford.edu/
**GSEA**	Subramanian et al[2]. (2005)	http://software.broadinstitute.org/gsea
**TIMER v2.0**	Taiwen Li et al[3]. (2020)	http://timer.cistrome.org/
**STRING v2021**	Szklarczyk D et al[4]. (2021)	https://cn.string-db.org/
**JASPAR v2020**	Fornes O et al[5]. (2020)	https://jaspar.genereg.net/
**PROMO**	Messeguer X et al[6]. (2002)	http://alggen.lsi.upc.es/cgi-bin/promo_v3/promo/promoinit.cgi?dirDB=TF_8.3

References:

1. Newman, A.M., Liu, C.L., Green, M.R., Gentles, A.J., Feng, W., Xu, Y., Hoang, C.D., Diehn, M., and Alizadeh, A.A. (2015). Robust enumeration of cell subsets from tissue expression profiles. Nat Methods *12*, 453-457. 10.1038/nmeth.3337.
2. Subramanian, A., Tamayo, P., Mootha, V.K., Mukherjee, S., Ebert, B.L., Gillette, M.A., Paulovich, A., Pomeroy, S.L., Golub, T.R., Lander, E.S., and Mesirov, J.P. (2005). Gene set enrichment analysis: a knowledge-based approach for interpreting genome-wide expression profiles. Proc Natl Acad Sci U S A *102*, 15545-15550. 10.1073/pnas.0506580102.
3. Li, T., Fu, J., Zeng, Z., Cohen, D., Li, J., Chen, Q., Li, B., and Liu, X.S. (2020). TIMER2.0 for analysis of tumor-infiltrating immune cells. Nucleic Acids Res *48*, W509-W514. 10.1093/nar/gkaa407.
4. Szklarczyk, D., Gable, A.L., Nastou, K.C., Lyon, D., Kirsch, R., Pyysalo, S., Doncheva, N.T., Legeay, M., Fang, T., Bork, P., Jensen, L.J., et al. (2021). The STRING database in 2021: customizable protein-protein networks, and functional characterization of user-uploaded gene/measurement sets. Nucleic Acids Res *49*, D605-D612. 10.1093/nar/gkaa1074.
5. Fornes, O., Castro-Mondragon, J.A., Khan, A., van der Lee, R., Zhang, X., Richmond, P.A., Modi, B.P., Correard, S., Gheorghe, M., Baranasic, D., Santana-Garcia, W., et al. (2020). JASPAR 2020: update of the open-access database of transcription factor binding profiles. Nucleic Acids Res *48*, D87-D92. 10.1093/nar/gkz1001.
6. Messeguer, X., Escudero, R., Farre, D., Nunez, O., Martinez, J., and Alba, M.M. (2002). PROMO: detection of known transcription regulatory elements using species-tailored searches. Bioinformatics *18*, 333-334. 10.1093/bioinformatics/18.2.333.
