# Supplementary material for: CDK5RAP3 acts as a tumour suppressor in gastric cancer through the infiltration and polarization of tumour-associated macrophages
Source: Cancer Gene Ther. 2022 Aug 23;30(1):22–37. doi: 10.1038/s41417-022-00515-9 (PMC9842504; doi:10.1038/s41417-022-00515-9)
Supplement: Supplementary file 2 — Supplemental figure legends [file 41417_2022_515_MOESM2_ESM.docx]

Supplemental figure legends

Figure S1. CIBERSORT analysis detects the infiltration of 22 kinds of immune cells in the tumour between the low subgroup of CDK5RAP3 and the high subgroup of CDK5RAP3. Gene expression level (9.97) of CDK5RAP3 was used to stratify tumours with low versus high CDK5RAP3 expression. Data are presented as the mean ± SD and were analysed using Wilcoxon's test. *P*-values were not adjusted for multiple comparisons.

Figure S2. (A) The scatter plot shows the correlation between the proportion of infiltrating immune cells in STAD and the expression of CDK5RAP3. (B) Kaplan-Meier survival analysis of different immune infiltrating cells and CDK5RAP3 in the TIMER database.

STAD, Stomach adenocarcinoma.

Figure S3. Visualize the correlation between the expression of CDK5RAP3 and the infiltration level of various types of macrophages in different cancer types in the TIMER database. The heatmap with numbers showed the purity-adjusted spearman's rho across various cancer types.

Figure S4. (A) Staining intensity scoring criteria for CDK5RAP3 IHC staining results in gastric tissue. Magnification: X4 and X10. Scale bar = 400 µm. (B) Immunohistochemical methods were used to detect the protein expression of CD68, and CD206 in CT and IM sections of gastric cancer tissues. The Representative photos are shown. Magnification: X4 and X40. Scale bars = 400 µm.

Figure S5. (A) Kaplan-Meier analysis of OS in gastric cancer patients based on the expression of CDK5RAP3 combined with CD68 and CD206 in gastric cancer tissues on CT and IM (n = 241). (B) Kaplan-Meier analysis of RFS in gastric cancer patients based on the expression of CDK5RAP3 combined with CD68 and CD206 in gastric cancer tissues on CT and IM (n = 241). *P*-values for all survival analyses were calculated using the log-rank test. *P*-values were used to compare statistical differences between all 4 patient subgroups.

Figure S6. (A) F4/80, CD206 and CD86 immunohistochemical (IHC) images of tumours collected from Balb/c nude mice injected with stably transfected BGC-823 cells. Scale bar = 50 μm. N = 5 per group. (B) Count the results of IHC staining of F4/80, CD206 and CD86. *, *P* <0.05; **, *P* <0.01. (C) Schematic picture of the procedure for the separation of tumour cells and TAMs.

Figure S7. (A) Western blotting was used to detect the protein expression of CDK5RAP3 in AGS gastric cancer cell line. (B) Flow cytometry was used to detect the expression of CD16/32 and CD206 on the surface of CD11b+ macrophages and to determine the percentage of CD16/32+ and CD206+ cells in CD11b+ macrophages. (C) The bar graphs present the difference of Q3 quadrant (representing the percentage of CD16/32-PE staining positive cells) and Q1 quadrant (representing the percentage of CD206-APC staining positive cells) in different treatment groups in FACS plots.

Figure S8. (A) Representative brightfield images of macrophages from each co-culture with the differently treated AGS gastric cancer cell lines are shown. Scale bar = 100 μm. (B) Flow cytometry was used to detect the expression of CD206 and CD86 on the surface of differentiated macrophages. *, P <0.05; **, P <0.01, compared with Vector.

Figure S9. (A) Western blotting was used to detect the protein expression of CCL2 in the protein extract of the gastric cancer cell line AGS. (B) Combinations of CDK5RAP3 and NF-κB p65 (RELA) were presented using the STRING database based on gene expression data. (C) The expression of p-NF-κB p65 and NF-κB p65 in stably transfected AGS cell line was detected using Western blotting.

Figure S10. (A) Immunofluorescence (IF) staining showed the localization of NF-κB p65 (red) and DAPI (blue) in stably transfected AGS cells. Scale bar = 50μm. (B) Flow cytometry was used to detect the expression of CD206 and CD86 on the surface of differentiated macrophages. **, P <0.01, compared with Vector.

Figure S11. (A) Diagram shows the predicted binding site of NF-κB in the -2000 bp ~ +1 bp promoter regions of IL4 and IL10. (B) Flow cytometry was used to detect the expression of CD206 and CD86 on the surface of differentiated macrophages. **, P <0.01, compared with Vector.

Figure S12. The invasion and migration of BGC-823 cells were observed in the Transwell system. All cells were tested after cocultivation with TAMs. A representative image is shown in (A), and the quantification is represented by the mean ± SD (*, *P* <0.05; **, *P* <0.01; ns, no significance) in (B). (C) The proliferative ability of BGC-823 cells in different groups was investigated via CCK-8 assays.

Figure S13. Colony formation assay and MTT assay of shCDK5RAP3 cells with MMP2 inhibitor applied or not.

Figure S14. (A) Colony formation assay of AGS cells cocultured with macrophages or single cultures. Days = 8. (B) Transwell assays of stably transfected AGS cells were performed, and the quantification of the results is presented. Days = 8. (C) Western blotting were used to determine MMP2 expression of macrophages in different coculture systems. Coculture time = 48 hours. (D, E) Colony formation assay of AGS cells in different treatment coculture systems. Days = 8. (F) Western blotting was used to detect the expression of E-cadherin, N-cadherin, vimentin and Snail in AGS cells in different treatment coculture systems.

Figure S15. (A, B) Representative pictures of tumours in each group of mice are shown. (n = 6 per group). The average tumour weights of the three different groups were compared. The size of the xenograft was measured every 3 days until the mice were sacrificed. The xenograft tumour model used the AGS gastric cancer cell line. (C) Western blotting was used to detect the expression of CDK5RAP3 in xenograft tumours in different groups.

Figure S16. (A) Representative images of lung metastasis and haematoxylin-and-eosin staining. Scale bars = 200 μm. The lung metastasis model used the BGC-823 gastric cancer cell line. (B) Comparison of the number of lung nodules in lung metastasis models in different groups (n=6 per group).

Figure S17. Graphical model of the relationship between CDK5RAP3 expression in gastric cancer and its immunoregulatory effect on tumour-associated macrophages in tumour microenvironment.
