# Supplementary material for: CDK5RAP3 acts as a tumour suppressor in gastric cancer through the infiltration and polarization of tumour-associated macrophages
Source: Cancer Gene Ther. 2022 Aug 23;30(1):22–37. doi: 10.1038/s41417-022-00515-9 (PMC9842504; doi:10.1038/s41417-022-00515-9)
Supplement: Supplementary file 3 — Descriptions of Supplementary Material [file 41417_2022_515_MOESM3_ESM.docx]

**Descriptions of Supplementary Material**

**Data S1 Description:**

The survival and clinicopathological data and the Immunohistochemical score of 241 gastric cancer patients.

**Predicted binding sites：**

The different NF-kB binding sites from 2000bp upstream of the transcription start point of IL4 and IL10 genes to the transcription start point.

**GSEA report for low CDK5RAP3:**

KEGG enrichments results for "KEGG CYTOKINE CYTOKINE RECEPTOR INTERACTION" pathway when CDK5RAP3 is low expressed.
