# Supplementary material for: CDK5RAP3 acts as a tumour suppressor in gastric cancer through the infiltration and polarization of tumour-associated macrophages
Source: Cancer Gene Ther. 2022 Aug 23;30(1):22–37. doi: 10.1038/s41417-022-00515-9 (PMC9842504; doi:10.1038/s41417-022-00515-9)
Supplement: Supplementary file 5 — Predicted binding sites for IL4 [file 41417_2022_515_MOESM5_ESM.html]

searchRes.txt
-- Input sequence ---------------------------------------------------
  
>Sequence
  
GGCATGGTGGTTCACGCCTATAATCCCAGCACTTTGGGAGGCTGAGGTGGGTGGATCATTTTGAGGCCAGGAGTTCGACACCAGCCTAGCCAACATGGTGAAACCCCATCTCTACTAAAAAAAAAAAAAAAATACAGAAATTAGCCAGGCGTGGTGGCATGTGCCTGTAATCCCAGCTACTCGGGAAGCTGAGGCTGGAGAATGCCTTGAGCCCAGGAGATCAAGGCTACAGTAAGCTATGATCACCACTGCACTCCAGCCTGGGTGACAGAGTATGGGGGCAGGGGGTGGTGAGGGGGGGGCGGGGAAGTGGAACAGAGGCAAAACCTTAGCAACACACATTTTTAGATGATCTTCCAGAATAAATTCATAGGGAGGCCCAGGCACAGTGGCTCACGCCTGTAATCCCAGCACTTTGGGAGGCCGAGGCAGGCGGATCACGAGGTCAGGAGATGGAGACCATCCTGGCTAACACGGTGAAACCCCGTCTCTACTAAAAATACAAAAAATTAGCCGGGCGTGGTGGCAGGTGCCTGTAGTCCCAGCTACTCGGGGAGGCTGAGGCAGGAGAACGGCATGAACCCAGGAGGCGGAGCTTGCAGTGAACTAAGATCACGCCACTGCACTCCAGCCTGGGTGACAGAGCAAGATTCCATCTCAAAAAAAAAAAAAAAAAAAAGAAATTCATAGGGAAAAGAAGGTCAGAGACCAAGGGAAGGGAAGGTTCTGGGAGAAAAGCAGGGGGCAGGCAGGGCCCAAGAATCCTGCTGCCCATGAGCCCTTACTGGGAGGTGGGGTGGCCTTGCACAGGGCCCAGGCACCTGAGTGAGTGGTGGGGTCCTTACGTTCACTGCTGGGGTGAGGCAATGAGCACCTTATTGTGTCCACATGAATTCAATAAAAAACAAGCAGGGCGGGTGGTGGGGCACTGACTAGGAGGGCTGATTTGTAAGTTGGTAAGACTGTAGCTCTTTTTCCTAATTAGCTGAGGATGTGTTTAGGTTCCATTCAAAAAGTGGGCAT  
TCCTGGCCAGGCATGGTGGCTCACACCTGTAATCTCAGAGCTTTGGGAGACTGAGGTAGGAGGATCACTTGAGCCCAGGAATTTGAGATGAGCCTAGGCAACATAGTGAGACTCTTATCTCTATCAAAAAATAAAAATAAAAATGAGCCAGGCATGGTGCGGTGGCACGCACCTACTGCTAGGGGGGCTGAGGTGGGAGGATCACTTGAGCCTGGGAGGTTGAGGCTGCAGTGATCCCTGATCACAACATTGCATTTCAGCCTGGGTGACAGAGTGAGACCCTGTCTCAGAAAAAAAAAAAAAAAAGTCATTCCTGAAACCTCAGAATAGACCTACCTTGCCAAGGGCTTCCTTATGGGTAAGGACCTTATGGACCTGCTGGGACCCAAACTAGGCCTCACCTGATACGACCTGTCCTTCTCAAAACACCTAAACTTGGGAGAACATTGTCCCCCAGTGCTGGGGTAGGAGAGTCTGCCTGTTATTCTGCCTCTATGCAGAGAAGGAGCCCCAGATCAGCTTTTCCATGACAGGACAGTTTCCAAGATGCCACCTGTACTTGGAAGAAGCCAGGTTAAAATACTTTTCAAGTAAAACTTTCTTGATATTACTCTATCTTTCCCCAGGAGGACTGCATTACAACAAATTCGGACACCTGTGGCCTCTCCCTTCTATGCAAAGCAAAAAGCCAGCAGCAGCCCCAAGCTGATAAGATTAATCTAAAGAGCAAATTATGGTGTAATTTCCTATGCTGAAACTTTGTAGTTAATTTTTTAAAAAGGTTTCATTTTCCTATTGGTCTGATTTCACAGGAACATTTTACCTGTTTGTGAGGCATTTTTTCTCCTGGAAGAGAGGTGCTGATTGGCCCCAAGTGACTGACAATCTGGTGTAACGAAAATTTCCAATGTAAACTCATTTTCCCTCGGTTTCAGCAATTTTAAATCTATATATAGAGATATCTTTGTCAGCATTGCA
  
  
-- Factors predicted by PROMO in this sequence ----------------------
  
NAME; MATRIX\_WIDTH;
  
TFII-I [T00824]; 6
  
GR-beta [T01920]; 5
  
Ik-1 [T02702]; 13
  
HNF-4alpha [T03828]; 13
  
C/EBPbeta [T00581]; 4
  
LEF-1 [T02905]; 8
  
NF-1 [T00539]; 8
  
GR-alpha [T00337]; 5
  
AP-2alphaA [T00035]; 6
  
FOXP3 [T04280]; 6
  
PR B [T00696]; 7
  
PR A [T01661]; 7
  
RXR-alpha [T01345]; 7
  
RAR-beta [T00721]; 10
  
YY1 [T00915]; 4
  
TFIID [T00820]; 7
  
MEF-2A [T01005]; 11
  
HNF-3alpha [T02512]; 8
  
ENKTF-1 [T00255]; 8
  
XBP-1 [T00902]; 6
  
RAR-beta:RXR-alpha [T05420]; 12
  
Pax-5 [T00070]; 7
  
p53 [T00671]; 7
  
T3R-beta1 [T00851]; 9
  
GR [T05076]; 7
  
STAT4 [T01577]; 6
  
c-Ets-1 [T00112]; 7
  
Elk-1 [T00250]; 9
  
PPAR-alpha:RXR-alpha [T05221]; 11
  
AhR [T01795]; 11
  
AhR:Arnt [T05394]; 10
  
c-Jun [T00133]; 7
  
COUP-TF1 [T00149]; 13
  
PEA3 [T00685]; 9
  
HNF-1C [T01951]; 9
  
HOXD9 [T01424]; 10
  
HOXD10 [T01425]; 10
  
VDR [T00885]; 9
  
PXR-1:RXR-alpha [T05671]; 8
  
c-Myb [T00137]; 8
  
IRF-1 [T00423]; 9
  
NF-AT1 [T00550]; 9
  
NFI/CTF [T00094]; 8
  
EBF [T05427]; 11
  
USF2 [T00878]; 10
  
C/EBPalpha [T00105]; 7
  
AR [T00040]; 9
  
NF-AT2 [T01945]; 10
  
STAT1beta [T01573]; 10
  
c-Ets-2 [T00113]; 9
  
GATA-1 [T00306]; 6
  
GATA-2 [T00308]; 9
  
AP-1 [T00029]; 9
  
NF-AT1 [T01948]; 10
  
RBP-Jkappa [T01616]; 12
  
SRY [T00997]; 9
  
TCF-4E [T02878]; 7
  
NF-Y [T00150]; 8
  
CTF [T00174]; 12
  
IRF-2 [T01491]; 6
  
RelA [T00594]; 11
  
HNF-1A [T00368]; 8
  
HNF-1B [T01950]; 9
  
POU2F1 [T00641]; 11
  
POU2F2 (Oct-2.1) [T00646]; 11
  
GATA-3 [T00311]; 12
  
ETF [T00270]; 11
  
E2F-1 [T01542]; 8
  
PU.1 [T02068]; 13
  
Sp1 [T00759]; 10
  
NF-kappaB [T00590]; 12
  
ER-alpha [T00261]; 5
  
NF-kappaB1 [T00593]; 11
  
MAZ [T00490]; 13
  
WT1 [T00899]; 9
  
Egr-3 [T00243]; 13
  
  
-- PROMO predictions detail ------------------------------------------
  
  
Sequence name; Factor name; Start position; End position; Dissimilarity; String; RE equally; RE query
  
Sequence; TFII-I [T00824]; 20; 25; 14.269340; TAATCC; 5.86230; 5.77926;
  
Sequence; TFII-I [T00824]; 69; 74; 11.337888; GGAGTT; 2.44263; 2.50428;
  
Sequence; TFII-I [T00824]; 167; 172; 14.269340; TAATCC; 5.86230; 5.77926;
  
Sequence; TFII-I [T00824]; 196; 201; 11.337888; GGAGAA; 2.44263; 2.50428;
  
Sequence; TFII-I [T00824]; 215; 220; 6.581441; GGAGAT; 0.97705; 0.99908;
  
Sequence; TFII-I [T00824]; 251; 256; 6.581441; CACTCC; 0.97705; 0.99908;
  
Sequence; TFII-I [T00824]; 305; 310; 14.269340; GGAAGT; 5.86230; 5.77926;
  
Sequence; TFII-I [T00824]; 402; 407; 14.269340; TAATCC; 5.86230; 5.77926;
  
Sequence; TFII-I [T00824]; 448; 453; 6.581441; GGAGAT; 0.97705; 0.99908;
  
Sequence; TFII-I [T00824]; 454; 459; 11.337888; GGAGAC; 2.44263; 2.50428;
  
Sequence; TFII-I [T00824]; 459; 464; 9.512894; CCATCC; 7.32788; 7.45030;
  
Sequence; TFII-I [T00824]; 536; 541; 14.269340; TAGTCC; 5.86230; 5.77926;
  
Sequence; TFII-I [T00824]; 566; 571; 11.337888; GGAGAA; 2.44263; 2.50428;
  
Sequence; TFII-I [T00824]; 623; 628; 6.581441; CACTCC; 0.97705; 0.99908;
  
Sequence; TFII-I [T00824]; 648; 653; 14.269340; GATTCC; 5.86230; 5.77926;
  
Sequence; TFII-I [T00824]; 690; 695; 9.512894; GGAAAA; 7.32788; 7.45030;
  
Sequence; TFII-I [T00824]; 713; 718; 9.512894; GGAAGG; 7.32788; 7.45030;
  
Sequence; TFII-I [T00824]; 718; 723; 9.512894; GGAAGG; 7.32788; 7.45030;
  
Sequence; TFII-I [T00824]; 729; 734; 11.337888; GGAGAA; 2.44263; 2.50428;
  
Sequence; TFII-I [T00824]; 759; 764; 14.269340; GAATCC; 5.86230; 5.77926;
  
Sequence; TFII-I [T00824]; 880; 885; 9.512894; GTGTCC; 7.32788; 7.45030;
  
Sequence; TFII-I [T00824]; 935; 940; 11.337888; GGAGGG; 2.44263; 2.50428;
  
Sequence; TFII-I [T00824]; 972; 977; 9.512894; TTTTCC; 7.32788; 7.45030;
  
Sequence; TFII-I [T00824]; 989; 994; 14.269340; GGATGT; 5.86230; 5.77926;
  
Sequence; TFII-I [T00824]; 1020; 1025; 4.756447; CATTCC; 2.93115; 2.98746;
  
Sequence; TFII-I [T00824]; 1068; 1073; 11.337888; GGAGAC; 2.44263; 2.50428;
  
Sequence; TFII-I [T00824]; 1100; 1105; 9.512894; GGAATT; 7.32788; 7.45030;
  
Sequence; TFII-I [T00824]; 1331; 1336; 4.756447; CATTCC; 2.93115; 2.98746;
  
Sequence; TFII-I [T00824]; 1385; 1390; 14.269340; GGACCT; 5.86230; 5.77926;
  
Sequence; TFII-I [T00824]; 1394; 1399; 14.269340; GGACCT; 5.86230; 5.77926;
  
Sequence; TFII-I [T00824]; 1434; 1439; 0.000000; CTGTCC; 1.46558; 1.57268;
  
Sequence; TFII-I [T00824]; 1461; 1466; 11.337888; GGAGAA; 2.44263; 2.50428;
  
Sequence; TFII-I [T00824]; 1469; 1474; 9.512894; TTGTCC; 7.32788; 7.45030;
  
Sequence; TFII-I [T00824]; 1490; 1495; 1.824994; GGAGAG; 0.48853; 0.53907;
  
Sequence; TFII-I [T00824]; 1543; 1548; 9.512894; TTTTCC; 7.32788; 7.45030;
  
Sequence; TFII-I [T00824]; 1555; 1560; 0.000000; GGACAG; 1.46558; 1.57268;
  
Sequence; TFII-I [T00824]; 1560; 1565; 9.512894; GTTTCC; 7.32788; 7.45030;
  
Sequence; TFII-I [T00824]; 1639; 1644; 0.000000; CTTTCC; 1.46558; 1.57268;
  
Sequence; TFII-I [T00824]; 1651; 1656; 4.756447; GGACTG; 2.93115; 2.98746;
  
Sequence; TFII-I [T00824]; 1672; 1677; 9.512894; GGACAC; 7.32788; 7.45030;
  
Sequence; TFII-I [T00824]; 1685; 1690; 1.824994; CTCTCC; 0.48853; 0.53907;
  
Sequence; TFII-I [T00824]; 1764; 1769; 4.756447; ATTTCC; 2.93115; 2.98746;
  
Sequence; TFII-I [T00824]; 1810; 1815; 9.512894; TTTTCC; 7.32788; 7.45030;
  
Sequence; TFII-I [T00824]; 1864; 1869; 11.337888; TTCTCC; 2.44263; 2.50428;
  
Sequence; TFII-I [T00824]; 1923; 1928; 4.756447; ATTTCC; 2.93115; 2.98746;
  
Sequence; TFII-I [T00824]; 1941; 1946; 9.512894; TTTTCC; 7.32788; 7.45030;
  
Sequence; GR-beta [T01920]; 21; 25; 5.042296; AATCC; 3.90820; 4.04241;
  
Sequence; GR-beta [T01920]; 55; 59; 0.840383; TCATT; 7.81641; 8.51783;
  
Sequence; GR-beta [T01920]; 130; 134; 5.042296; AATAC; 3.90820; 4.04241;
  
Sequence; GR-beta [T01920]; 137; 141; 0.000000; AAATT; 3.90820; 4.28673;
  
Sequence; GR-beta [T01920]; 138; 142; 0.840383; AATTA; 7.81641; 8.51783;
  
Sequence; GR-beta [T01920]; 168; 172; 5.042296; AATCC; 3.90820; 4.04241;
  
Sequence; GR-beta [T01920]; 200; 204; 1.680765; AATGC; 3.90820; 4.04232;
  
Sequence; GR-beta [T01920]; 338; 342; 0.000000; ACATT; 3.90820; 4.28673;
  
Sequence; GR-beta [T01920]; 360; 364; 4.201913; AATAA; 7.81641; 8.53422;
  
Sequence; GR-beta [T01920]; 363; 367; 0.000000; AAATT; 3.90820; 4.28673;
  
Sequence; GR-beta [T01920]; 364; 368; 1.680765; AATTC; 3.90820; 4.04232;
  
Sequence; GR-beta [T01920]; 403; 407; 5.042296; AATCC; 3.90820; 4.04241;
  
Sequence; GR-beta [T01920]; 498; 502; 5.042296; AATAC; 3.90820; 4.04241;
  
Sequence; GR-beta [T01920]; 506; 510; 0.000000; AAATT; 3.90820; 4.28673;
  
Sequence; GR-beta [T01920]; 507; 511; 0.840383; AATTA; 7.81641; 8.51783;
  
Sequence; GR-beta [T01920]; 647; 651; 3.361531; AGATT; 3.90820; 4.28673;
  
Sequence; GR-beta [T01920]; 680; 684; 0.000000; AAATT; 3.90820; 4.28673;
  
Sequence; GR-beta [T01920]; 681; 685; 1.680765; AATTC; 3.90820; 4.04232;
  
Sequence; GR-beta [T01920]; 760; 764; 5.042296; AATCC; 3.90820; 4.04241;
  
Sequence; GR-beta [T01920]; 865; 869; 0.840383; AATGA; 7.81641; 8.51783;
  
Sequence; GR-beta [T01920]; 875; 879; 4.201913; TTATT; 7.81641; 8.53422;
  
Sequence; GR-beta [T01920]; 890; 894; 1.680765; GAATT; 3.90820; 4.04232;
  
Sequence; GR-beta [T01920]; 891; 895; 1.680765; AATTC; 3.90820; 4.04232;
  
Sequence; GR-beta [T01920]; 896; 900; 4.201913; AATAA; 7.81641; 8.53422;
  
Sequence; GR-beta [T01920]; 942; 946; 4.201913; TGATT; 7.81641; 8.53422;
  
Sequence; GR-beta [T01920]; 978; 982; 0.840383; TAATT; 7.81641; 8.51783;
  
Sequence; GR-beta [T01920]; 979; 983; 0.840383; AATTA; 7.81641; 8.51783;
  
Sequence; GR-beta [T01920]; 1004; 1008; 0.840383; CCATT; 7.81641; 8.51783;
  
Sequence; GR-beta [T01920]; 1019; 1023; 1.680765; GCATT; 3.90820; 4.04232;
  
Sequence; GR-beta [T01920]; 1053; 1057; 3.361531; AATCT; 3.90820; 4.28673;
  
Sequence; GR-beta [T01920]; 1101; 1105; 1.680765; GAATT; 3.90820; 4.04232;
  
Sequence; GR-beta [T01920]; 1102; 1106; 0.000000; AATTT; 3.90820; 4.28673;
  
Sequence; GR-beta [T01920]; 1152; 1156; 4.201913; AATAA; 7.81641; 8.53422;
  
Sequence; GR-beta [T01920]; 1158; 1162; 4.201913; AATAA; 7.81641; 8.53422;
  
Sequence; GR-beta [T01920]; 1164; 1168; 0.840383; AATGA; 7.81641; 8.51783;
  
Sequence; GR-beta [T01920]; 1269; 1273; 0.000000; ACATT; 3.90820; 4.28673;
  
Sequence; GR-beta [T01920]; 1274; 1278; 1.680765; GCATT; 3.90820; 4.04232;
  
Sequence; GR-beta [T01920]; 1330; 1334; 0.840383; TCATT; 7.81641; 8.51783;
  
Sequence; GR-beta [T01920]; 1348; 1352; 4.201913; AATAG; 7.81641; 8.53422;
  
Sequence; GR-beta [T01920]; 1466; 1470; 0.000000; ACATT; 3.90820; 4.28673;
  
Sequence; GR-beta [T01920]; 1504; 1508; 4.201913; TTATT; 7.81641; 8.53422;
  
Sequence; GR-beta [T01920]; 1601; 1605; 5.042296; AATAC; 3.90820; 4.04241;
  
Sequence; GR-beta [T01920]; 1627; 1631; 3.361531; ATATT; 3.90820; 4.28673;
  
Sequence; GR-beta [T01920]; 1656; 1660; 1.680765; GCATT; 3.90820; 4.04232;
  
Sequence; GR-beta [T01920]; 1666; 1670; 0.000000; AAATT; 3.90820; 4.28673;
  
Sequence; GR-beta [T01920]; 1667; 1671; 1.680765; AATTC; 3.90820; 4.04232;
  
Sequence; GR-beta [T01920]; 1734; 1738; 3.361531; AGATT; 3.90820; 4.28673;
  
Sequence; GR-beta [T01920]; 1739; 1743; 3.361531; AATCT; 3.90820; 4.28673;
  
Sequence; GR-beta [T01920]; 1751; 1755; 0.000000; AAATT; 3.90820; 4.28673;
  
Sequence; GR-beta [T01920]; 1752; 1756; 0.840383; AATTA; 7.81641; 8.51783;
  
Sequence; GR-beta [T01920]; 1762; 1766; 0.840383; TAATT; 7.81641; 8.51783;
  
Sequence; GR-beta [T01920]; 1763; 1767; 0.000000; AATTT; 3.90820; 4.28673;
  
Sequence; GR-beta [T01920]; 1789; 1793; 0.840383; TAATT; 7.81641; 8.51783;
  
Sequence; GR-beta [T01920]; 1790; 1794; 0.000000; AATTT; 3.90820; 4.28673;
  
Sequence; GR-beta [T01920]; 1807; 1811; 0.840383; TCATT; 7.81641; 8.51783;
  
Sequence; GR-beta [T01920]; 1815; 1819; 4.201913; CTATT; 7.81641; 8.53422;
  
Sequence; GR-beta [T01920]; 1824; 1828; 4.201913; TGATT; 7.81641; 8.53422;
  
Sequence; GR-beta [T01920]; 1837; 1841; 0.000000; ACATT; 3.90820; 4.28673;
  
Sequence; GR-beta [T01920]; 1857; 1861; 1.680765; GCATT; 3.90820; 4.04232;
  
Sequence; GR-beta [T01920]; 1884; 1888; 4.201913; TGATT; 7.81641; 8.53422;
  
Sequence; GR-beta [T01920]; 1906; 1910; 3.361531; AATCT; 3.90820; 4.28673;
  
Sequence; GR-beta [T01920]; 1921; 1925; 0.000000; AAATT; 3.90820; 4.28673;
  
Sequence; GR-beta [T01920]; 1922; 1926; 0.000000; AATTT; 3.90820; 4.28673;
  
Sequence; GR-beta [T01920]; 1929; 1933; 0.000000; AATGT; 3.90820; 4.28673;
  
Sequence; GR-beta [T01920]; 1938; 1942; 0.840383; TCATT; 7.81641; 8.51783;
  
Sequence; GR-beta [T01920]; 1958; 1962; 0.840383; CAATT; 7.81641; 8.51783;
  
Sequence; GR-beta [T01920]; 1959; 1963; 0.000000; AATTT; 3.90820; 4.28673;
  
Sequence; GR-beta [T01920]; 1966; 1970; 3.361531; AATCT; 3.90820; 4.28673;
  
Sequence; GR-beta [T01920]; 1993; 1997; 1.680765; GCATT; 3.90820; 4.04232;
  
Sequence; Ik-1 [T02702]; 23; 35; 2.374299; TCCCAGCACTTTG; 0.00063; 0.00065;
  
Sequence; Ik-1 [T02702]; 170; 182; 4.748597; TCCCAGCTACTCG; 0.00313; 0.00321;
  
Sequence; Ik-1 [T02702]; 405; 417; 2.374299; TCCCAGCACTTTG; 0.00063; 0.00065;
  
Sequence; Ik-1 [T02702]; 539; 551; 4.748597; TCCCAGCTACTCG; 0.00313; 0.00321;
  
Sequence; Ik-1 [T02702]; 719; 731; 7.122895; GAAGGTTCTGGGA; 0.01064; 0.01087;
  
Sequence; Ik-1 [T02702]; 1208; 1220; 14.245790; GGCTGAGGTGGGA; 0.12004; 0.12124;
  
Sequence; Ik-1 [T02702]; 1394; 1406; 14.245790; GGACCTGCTGGGA; 0.12004; 0.12124;
  
Sequence; HNF-4alpha [T03828]; 23; 35; 12.669538; TCCCAGCACTTTG; 0.02755; 0.02884;
  
Sequence; HNF-4alpha [T03828]; 405; 417; 12.669538; TCCCAGCACTTTG; 0.02755; 0.02884;
  
Sequence; HNF-4alpha [T03828]; 1772; 1784; 11.074603; TGCTGAAACTTTG; 0.01628; 0.01713;
  
Sequence; C/EBPbeta [T00581]; 33; 36; 1.639871; TTGG; 15.63281; 15.70864;
  
Sequence; C/EBPbeta [T00581]; 60; 63; 1.366559; TTGA; 15.63281; 15.70864;
  
Sequence; C/EBPbeta [T00581]; 89; 92; 1.639871; CCAA; 15.63281; 15.70864;
  
Sequence; C/EBPbeta [T00581]; 206; 209; 1.366559; TTGA; 15.63281; 15.70864;
  
Sequence; C/EBPbeta [T00581]; 220; 223; 1.366559; TCAA; 15.63281; 15.70864;
  
Sequence; C/EBPbeta [T00581]; 320; 323; 0.000000; GCAA; 15.63281; 16.08924;
  
Sequence; C/EBPbeta [T00581]; 331; 334; 0.000000; GCAA; 15.63281; 16.08924;
  
Sequence; C/EBPbeta [T00581]; 415; 418; 1.639871; TTGG; 15.63281; 15.70864;
  
Sequence; C/EBPbeta [T00581]; 501; 504; 0.000000; ACAA; 15.63281; 16.08924;
  
Sequence; C/EBPbeta [T00581]; 596; 599; 0.000000; TTGC; 15.63281; 16.08924;
  
Sequence; C/EBPbeta [T00581]; 644; 647; 0.000000; GCAA; 15.63281; 16.08924;
  
Sequence; C/EBPbeta [T00581]; 657; 660; 1.366559; TCAA; 15.63281; 15.70864;
  
Sequence; C/EBPbeta [T00581]; 708; 711; 1.639871; CCAA; 15.63281; 15.70864;
  
Sequence; C/EBPbeta [T00581]; 755; 758; 1.639871; CCAA; 15.63281; 15.70864;
  
Sequence; C/EBPbeta [T00581]; 802; 805; 0.000000; TTGC; 15.63281; 16.08924;
  
Sequence; C/EBPbeta [T00581]; 863; 866; 0.000000; GCAA; 15.63281; 16.08924;
  
Sequence; C/EBPbeta [T00581]; 878; 881; 0.000000; TTGT; 15.63281; 16.08924;
  
Sequence; C/EBPbeta [T00581]; 894; 897; 1.366559; TCAA; 15.63281; 15.70864;
  
Sequence; C/EBPbeta [T00581]; 904; 907; 0.000000; ACAA; 15.63281; 16.08924;
  
Sequence; C/EBPbeta [T00581]; 946; 949; 0.000000; TTGT; 15.63281; 16.08924;
  
Sequence; C/EBPbeta [T00581]; 953; 956; 1.639871; TTGG; 15.63281; 15.70864;
  
Sequence; C/EBPbeta [T00581]; 1008; 1011; 1.366559; TCAA; 15.63281; 15.70864;
  
Sequence; C/EBPbeta [T00581]; 1065; 1068; 1.639871; TTGG; 15.63281; 15.70864;
  
Sequence; C/EBPbeta [T00581]; 1091; 1094; 1.366559; TTGA; 15.63281; 15.70864;
  
Sequence; C/EBPbeta [T00581]; 1105; 1108; 1.366559; TTGA; 15.63281; 15.70864;
  
Sequence; C/EBPbeta [T00581]; 1120; 1123; 0.000000; GCAA; 15.63281; 16.08924;
  
Sequence; C/EBPbeta [T00581]; 1146; 1149; 1.366559; TCAA; 15.63281; 15.70864;
  
Sequence; C/EBPbeta [T00581]; 1228; 1231; 1.366559; TTGA; 15.63281; 15.70864;
  
Sequence; C/EBPbeta [T00581]; 1242; 1245; 1.366559; TTGA; 15.63281; 15.70864;
  
Sequence; C/EBPbeta [T00581]; 1266; 1269; 0.000000; ACAA; 15.63281; 16.08924;
  
Sequence; C/EBPbeta [T00581]; 1272; 1275; 0.000000; TTGC; 15.63281; 16.08924;
  
Sequence; C/EBPbeta [T00581]; 1360; 1363; 0.000000; TTGC; 15.63281; 16.08924;
  
Sequence; C/EBPbeta [T00581]; 1363; 1366; 1.639871; CCAA; 15.63281; 15.70864;
  
Sequence; C/EBPbeta [T00581]; 1408; 1411; 1.639871; CCAA; 15.63281; 15.70864;
  
Sequence; C/EBPbeta [T00581]; 1443; 1446; 1.366559; TCAA; 15.63281; 15.70864;
  
Sequence; C/EBPbeta [T00581]; 1458; 1461; 1.639871; TTGG; 15.63281; 15.70864;
  
Sequence; C/EBPbeta [T00581]; 1469; 1472; 0.000000; TTGT; 15.63281; 16.08924;
  
Sequence; C/EBPbeta [T00581]; 1564; 1567; 1.639871; CCAA; 15.63281; 15.70864;
  
Sequence; C/EBPbeta [T00581]; 1582; 1585; 1.639871; TTGG; 15.63281; 15.70864;
  
Sequence; C/EBPbeta [T00581]; 1609; 1612; 1.366559; TCAA; 15.63281; 15.70864;
  
Sequence; C/EBPbeta [T00581]; 1624; 1627; 1.366559; TTGA; 15.63281; 15.70864;
  
Sequence; C/EBPbeta [T00581]; 1661; 1664; 0.000000; ACAA; 15.63281; 16.08924;
  
Sequence; C/EBPbeta [T00581]; 1664; 1667; 0.000000; ACAA; 15.63281; 16.08924;
  
Sequence; C/EBPbeta [T00581]; 1698; 1701; 0.000000; GCAA; 15.63281; 16.08924;
  
Sequence; C/EBPbeta [T00581]; 1703; 1706; 0.000000; GCAA; 15.63281; 16.08924;
  
Sequence; C/EBPbeta [T00581]; 1723; 1726; 1.639871; CCAA; 15.63281; 15.70864;
  
Sequence; C/EBPbeta [T00581]; 1749; 1752; 0.000000; GCAA; 15.63281; 16.08924;
  
Sequence; C/EBPbeta [T00581]; 1782; 1785; 0.000000; TTGT; 15.63281; 16.08924;
  
Sequence; C/EBPbeta [T00581]; 1818; 1821; 1.639871; TTGG; 15.63281; 15.70864;
  
Sequence; C/EBPbeta [T00581]; 1850; 1853; 0.000000; TTGT; 15.63281; 16.08924;
  
Sequence; C/EBPbeta [T00581]; 1887; 1890; 1.639871; TTGG; 15.63281; 15.70864;
  
Sequence; C/EBPbeta [T00581]; 1893; 1896; 1.639871; CCAA; 15.63281; 15.70864;
  
Sequence; C/EBPbeta [T00581]; 1904; 1907; 0.000000; ACAA; 15.63281; 16.08924;
  
Sequence; C/EBPbeta [T00581]; 1927; 1930; 1.639871; CCAA; 15.63281; 15.70864;
  
Sequence; C/EBPbeta [T00581]; 1957; 1960; 0.000000; GCAA; 15.63281; 16.08924;
  
Sequence; C/EBPbeta [T00581]; 1987; 1990; 0.000000; TTGT; 15.63281; 16.08924;
  
Sequence; C/EBPbeta [T00581]; 1996; 1999; 0.000000; TTGC; 15.63281; 16.08924;
  
Sequence; LEF-1 [T02905]; 31; 38; 8.973041; CTTTGGGA; 0.54959; 0.56713;
  
Sequence; LEF-1 [T02905]; 413; 420; 8.973041; CTTTGGGA; 0.54959; 0.56713;
  
Sequence; LEF-1 [T02905]; 1063; 1070; 8.973041; CTTTGGGA; 0.54959; 0.56713;
  
Sequence; LEF-1 [T02905]; 1696; 1703; 8.759086; ATGCAAAG; 0.54959; 0.56713;
  
Sequence; LEF-1 [T02905]; 1780; 1787; 9.099721; CTTTGTAG; 0.54959; 0.56713;
  
Sequence; LEF-1 [T02905]; 1985; 1992; 9.724040; CTTTGTCA; 0.21373; 0.22471;
  
Sequence; NF-1 [T00539]; 33; 40; 8.790071; TTGGGAGG; 0.24426; 0.24593;
  
Sequence; NF-1 [T00539]; 85; 92; 10.857758; CTAGCCAA; 0.61066; 0.59380;
  
Sequence; NF-1 [T00539]; 415; 422; 8.790071; TTGGGAGG; 0.24426; 0.24593;
  
Sequence; NF-1 [T00539]; 704; 711; 11.004208; GAGACCAA; 0.61066; 0.59380;
  
Sequence; NF-1 [T00539]; 751; 758; 2.813149; GGGCCCAA; 0.24426; 0.22703;
  
Sequence; NF-1 [T00539]; 953; 960; 11.603221; TTGGTAAG; 0.48853; 0.49603;
  
Sequence; NF-1 [T00539]; 1065; 1072; 8.790071; TTGGGAGA; 0.24426; 0.24593;
  
Sequence; NF-1 [T00539]; 1359; 1366; 8.790071; CTTGCCAA; 0.24426; 0.24593;
  
Sequence; NF-1 [T00539]; 1404; 1411; 6.948522; GGACCCAA; 0.48853; 0.46277;
  
Sequence; NF-1 [T00539]; 1458; 1465; 8.790071; TTGGGAGA; 0.24426; 0.24593;
  
Sequence; NF-1 [T00539]; 1560; 1567; 14.416371; GTTTCCAA; 0.24426; 0.24944;
  
Sequence; NF-1 [T00539]; 1582; 1589; 11.603221; TTGGAAGA; 0.48853; 0.49603;
  
Sequence; NF-1 [T00539]; 1719; 1726; 6.948522; AGCCCCAA; 0.48853; 0.46277;
  
Sequence; NF-1 [T00539]; 1818; 1825; 8.191058; TTGGTCTG; 0.24426; 0.24090;
  
Sequence; NF-1 [T00539]; 1887; 1894; 2.813149; TTGGCCCC; 0.24426; 0.22703;
  
Sequence; NF-1 [T00539]; 1889; 1896; 6.948522; GGCCCCAA; 0.48853; 0.46277;
  
Sequence; NF-1 [T00539]; 1923; 1930; 14.416371; ATTTCCAA; 0.24426; 0.24944;
  
Sequence; GR-alpha [T00337]; 16; 20; 0.000000; CCTAT; 7.81641; 8.34392;
  
Sequence; GR-alpha [T00337]; 36; 40; 8.281568; GGAGG; 7.81641; 7.86291;
  
Sequence; GR-alpha [T00337]; 42; 46; 6.263098; TGAGG; 3.90820; 3.98654;
  
Sequence; GR-alpha [T00337]; 61; 65; 6.263098; TGAGG; 3.90820; 3.98654;
  
Sequence; GR-alpha [T00337]; 66; 70; 8.073878; CCAGG; 7.81641; 7.35374;
  
Sequence; GR-alpha [T00337]; 84; 88; 8.073878; CCTAG; 7.81641; 7.35374;
  
Sequence; GR-alpha [T00337]; 144; 148; 8.073878; CCAGG; 7.81641; 7.35374;
  
Sequence; GR-alpha [T00337]; 163; 167; 0.000000; CCTGT; 7.81641; 8.34392;
  
Sequence; GR-alpha [T00337]; 189; 193; 6.263098; TGAGG; 3.90820; 3.98654;
  
Sequence; GR-alpha [T00337]; 204; 208; 8.281568; CCTTG; 7.81641; 7.86291;
  
Sequence; GR-alpha [T00337]; 212; 216; 8.073878; CCAGG; 7.81641; 7.35374;
  
Sequence; GR-alpha [T00337]; 221; 225; 8.281568; CAAGG; 7.81641; 7.86291;
  
Sequence; GR-alpha [T00337]; 259; 263; 8.073878; CCTGG; 7.81641; 7.35374;
  
Sequence; GR-alpha [T00337]; 280; 284; 8.073878; GCAGG; 7.81641; 7.35374;
  
Sequence; GR-alpha [T00337]; 291; 295; 6.263098; TGAGG; 3.90820; 3.98654;
  
Sequence; GR-alpha [T00337]; 316; 320; 0.207689; AGAGG; 7.81641; 8.34392;
  
Sequence; GR-alpha [T00337]; 326; 330; 6.263098; CCTTA; 3.90820; 3.98654;
  
Sequence; GR-alpha [T00337]; 369; 373; 0.000000; ATAGG; 7.81641; 8.34392;
  
Sequence; GR-alpha [T00337]; 373; 377; 8.281568; GGAGG; 7.81641; 7.86291;
  
Sequence; GR-alpha [T00337]; 379; 383; 8.073878; CCAGG; 7.81641; 7.35374;
  
Sequence; GR-alpha [T00337]; 398; 402; 0.000000; CCTGT; 7.81641; 8.34392;
  
Sequence; GR-alpha [T00337]; 418; 422; 8.281568; GGAGG; 7.81641; 7.86291;
  
Sequence; GR-alpha [T00337]; 424; 428; 8.281568; CGAGG; 7.81641; 7.86291;
  
Sequence; GR-alpha [T00337]; 428; 432; 8.073878; GCAGG; 7.81641; 7.35374;
  
Sequence; GR-alpha [T00337]; 440; 444; 8.281568; CGAGG; 7.81641; 7.86291;
  
Sequence; GR-alpha [T00337]; 445; 449; 6.055408; TCAGG; 3.90820; 3.81078;
  
Sequence; GR-alpha [T00337]; 463; 467; 8.073878; CCTGG; 7.81641; 7.35374;
  
Sequence; GR-alpha [T00337]; 525; 529; 8.073878; GCAGG; 7.81641; 7.35374;
  
Sequence; GR-alpha [T00337]; 532; 536; 0.000000; CCTGT; 7.81641; 8.34392;
  
Sequence; GR-alpha [T00337]; 553; 557; 8.281568; GGAGG; 7.81641; 7.86291;
  
Sequence; GR-alpha [T00337]; 559; 563; 6.263098; TGAGG; 3.90820; 3.98654;
  
Sequence; GR-alpha [T00337]; 563; 567; 8.073878; GCAGG; 7.81641; 7.35374;
  
Sequence; GR-alpha [T00337]; 582; 586; 8.073878; CCAGG; 7.81641; 7.35374;
  
Sequence; GR-alpha [T00337]; 585; 589; 8.281568; GGAGG; 7.81641; 7.86291;
  
Sequence; GR-alpha [T00337]; 631; 635; 8.073878; CCTGG; 7.81641; 7.35374;
  
Sequence; GR-alpha [T00337]; 686; 690; 0.000000; ATAGG; 7.81641; 8.34392;
  
Sequence; GR-alpha [T00337]; 696; 700; 8.281568; GAAGG; 7.81641; 7.86291;
  
Sequence; GR-alpha [T00337]; 709; 713; 8.281568; CAAGG; 7.81641; 7.86291;
  
Sequence; GR-alpha [T00337]; 714; 718; 8.281568; GAAGG; 7.81641; 7.86291;
  
Sequence; GR-alpha [T00337]; 719; 723; 8.281568; GAAGG; 7.81641; 7.86291;
  
Sequence; GR-alpha [T00337]; 737; 741; 8.073878; GCAGG; 7.81641; 7.35374;
  
Sequence; GR-alpha [T00337]; 744; 748; 8.073878; GCAGG; 7.81641; 7.35374;
  
Sequence; GR-alpha [T00337]; 748; 752; 8.073878; GCAGG; 7.81641; 7.35374;
  
Sequence; GR-alpha [T00337]; 763; 767; 8.073878; CCTGC; 7.81641; 7.35374;
  
Sequence; GR-alpha [T00337]; 779; 783; 6.263098; CCTTA; 3.90820; 3.98654;
  
Sequence; GR-alpha [T00337]; 787; 791; 8.281568; GGAGG; 7.81641; 7.86291;
  
Sequence; GR-alpha [T00337]; 800; 804; 8.281568; CCTTG; 7.81641; 7.86291;
  
Sequence; GR-alpha [T00337]; 806; 810; 0.000000; ACAGG; 7.81641; 8.34392;
  
Sequence; GR-alpha [T00337]; 813; 817; 8.073878; CCAGG; 7.81641; 7.35374;
  
Sequence; GR-alpha [T00337]; 820; 824; 6.055408; CCTGA; 3.90820; 3.81078;
  
Sequence; GR-alpha [T00337]; 839; 843; 6.263098; CCTTA; 3.90820; 3.98654;
  
Sequence; GR-alpha [T00337]; 859; 863; 6.263098; TGAGG; 3.90820; 3.98654;
  
Sequence; GR-alpha [T00337]; 873; 877; 6.263098; CCTTA; 3.90820; 3.98654;
  
Sequence; GR-alpha [T00337]; 908; 912; 8.073878; GCAGG; 7.81641; 7.35374;
  
Sequence; GR-alpha [T00337]; 932; 936; 8.073878; CTAGG; 7.81641; 7.35374;
  
Sequence; GR-alpha [T00337]; 935; 939; 8.281568; GGAGG; 7.81641; 7.86291;
  
Sequence; GR-alpha [T00337]; 976; 980; 6.055408; CCTAA; 3.90820; 3.81078;
  
Sequence; GR-alpha [T00337]; 986; 990; 6.263098; TGAGG; 3.90820; 3.98654;
  
Sequence; GR-alpha [T00337]; 997; 1001; 6.055408; TTAGG; 3.90820; 3.81078;
  
Sequence; GR-alpha [T00337]; 1024; 1028; 8.073878; CCTGG; 7.81641; 7.35374;
  
Sequence; GR-alpha [T00337]; 1029; 1033; 8.073878; CCAGG; 7.81641; 7.35374;
  
Sequence; GR-alpha [T00337]; 1048; 1052; 0.000000; CCTGT; 7.81641; 8.34392;
  
Sequence; GR-alpha [T00337]; 1074; 1078; 6.263098; TGAGG; 3.90820; 3.98654;
  
Sequence; GR-alpha [T00337]; 1078; 1082; 8.073878; GTAGG; 7.81641; 7.35374;
  
Sequence; GR-alpha [T00337]; 1081; 1085; 8.281568; GGAGG; 7.81641; 7.86291;
  
Sequence; GR-alpha [T00337]; 1097; 1101; 8.073878; CCAGG; 7.81641; 7.35374;
  
Sequence; GR-alpha [T00337]; 1115; 1119; 8.073878; CCTAG; 7.81641; 7.35374;
  
Sequence; GR-alpha [T00337]; 1116; 1120; 8.073878; CTAGG; 7.81641; 7.35374;
  
Sequence; GR-alpha [T00337]; 1170; 1174; 8.073878; CCAGG; 7.81641; 7.35374;
  
Sequence; GR-alpha [T00337]; 1194; 1198; 8.073878; CCTAC; 7.81641; 7.35374;
  
Sequence; GR-alpha [T00337]; 1201; 1205; 8.073878; CTAGG; 7.81641; 7.35374;
  
Sequence; GR-alpha [T00337]; 1211; 1215; 6.263098; TGAGG; 3.90820; 3.98654;
  
Sequence; GR-alpha [T00337]; 1218; 1222; 8.281568; GGAGG; 7.81641; 7.86291;
  
Sequence; GR-alpha [T00337]; 1233; 1237; 8.073878; CCTGG; 7.81641; 7.35374;
  
Sequence; GR-alpha [T00337]; 1237; 1241; 8.281568; GGAGG; 7.81641; 7.86291;
  
Sequence; GR-alpha [T00337]; 1243; 1247; 6.263098; TGAGG; 3.90820; 3.98654;
  
Sequence; GR-alpha [T00337]; 1259; 1263; 6.055408; CCTGA; 3.90820; 3.81078;
  
Sequence; GR-alpha [T00337]; 1283; 1287; 8.073878; CCTGG; 7.81641; 7.35374;
  
Sequence; GR-alpha [T00337]; 1303; 1307; 0.000000; CCTGT; 7.81641; 8.34392;
  
Sequence; GR-alpha [T00337]; 1335; 1339; 6.055408; CCTGA; 3.90820; 3.81078;
  
Sequence; GR-alpha [T00337]; 1342; 1346; 6.263098; CCTCA; 3.90820; 3.98654;
  
Sequence; GR-alpha [T00337]; 1354; 1358; 8.073878; CCTAC; 7.81641; 7.35374;
  
Sequence; GR-alpha [T00337]; 1358; 1362; 8.281568; CCTTG; 7.81641; 7.86291;
  
Sequence; GR-alpha [T00337]; 1364; 1368; 8.281568; CAAGG; 7.81641; 7.86291;
  
Sequence; GR-alpha [T00337]; 1373; 1377; 6.263098; CCTTA; 3.90820; 3.98654;
  
Sequence; GR-alpha [T00337]; 1382; 1386; 6.263098; TAAGG; 3.90820; 3.98654;
  
Sequence; GR-alpha [T00337]; 1388; 1392; 6.263098; CCTTA; 3.90820; 3.98654;
  
Sequence; GR-alpha [T00337]; 1397; 1401; 8.073878; CCTGC; 7.81641; 7.35374;
  
Sequence; GR-alpha [T00337]; 1413; 1417; 8.073878; CTAGG; 7.81641; 7.35374;
  
Sequence; GR-alpha [T00337]; 1418; 1422; 6.263098; CCTCA; 3.90820; 3.98654;
  
Sequence; GR-alpha [T00337]; 1423; 1427; 6.055408; CCTGA; 3.90820; 3.81078;
  
Sequence; GR-alpha [T00337]; 1433; 1437; 0.000000; CCTGT; 7.81641; 8.34392;
  
Sequence; GR-alpha [T00337]; 1438; 1442; 8.281568; CCTTC; 7.81641; 7.86291;
  
Sequence; GR-alpha [T00337]; 1451; 1455; 6.055408; CCTAA; 3.90820; 3.81078;
  
Sequence; GR-alpha [T00337]; 1487; 1491; 8.073878; GTAGG; 7.81641; 7.35374;
  
Sequence; GR-alpha [T00337]; 1500; 1504; 0.000000; CCTGT; 7.81641; 8.34392;
  
Sequence; GR-alpha [T00337]; 1512; 1516; 0.207689; CCTCT; 7.81641; 8.34392;
  
Sequence; GR-alpha [T00337]; 1524; 1528; 8.281568; GAAGG; 7.81641; 7.86291;
  
Sequence; GR-alpha [T00337]; 1552; 1556; 0.000000; ACAGG; 7.81641; 8.34392;
  
Sequence; GR-alpha [T00337]; 1575; 1579; 0.000000; CCTGT; 7.81641; 8.34392;
  
Sequence; GR-alpha [T00337]; 1592; 1596; 8.073878; CCAGG; 7.81641; 7.35374;
  
Sequence; GR-alpha [T00337]; 1645; 1649; 8.073878; CCAGG; 7.81641; 7.35374;
  
Sequence; GR-alpha [T00337]; 1648; 1652; 8.281568; GGAGG; 7.81641; 7.86291;
  
Sequence; GR-alpha [T00337]; 1677; 1681; 0.000000; CCTGT; 7.81641; 8.34392;
  
Sequence; GR-alpha [T00337]; 1684; 1688; 0.207689; CCTCT; 7.81641; 8.34392;
  
Sequence; GR-alpha [T00337]; 1690; 1694; 8.281568; CCTTC; 7.81641; 7.86291;
  
Sequence; GR-alpha [T00337]; 1768; 1772; 0.000000; CCTAT; 7.81641; 8.34392;
  
Sequence; GR-alpha [T00337]; 1800; 1804; 0.207689; AAAGG; 7.81641; 8.34392;
  
Sequence; GR-alpha [T00337]; 1814; 1818; 0.000000; CCTAT; 7.81641; 8.34392;
  
Sequence; GR-alpha [T00337]; 1831; 1835; 0.000000; ACAGG; 7.81641; 8.34392;
  
Sequence; GR-alpha [T00337]; 1845; 1849; 0.000000; CCTGT; 7.81641; 8.34392;
  
Sequence; GR-alpha [T00337]; 1853; 1857; 6.263098; TGAGG; 3.90820; 3.98654;
  
Sequence; GR-alpha [T00337]; 1868; 1872; 8.073878; CCTGG; 7.81641; 7.35374;
  
Sequence; GR-alpha [T00337]; 1876; 1880; 0.207689; AGAGG; 7.81641; 8.34392;
  
Sequence; GR-alpha [T00337]; 1946; 1950; 8.281568; CCTCG; 7.81641; 7.86291;
  
Sequence; AP-2alphaA [T00035]; 15; 20; 5.568965; GCCTAT; 0.48853; 0.48156;
  
Sequence; AP-2alphaA [T00035]; 36; 41; 1.871933; GGAGGC; 0.97705; 0.88570;
  
Sequence; AP-2alphaA [T00035]; 61; 66; 2.550491; TGAGGC; 0.48853; 0.45385;
  
Sequence; AP-2alphaA [T00035]; 83; 88; 4.438035; GCCTAG; 0.97705; 0.92532;
  
Sequence; AP-2alphaA [T00035]; 144; 149; 0.226186; CCAGGC; 0.97705; 0.84668;
  
Sequence; AP-2alphaA [T00035]; 162; 167; 1.357116; GCCTGT; 0.48853; 0.45437;
  
Sequence; AP-2alphaA [T00035]; 189; 194; 2.550491; TGAGGC; 0.48853; 0.45385;
  
Sequence; AP-2alphaA [T00035]; 203; 208; 3.970052; GCCTTG; 0.97705; 0.90823;
  
Sequence; AP-2alphaA [T00035]; 221; 226; 3.970052; CAAGGC; 0.97705; 0.90823;
  
Sequence; AP-2alphaA [T00035]; 258; 263; 0.226186; GCCTGG; 0.97705; 0.84668;
  
Sequence; AP-2alphaA [T00035]; 316; 321; 3.229049; AGAGGC; 0.48853; 0.48567;
  
Sequence; AP-2alphaA [T00035]; 373; 378; 1.871933; GGAGGC; 0.97705; 0.88570;
  
Sequence; AP-2alphaA [T00035]; 379; 384; 0.226186; CCAGGC; 0.97705; 0.84668;
  
Sequence; AP-2alphaA [T00035]; 397; 402; 1.357116; GCCTGT; 0.48853; 0.45437;
  
Sequence; AP-2alphaA [T00035]; 418; 423; 1.871933; GGAGGC; 0.97705; 0.88570;
  
Sequence; AP-2alphaA [T00035]; 424; 429; 2.098119; CGAGGC; 0.97705; 0.88570;
  
Sequence; AP-2alphaA [T00035]; 428; 433; 0.000000; GCAGGC; 0.97705; 0.84668;
  
Sequence; AP-2alphaA [T00035]; 531; 536; 1.357116; GCCTGT; 0.48853; 0.45437;
  
Sequence; AP-2alphaA [T00035]; 553; 558; 1.871933; GGAGGC; 0.97705; 0.88570;
  
Sequence; AP-2alphaA [T00035]; 559; 564; 2.550491; TGAGGC; 0.48853; 0.45385;
  
Sequence; AP-2alphaA [T00035]; 585; 590; 1.871933; GGAGGC; 0.97705; 0.88570;
  
Sequence; AP-2alphaA [T00035]; 630; 635; 0.226186; GCCTGG; 0.97705; 0.84668;
  
Sequence; AP-2alphaA [T00035]; 744; 749; 0.000000; GCAGGC; 0.97705; 0.84668;
  
Sequence; AP-2alphaA [T00035]; 799; 804; 3.970052; GCCTTG; 0.97705; 0.90823;
  
Sequence; AP-2alphaA [T00035]; 813; 818; 0.226186; CCAGGC; 0.97705; 0.84668;
  
Sequence; AP-2alphaA [T00035]; 859; 864; 2.550491; TGAGGC; 0.48853; 0.45385;
  
Sequence; AP-2alphaA [T00035]; 1029; 1034; 0.226186; CCAGGC; 0.97705; 0.84668;
  
Sequence; AP-2alphaA [T00035]; 1114; 1119; 4.438035; GCCTAG; 0.97705; 0.92532;
  
Sequence; AP-2alphaA [T00035]; 1116; 1121; 4.438035; CTAGGC; 0.97705; 0.92532;
  
Sequence; AP-2alphaA [T00035]; 1170; 1175; 0.226186; CCAGGC; 0.97705; 0.84668;
  
Sequence; AP-2alphaA [T00035]; 1232; 1237; 0.226186; GCCTGG; 0.97705; 0.84668;
  
Sequence; AP-2alphaA [T00035]; 1243; 1248; 2.550491; TGAGGC; 0.48853; 0.45385;
  
Sequence; AP-2alphaA [T00035]; 1282; 1287; 0.226186; GCCTGG; 0.97705; 0.84668;
  
Sequence; AP-2alphaA [T00035]; 1413; 1418; 4.438035; CTAGGC; 0.97705; 0.92532;
  
Sequence; AP-2alphaA [T00035]; 1417; 1422; 2.550491; GCCTCA; 0.48853; 0.45385;
  
Sequence; AP-2alphaA [T00035]; 1499; 1504; 1.357116; GCCTGT; 0.48853; 0.45437;
  
Sequence; AP-2alphaA [T00035]; 1511; 1516; 3.229049; GCCTCT; 0.48853; 0.48567;
  
Sequence; AP-2alphaA [T00035]; 1683; 1688; 3.229049; GCCTCT; 0.48853; 0.48567;
  
Sequence; AP-2alphaA [T00035]; 1853; 1858; 2.550491; TGAGGC; 0.48853; 0.45385;
  
Sequence; FOXP3 [T04280]; 9; 14; 14.269340; GTTCAC; 5.86230; 6.01111;
  
Sequence; FOXP3 [T04280]; 88; 93; 9.512894; GCCAAC; 7.32788; 7.39742;
  
Sequence; FOXP3 [T04280]; 321; 326; 4.756447; CAAAAC; 2.93115; 3.09069;
  
Sequence; FOXP3 [T04280]; 330; 335; 9.512894; AGCAAC; 7.32788; 7.39742;
  
Sequence; FOXP3 [T04280]; 567; 572; 9.512894; GAGAAC; 7.32788; 7.39742;
  
Sequence; FOXP3 [T04280]; 576; 581; 14.269340; ATGAAC; 5.86230; 6.01111;
  
Sequence; FOXP3 [T04280]; 601; 606; 14.269340; GTGAAC; 5.86230; 6.01111;
  
Sequence; FOXP3 [T04280]; 723; 728; 9.512894; GTTCTG; 7.32788; 7.39742;
  
Sequence; FOXP3 [T04280]; 845; 850; 14.269340; GTTCAC; 5.86230; 6.01111;
  
Sequence; FOXP3 [T04280]; 900; 905; 4.756447; AAAAAC; 2.93115; 3.09069;
  
Sequence; FOXP3 [T04280]; 952; 957; 9.512894; GTTGGT; 7.32788; 7.39742;
  
Sequence; FOXP3 [T04280]; 995; 1000; 9.512894; GTTTAG; 7.32788; 7.39742;
  
Sequence; FOXP3 [T04280]; 1119; 1124; 9.512894; GGCAAC; 7.32788; 7.39742;
  
Sequence; FOXP3 [T04280]; 1241; 1246; 4.756447; GTTGAG; 2.93115; 3.09069;
  
Sequence; FOXP3 [T04280]; 1265; 1270; 0.000000; CACAAC; 1.46558; 1.46429;
  
Sequence; FOXP3 [T04280]; 1408; 1413; 14.269340; CCAAAC; 5.86230; 6.01111;
  
Sequence; FOXP3 [T04280]; 1444; 1449; 4.756447; CAAAAC; 2.93115; 3.09069;
  
Sequence; FOXP3 [T04280]; 1452; 1457; 9.512894; CTAAAC; 7.32788; 7.39742;
  
Sequence; FOXP3 [T04280]; 1462; 1467; 9.512894; GAGAAC; 7.32788; 7.39742;
  
Sequence; FOXP3 [T04280]; 1503; 1508; 9.512894; GTTATT; 7.32788; 7.39742;
  
Sequence; FOXP3 [T04280]; 1560; 1565; 14.269340; GTTTCC; 5.86230; 6.01111;
  
Sequence; FOXP3 [T04280]; 1614; 1619; 6.581441; TAAAAC; 0.97705; 1.05257;
  
Sequence; FOXP3 [T04280]; 1660; 1665; 1.824994; TACAAC; 0.48853; 0.49904;
  
Sequence; FOXP3 [T04280]; 1787; 1792; 14.269340; GTTAAT; 5.86230; 6.01111;
  
Sequence; FOXP3 [T04280]; 1848; 1853; 14.269340; GTTTGT; 5.86230; 6.01111;
  
Sequence; FOXP3 [T04280]; 1932; 1937; 9.512894; GTAAAC; 7.32788; 7.39742;
  
Sequence; PR B [T00696]; 91; 97; 11.148154; AACATGG; 2.19836; 2.39326;
  
Sequence; PR B [T00696]; 313; 319; 8.338824; AACAGAG; 1.09918; 1.16255;
  
Sequence; PR B [T00696]; 333; 339; 9.743489; AACACAC; 1.09918; 1.09361;
  
Sequence; PR B [T00696]; 470; 476; 9.743489; AACACGG; 1.09918; 1.09361;
  
Sequence; PR B [T00696]; 903; 909; 11.148154; AACAAGC; 2.19836; 2.39326;
  
Sequence; PR B [T00696]; 991; 997; 10.231719; ATGTGTT; 0.36639; 0.37067;
  
Sequence; PR B [T00696]; 1122; 1128; 11.148154; AACATAG; 2.19836; 2.39326;
  
Sequence; PR B [T00696]; 1268; 1274; 2.809330; AACATTG; 0.73279; 0.79898;
  
Sequence; PR B [T00696]; 1447; 1453; 10.231719; AACACCT; 0.36639; 0.37067;
  
Sequence; PR B [T00696]; 1465; 1471; 2.809330; AACATTG; 0.73279; 0.79898;
  
Sequence; PR B [T00696]; 1499; 1505; 8.338824; GCCTGTT; 1.09918; 1.16255;
  
Sequence; PR B [T00696]; 1663; 1669; 11.636384; AACAAAT; 0.73279; 0.79898;
  
Sequence; PR B [T00696]; 1836; 1842; 3.297560; AACATTT; 0.24426; 0.27240;
  
Sequence; PR B [T00696]; 1844; 1850; 8.827054; ACCTGTT; 0.36639; 0.38254;
  
Sequence; PR A [T01661]; 91; 97; 11.148154; AACATGG; 2.19836; 2.39326;
  
Sequence; PR A [T01661]; 313; 319; 8.338824; AACAGAG; 1.09918; 1.16255;
  
Sequence; PR A [T01661]; 333; 339; 9.743489; AACACAC; 1.09918; 1.09361;
  
Sequence; PR A [T01661]; 470; 476; 9.743489; AACACGG; 1.09918; 1.09361;
  
Sequence; PR A [T01661]; 903; 909; 11.148154; AACAAGC; 2.19836; 2.39326;
  
Sequence; PR A [T01661]; 991; 997; 10.231719; ATGTGTT; 0.36639; 0.37067;
  
Sequence; PR A [T01661]; 1122; 1128; 11.148154; AACATAG; 2.19836; 2.39326;
  
Sequence; PR A [T01661]; 1268; 1274; 2.809330; AACATTG; 0.73279; 0.79898;
  
Sequence; PR A [T01661]; 1447; 1453; 10.231719; AACACCT; 0.36639; 0.37067;
  
Sequence; PR A [T01661]; 1465; 1471; 2.809330; AACATTG; 0.73279; 0.79898;
  
Sequence; PR A [T01661]; 1499; 1505; 8.338824; GCCTGTT; 1.09918; 1.16255;
  
Sequence; PR A [T01661]; 1663; 1669; 11.636384; AACAAAT; 0.73279; 0.79898;
  
Sequence; PR A [T01661]; 1836; 1842; 3.297560; AACATTT; 0.24426; 0.27240;
  
Sequence; PR A [T01661]; 1844; 1850; 8.827054; ACCTGTT; 0.36639; 0.38254;
  
Sequence; RXR-alpha [T01345]; 48; 54; 4.423008; GGGTGGA; 0.24426; 0.23668;
  
Sequence; RXR-alpha [T01345]; 99; 105; 4.019014; GAAACCC; 0.97705; 0.91950;
  
Sequence; RXR-alpha [T01345]; 262; 268; 5.937582; GGGTGAC; 0.73279; 0.67890;
  
Sequence; RXR-alpha [T01345]; 285; 291; 6.967687; GGGTGGT; 0.36639; 0.33952;
  
Sequence; RXR-alpha [T01345]; 478; 484; 4.019014; GAAACCC; 0.97705; 0.91950;
  
Sequence; RXR-alpha [T01345]; 577; 583; 0.000000; TGAACCC; 0.24426; 0.24073;
  
Sequence; RXR-alpha [T01345]; 634; 640; 5.937582; GGGTGAC; 0.73279; 0.67890;
  
Sequence; RXR-alpha [T01345]; 794; 800; 7.815913; GGGTGGC; 0.24426; 0.22534;
  
Sequence; RXR-alpha [T01345]; 835; 841; 3.392904; GGGTCCT; 1.09918; 1.03617;
  
Sequence; RXR-alpha [T01345]; 856; 862; 3.392904; GGGTGAG; 1.09918; 1.03617;
  
Sequence; RXR-alpha [T01345]; 915; 921; 6.967687; GGGTGGT; 0.36639; 0.33952;
  
Sequence; RXR-alpha [T01345]; 1286; 1292; 5.937582; GGGTGAC; 0.73279; 0.67890;
  
Sequence; RXR-alpha [T01345]; 1298; 1304; 4.867240; GAGACCC; 0.48853; 0.45389;
  
Sequence; RXR-alpha [T01345]; 1379; 1385; 4.241130; GGGTAAG; 0.97705; 0.91950;
  
Sequence; RXR-alpha [T01345]; 1403; 1409; 4.241130; GGGACCC; 0.97705; 0.91950;
  
Sequence; RXR-alpha [T01345]; 1485; 1491; 6.119461; GGGTAGG; 0.73279; 0.67890;
  
Sequence; RAR-beta [T00721]; 97; 106; 6.415195; GTGAAACCCC; 0.18320; 0.17730;
  
Sequence; RAR-beta [T00721]; 476; 485; 6.415195; GTGAAACCCC; 0.18320; 0.17730;
  
Sequence; RAR-beta [T00721]; 575; 584; 5.370617; CATGAACCCA; 0.15266; 0.14233;
  
Sequence; YY1 [T00915]; 3; 6; 0.000000; ATGG; 7.81641; 7.79732;
  
Sequence; YY1 [T00915]; 94; 97; 0.000000; ATGG; 7.81641; 7.79732;
  
Sequence; YY1 [T00915]; 105; 108; 0.000000; CCAT; 7.81641; 7.79732;
  
Sequence; YY1 [T00915]; 274; 277; 0.000000; ATGG; 7.81641; 7.79732;
  
Sequence; YY1 [T00915]; 452; 455; 0.000000; ATGG; 7.81641; 7.79732;
  
Sequence; YY1 [T00915]; 459; 462; 0.000000; CCAT; 7.81641; 7.79732;
  
Sequence; YY1 [T00915]; 652; 655; 0.000000; CCAT; 7.81641; 7.79732;
  
Sequence; YY1 [T00915]; 771; 774; 0.000000; CCAT; 7.81641; 7.79732;
  
Sequence; YY1 [T00915]; 1004; 1007; 0.000000; CCAT; 7.81641; 7.79732;
  
Sequence; YY1 [T00915]; 1035; 1038; 0.000000; ATGG; 7.81641; 7.79732;
  
Sequence; YY1 [T00915]; 1176; 1179; 0.000000; ATGG; 7.81641; 7.79732;
  
Sequence; YY1 [T00915]; 1377; 1380; 0.000000; ATGG; 7.81641; 7.79732;
  
Sequence; YY1 [T00915]; 1392; 1395; 0.000000; ATGG; 7.81641; 7.79732;
  
Sequence; YY1 [T00915]; 1547; 1550; 0.000000; CCAT; 7.81641; 7.79732;
  
Sequence; YY1 [T00915]; 1756; 1759; 0.000000; ATGG; 7.81641; 7.79732;
  
Sequence; TFIID [T00820]; 32; 38; 9.552105; TTTGGGA; 1.46558; 1.57124;
  
Sequence; TFIID [T00820]; 112; 118; 5.544826; TACTAAA; 0.73279; 0.81388;
  
Sequence; TFIID [T00820]; 115; 121; 0.000000; TAAAAAA; 1.09918; 1.32476;
  
Sequence; TFIID [T00820]; 342; 348; 0.000000; TTTTAGA; 1.09918; 1.32476;
  
Sequence; TFIID [T00820]; 414; 420; 9.552105; TTTGGGA; 1.46558; 1.57124;
  
Sequence; TFIID [T00820]; 491; 497; 5.544826; TACTAAA; 0.73279; 0.81388;
  
Sequence; TFIID [T00820]; 500; 506; 1.537547; TACAAAA; 0.73279; 0.85283;
  
Sequence; TFIID [T00820]; 655; 661; 8.014558; TCTCAAA; 2.19836; 2.44070;
  
Sequence; TFIID [T00820]; 657; 663; 0.000000; TCAAAAA; 1.09918; 1.32476;
  
Sequence; TFIID [T00820]; 898; 904; 0.000000; TAAAAAA; 1.09918; 1.32476;
  
Sequence; TFIID [T00820]; 945; 951; 8.014558; TTTGTAA; 2.19836; 2.44070;
  
Sequence; TFIID [T00820]; 973; 979; 8.014558; TTTCCTA; 2.19836; 2.44070;
  
Sequence; TFIID [T00820]; 1007; 1013; 1.537547; TTCAAAA; 0.73279; 0.85283;
  
Sequence; TFIID [T00820]; 1008; 1014; 0.000000; TCAAAAA; 1.09918; 1.32476;
  
Sequence; TFIID [T00820]; 1064; 1070; 9.552105; TTTGGGA; 1.46558; 1.57124;
  
Sequence; TFIID [T00820]; 1104; 1110; 8.014558; TTTGAGA; 2.19836; 2.44070;
  
Sequence; TFIID [T00820]; 1144; 1150; 8.014558; TATCAAA; 2.19836; 2.44070;
  
Sequence; TFIID [T00820]; 1146; 1152; 0.000000; TCAAAAA; 1.09918; 1.32476;
  
Sequence; TFIID [T00820]; 1309; 1315; 8.014558; TCAGAAA; 2.19836; 2.44070;
  
Sequence; TFIID [T00820]; 1441; 1447; 8.014558; TCTCAAA; 2.19836; 2.44070;
  
Sequence; TFIID [T00820]; 1543; 1549; 1.537547; TTTTCCA; 0.73279; 0.85283;
  
Sequence; TFIID [T00820]; 1561; 1567; 8.014558; TTTCCAA; 2.19836; 2.44070;
  
Sequence; TFIID [T00820]; 1606; 1612; 0.000000; TTTTCAA; 1.09918; 1.32476;
  
Sequence; TFIID [T00820]; 1765; 1771; 8.014558; TTTCCTA; 2.19836; 2.44070;
  
Sequence; TFIID [T00820]; 1792; 1798; 0.000000; TTTTTTA; 1.09918; 1.32476;
  
Sequence; TFIID [T00820]; 1793; 1799; 0.000000; TTTTTAA; 1.09918; 1.32476;
  
Sequence; TFIID [T00820]; 1794; 1800; 4.007279; TTTTAAA; 1.09918; 1.26425;
  
Sequence; TFIID [T00820]; 1795; 1801; 0.000000; TTTAAAA; 1.09918; 1.32476;
  
Sequence; TFIID [T00820]; 1796; 1802; 0.000000; TTAAAAA; 1.09918; 1.32476;
  
Sequence; TFIID [T00820]; 1811; 1817; 8.014558; TTTCCTA; 2.19836; 2.44070;
  
Sequence; TFIID [T00820]; 1827; 1833; 9.552105; TTTCACA; 1.46558; 1.57124;
  
Sequence; TFIID [T00820]; 1849; 1855; 8.014558; TTTGTGA; 2.19836; 2.44070;
  
Sequence; TFIID [T00820]; 1924; 1930; 8.014558; TTTCCAA; 2.19836; 2.44070;
  
Sequence; TFIID [T00820]; 1961; 1967; 4.007279; TTTTAAA; 1.09918; 1.26425;
  
Sequence; TFIID [T00820]; 1986; 1992; 9.552105; TTTGTCA; 1.46558; 1.57124;
  
Sequence; MEF-2A [T01005]; 123; 133; 9.003254; AAAAAAAAATA; 0.07347; 0.08899;
  
Sequence; MEF-2A [T01005]; 491; 501; 9.220261; TACTAAAAATA; 0.07347; 0.08899;
  
Sequence; MEF-2A [T01005]; 1145; 1155; 6.342474; ATCAAAAAATA; 0.02385; 0.02989;
  
Sequence; MEF-2A [T01005]; 1151; 1161; 7.982343; AAATAAAAATA; 0.04007; 0.05051;
  
Sequence; MEF-2A [T01005]; 1594; 1604; 7.982343; AGGTTAAAATA; 0.04007; 0.05051;
  
Sequence; HNF-3alpha [T02512]; 56; 63; 7.000129; CATTTTGA; 0.82439; 0.97705;
  
Sequence; HNF-3alpha [T02512]; 126; 133; 3.500065; AAAAAATA; 0.27480; 0.34061;
  
Sequence; HNF-3alpha [T02512]; 339; 346; 7.000129; CATTTTTA; 0.82439; 0.97705;
  
Sequence; HNF-3alpha [T02512]; 360; 367; 10.500194; AATAAATT; 1.19078; 1.38019;
  
Sequence; HNF-3alpha [T02512]; 494; 501; 0.000000; TAAAAATA; 0.09160; 0.11373;
  
Sequence; HNF-3alpha [T02512]; 503; 510; 7.000129; AAAAAATT; 0.82439; 0.97705;
  
Sequence; HNF-3alpha [T02512]; 677; 684; 14.000258; AAGAAATT; 1.64877; 1.82548;
  
Sequence; HNF-3alpha [T02512]; 943; 950; 14.000258; GATTTGTA; 1.64877; 1.82548;
  
Sequence; HNF-3alpha [T02512]; 1148; 1155; 3.500065; AAAAAATA; 0.27480; 0.34061;
  
Sequence; HNF-3alpha [T02512]; 1154; 1161; 0.000000; TAAAAATA; 0.09160; 0.11373;
  
Sequence; HNF-3alpha [T02512]; 1160; 1167; 7.000129; TAAAAATG; 0.82439; 0.97705;
  
Sequence; HNF-3alpha [T02512]; 1597; 1604; 1.342935; TTAAAATA; 0.03053; 0.03761;
  
Sequence; HNF-3alpha [T02512]; 1663; 1670; 14.000258; AACAAATT; 1.64877; 1.82548;
  
Sequence; HNF-3alpha [T02512]; 1748; 1755; 14.000258; AGCAAATT; 1.64877; 1.82548;
  
Sequence; HNF-3alpha [T02512]; 1763; 1770; 14.000258; AATTTCCT; 1.64877; 1.82548;
  
Sequence; HNF-3alpha [T02512]; 1790; 1797; 7.000129; AATTTTTT; 0.82439; 0.97705;
  
Sequence; HNF-3alpha [T02512]; 1808; 1815; 14.000258; CATTTTCC; 1.64877; 1.82548;
  
Sequence; HNF-3alpha [T02512]; 1858; 1865; 10.500194; CATTTTTT; 1.19078; 1.38019;
  
Sequence; HNF-3alpha [T02512]; 1918; 1925; 10.500194; CGAAAATT; 1.19078; 1.38019;
  
Sequence; HNF-3alpha [T02512]; 1922; 1929; 10.500194; AATTTCCA; 1.19078; 1.38019;
  
Sequence; HNF-3alpha [T02512]; 1939; 1946; 14.000258; CATTTTCC; 1.64877; 1.82548;
  
Sequence; HNF-3alpha [T02512]; 1959; 1966; 4.842999; AATTTTAA; 0.09160; 0.11376;
  
Sequence; HNF-3alpha [T02512]; 1962; 1969; 11.843128; TTTAAATC; 0.39693; 0.46397;
  
Sequence; ENKTF-1 [T00255]; 61; 68; 12.629773; TGAGGCCA; 2.19836; 2.06420;
  
Sequence; ENKTF-1 [T00255]; 84; 91; 8.198520; CCTAGCCA; 0.73279; 0.64341;
  
Sequence; ENKTF-1 [T00255]; 139; 146; 12.629773; ATTAGCCA; 2.19836; 2.06420;
  
Sequence; ENKTF-1 [T00255]; 154; 161; 12.629773; TGGCATGT; 2.19836; 2.06420;
  
Sequence; ENKTF-1 [T00255]; 389; 396; 11.374018; TGGCTCAC; 1.09918; 1.07083;
  
Sequence; ENKTF-1 [T00255]; 465; 472; 12.629773; TGGCTAAC; 2.19836; 2.06420;
  
Sequence; ENKTF-1 [T00255]; 523; 530; 13.885529; TGGCAGGT; 1.09918; 0.99430;
  
Sequence; ENKTF-1 [T00255]; 612; 619; 6.942764; TCACGCCA; 1.46558; 1.31636;
  
Sequence; ENKTF-1 [T00255]; 797; 804; 5.687009; TGGCCTTG; 0.73279; 0.67237;
  
Sequence; ENKTF-1 [T00255]; 1024; 1031; 8.198520; CCTGGCCA; 0.73279; 0.64341;
  
Sequence; ENKTF-1 [T00255]; 1026; 1033; 8.198520; TGGCCAGG; 0.73279; 0.64341;
  
Sequence; ENKTF-1 [T00255]; 1039; 1046; 11.374018; TGGCTCAC; 1.09918; 1.07083;
  
Sequence; ENKTF-1 [T00255]; 1165; 1172; 11.374018; ATGAGCCA; 1.09918; 1.07083;
  
Sequence; ENKTF-1 [T00255]; 1185; 1192; 12.629773; TGGCACGC; 2.19836; 2.06420;
  
Sequence; ENKTF-1 [T00255]; 1358; 1365; 8.198520; CCTTGCCA; 0.73279; 0.64341;
  
Sequence; ENKTF-1 [T00255]; 1567; 1574; 12.629773; AGATGCCA; 2.19836; 2.06420;
  
Sequence; ENKTF-1 [T00255]; 1587; 1594; 12.629773; AGAAGCCA; 2.19836; 2.06420;
  
Sequence; ENKTF-1 [T00255]; 1681; 1688; 12.629773; TGGCCTCT; 2.19836; 2.06420;
  
Sequence; ENKTF-1 [T00255]; 1706; 1713; 11.374018; AAAAGCCA; 1.09918; 1.07083;
  
Sequence; ENKTF-1 [T00255]; 1888; 1895; 12.629773; TGGCCCCA; 2.19836; 2.06420;
  
Sequence; XBP-1 [T00902]; 53; 58; 8.756040; GATCAT; 2.93115; 3.15910;
  
Sequence; XBP-1 [T00902]; 154; 159; 6.478682; TGGCAT; 0.97705; 0.92444;
  
Sequence; XBP-1 [T00902]; 201; 206; 4.894955; ATGCCT; 0.97705; 0.92532;
  
Sequence; XBP-1 [T00902]; 238; 243; 8.756040; ATGATC; 2.93115; 3.15910;
  
Sequence; XBP-1 [T00902]; 348; 353; 8.756040; ATGATC; 2.93115; 3.15910;
  
Sequence; XBP-1 [T00902]; 365; 370; 7.172312; ATTCAT; 2.93115; 3.15216;
  
Sequence; XBP-1 [T00902]; 572; 577; 4.894955; CGGCAT; 0.97705; 0.92532;
  
Sequence; XBP-1 [T00902]; 576; 581; 8.756040; ATGAAC; 2.93115; 3.15910;
  
Sequence; XBP-1 [T00902]; 682; 687; 7.172312; ATTCAT; 2.93115; 3.15216;
  
Sequence; XBP-1 [T00902]; 773; 778; 8.756040; ATGAGC; 2.93115; 3.15910;
  
Sequence; XBP-1 [T00902]; 866; 871; 8.756040; ATGAGC; 2.93115; 3.15910;
  
Sequence; XBP-1 [T00902]; 888; 893; 7.172312; ATGAAT; 2.93115; 3.15216;
  
Sequence; XBP-1 [T00902]; 1017; 1022; 6.478682; GGGCAT; 0.97705; 0.92444;
  
Sequence; XBP-1 [T00902]; 1031; 1036; 4.894955; AGGCAT; 0.97705; 0.92532;
  
Sequence; XBP-1 [T00902]; 1110; 1115; 8.756040; ATGAGC; 2.93115; 3.15910;
  
Sequence; XBP-1 [T00902]; 1165; 1170; 8.756040; ATGAGC; 2.93115; 3.15910;
  
Sequence; XBP-1 [T00902]; 1172; 1177; 4.894955; AGGCAT; 0.97705; 0.92532;
  
Sequence; XBP-1 [T00902]; 1272; 1277; 13.650994; TTGCAT; 2.93115; 2.88747;
  
Sequence; XBP-1 [T00902]; 1328; 1333; 0.000000; AGTCAT; 0.97705; 0.98059;
  
Sequence; XBP-1 [T00902]; 1517; 1522; 12.067266; ATGCAG; 2.93115; 2.88658;
  
Sequence; XBP-1 [T00902]; 1549; 1554; 1.583727; ATGACA; 0.97705; 0.98155;
  
Sequence; XBP-1 [T00902]; 1569; 1574; 6.478682; ATGCCA; 0.97705; 0.92444;
  
Sequence; XBP-1 [T00902]; 1654; 1659; 12.067266; CTGCAT; 2.93115; 2.88658;
  
Sequence; XBP-1 [T00902]; 1696; 1701; 13.650994; ATGCAA; 2.93115; 2.88747;
  
Sequence; XBP-1 [T00902]; 1771; 1776; 12.067266; ATGCTG; 2.93115; 2.88658;
  
Sequence; XBP-1 [T00902]; 1805; 1810; 8.756040; TTTCAT; 2.93115; 3.15910;
  
Sequence; XBP-1 [T00902]; 1855; 1860; 4.894955; AGGCAT; 0.97705; 0.92532;
  
Sequence; XBP-1 [T00902]; 1936; 1941; 7.172312; ACTCAT; 2.93115; 3.15216;
  
Sequence; XBP-1 [T00902]; 1991; 1996; 12.067266; CAGCAT; 2.93115; 2.88658;
  
Sequence; RAR-beta:RXR-alpha [T05420]; 202; 213; 9.970660; TGCCTTGAGCCC; 0.06977; 0.06353;
  
Sequence; RAR-beta:RXR-alpha [T05420]; 803; 814; 14.955991; TGCACAGGGCCC; 0.20967; 0.19188;
  
Sequence; RAR-beta:RXR-alpha [T05420]; 1087; 1098; 7.477995; TCACTTGAGCCC; 0.02862; 0.02599;
  
Sequence; RAR-beta:RXR-alpha [T05420]; 1249; 1260; 12.463326; TGCAGTGATCCC; 0.13096; 0.11957;
  
Sequence; Pax-5 [T00070]; 207; 213; 8.014558; TGAGCCC; 2.19836; 1.92429;
  
Sequence; Pax-5 [T00070]; 278; 284; 0.000000; GGGCAGG; 1.09918; 0.94708;
  
Sequence; Pax-5 [T00070]; 299; 305; 1.537547; GGGCGGG; 0.73279; 0.65690;
  
Sequence; Pax-5 [T00070]; 374; 380; 8.014558; GAGGCCC; 2.19836; 1.92429;
  
Sequence; Pax-5 [T00070]; 515; 521; 1.537547; GGGCGTG; 0.73279; 0.65690;
  
Sequence; Pax-5 [T00070]; 742; 748; 0.000000; GGGCAGG; 1.09918; 0.94708;
  
Sequence; Pax-5 [T00070]; 750; 756; 4.007279; AGGGCCC; 1.09918; 0.97478;
  
Sequence; Pax-5 [T00070]; 751; 757; 8.014558; GGGCCCA; 2.19836; 1.92429;
  
Sequence; Pax-5 [T00070]; 766; 772; 8.014558; GCTGCCC; 2.19836; 1.92429;
  
Sequence; Pax-5 [T00070]; 774; 780; 8.014558; TGAGCCC; 2.19836; 1.92429;
  
Sequence; Pax-5 [T00070]; 808; 814; 4.007279; AGGGCCC; 1.09918; 0.97478;
  
Sequence; Pax-5 [T00070]; 809; 815; 8.014558; GGGCCCA; 2.19836; 1.92429;
  
Sequence; Pax-5 [T00070]; 911; 917; 1.537547; GGGCGGG; 0.73279; 0.65690;
  
Sequence; Pax-5 [T00070]; 923; 929; 4.007279; GGGCACT; 1.09918; 0.97478;
  
Sequence; Pax-5 [T00070]; 938; 944; 8.014558; GGGCTGA; 2.19836; 1.92429;
  
Sequence; Pax-5 [T00070]; 1017; 1023; 4.007279; GGGCATT; 1.09918; 0.97478;
  
Sequence; Pax-5 [T00070]; 1092; 1098; 8.014558; TGAGCCC; 2.19836; 1.92429;
  
Sequence; Pax-5 [T00070]; 1207; 1213; 8.014558; GGGCTGA; 2.19836; 1.92429;
  
Sequence; Pax-5 [T00070]; 1367; 1373; 8.014558; GGGCTTC; 2.19836; 1.92429;
  
Sequence; Pax-5 [T00070]; 1527; 1533; 8.014558; GGAGCCC; 2.19836; 1.92429;
  
Sequence; Pax-5 [T00070]; 1717; 1723; 8.014558; GCAGCCC; 2.19836; 1.92429;
  
Sequence; Pax-5 [T00070]; 1887; 1893; 9.552105; TTGGCCC; 1.46558; 1.31664;
  
Sequence; p53 [T00671]; 207; 213; 6.778774; TGAGCCC; 1.09918; 0.94199;
  
Sequence; p53 [T00671]; 278; 284; 0.000000; GGGCAGG; 0.36639; 0.35124;
  
Sequence; p53 [T00671]; 299; 305; 3.375208; GGGCGGG; 0.73279; 0.67702;
  
Sequence; p53 [T00671]; 374; 380; 6.938545; GAGGCCC; 1.09918; 0.94199;
  
Sequence; p53 [T00671]; 515; 521; 3.375208; GGGCGTG; 0.73279; 0.67702;
  
Sequence; p53 [T00671]; 742; 748; 0.000000; GGGCAGG; 0.36639; 0.35124;
  
Sequence; p53 [T00671]; 750; 756; 8.912104; AGGGCCC; 0.12213; 0.10156;
  
Sequence; p53 [T00671]; 751; 757; 7.153797; GGGCCCA; 1.09918; 0.94199;
  
Sequence; p53 [T00671]; 766; 772; 2.813291; GCTGCCC; 0.48853; 0.43942;
  
Sequence; p53 [T00671]; 774; 780; 6.778774; TGAGCCC; 1.09918; 0.94199;
  
Sequence; p53 [T00671]; 808; 814; 8.912104; AGGGCCC; 0.12213; 0.10156;
  
Sequence; p53 [T00671]; 809; 815; 7.153797; GGGCCCA; 1.09918; 0.94199;
  
Sequence; p53 [T00671]; 911; 917; 3.375208; GGGCGGG; 0.73279; 0.67702;
  
Sequence; p53 [T00671]; 923; 929; 4.786849; GGGCACT; 0.48853; 0.43749;
  
Sequence; p53 [T00671]; 938; 944; 5.508538; GGGCTGA; 0.61066; 0.56484;
  
Sequence; p53 [T00671]; 1017; 1023; 3.516613; GGGCATT; 0.73279; 0.67702;
  
Sequence; p53 [T00671]; 1092; 1098; 6.778774; TGAGCCC; 1.09918; 0.94199;
  
Sequence; p53 [T00671]; 1207; 1213; 5.508538; GGGCTGA; 0.61066; 0.56484;
  
Sequence; p53 [T00671]; 1367; 1373; 6.563521; GGGCTTC; 0.48853; 0.42171;
  
Sequence; p53 [T00671]; 1527; 1533; 7.833758; GGAGCCC; 0.48853; 0.40647;
  
Sequence; p53 [T00671]; 1717; 1723; 6.563521; GCAGCCC; 0.48853; 0.42171;
  
Sequence; p53 [T00671]; 1887; 1893; 6.095267; TTGGCCC; 0.61066; 0.52826;
  
Sequence; T3R-beta1 [T00851]; 92; 100; 6.702681; ACATGGTGA; 0.21373; 0.20361;
  
Sequence; T3R-beta1 [T00851]; 242; 250; 1.110682; TCACCACTG; 0.07633; 0.07322;
  
Sequence; T3R-beta1 [T00851]; 259; 267; 7.774776; CCTGGGTGA; 0.27480; 0.26463;
  
Sequence; T3R-beta1 [T00851]; 285; 293; 2.259951; GGGTGGTGA; 0.03053; 0.03320;
  
Sequence; T3R-beta1 [T00851]; 471; 479; 11.145411; ACACGGTGA; 0.03053; 0.02996;
  
Sequence; T3R-beta1 [T00851]; 631; 639; 7.774776; CCTGGGTGA; 0.27480; 0.26463;
  
Sequence; T3R-beta1 [T00851]; 853; 861; 5.553412; CTGGGGTGA; 0.21373; 0.21316;
  
Sequence; T3R-beta1 [T00851]; 1283; 1291; 7.774776; CCTGGGTGA; 0.27480; 0.26463;
  
Sequence; T3R-beta1 [T00851]; 1420; 1428; 4.481316; TCACCTGAT; 0.27480; 0.28253;
  
Sequence; GR [T05076]; 29; 35; 11.290546; CACTTTG; 1.46558; 1.52028;
  
Sequence; GR [T05076]; 56; 62; 7.527031; CATTTTG; 1.83197; 2.00587;
  
Sequence; GR [T05076]; 321; 327; 5.207533; CAAAACC; 0.24426; 0.24824;
  
Sequence; GR [T05076]; 411; 417; 11.290546; CACTTTG; 1.46558; 1.52028;
  
Sequence; GR [T05076]; 502; 508; 0.000000; CAAAAAA; 0.36639; 0.44629;
  
Sequence; GR [T05076]; 658; 664; 0.000000; CAAAAAA; 0.36639; 0.44629;
  
Sequence; GR [T05076]; 942; 948; 11.290546; TGATTTG; 1.46558; 1.52028;
  
Sequence; GR [T05076]; 1009; 1015; 0.000000; CAAAAAG; 0.36639; 0.44629;
  
Sequence; GR [T05076]; 1061; 1067; 7.527031; AGCTTTG; 1.83197; 2.00587;
  
Sequence; GR [T05076]; 1147; 1153; 0.000000; CAAAAAA; 0.36639; 0.44629;
  
Sequence; GR [T05076]; 1444; 1450; 3.763516; CAAAACA; 0.73279; 0.81070;
  
Sequence; GR [T05076]; 1699; 1705; 7.527031; CAAAGCA; 1.83197; 2.00587;
  
Sequence; GR [T05076]; 1704; 1710; 0.000000; CAAAAAG; 0.36639; 0.44629;
  
Sequence; GR [T05076]; 1778; 1784; 11.290546; AACTTTG; 1.46558; 1.52028;
  
Sequence; GR [T05076]; 1846; 1852; 7.527031; CTGTTTG; 1.83197; 2.00587;
  
Sequence; GR [T05076]; 1983; 1989; 3.763516; ATCTTTG; 0.73279; 0.81070;
  
Sequence; STAT4 [T01577]; 183; 188; 4.411765; GGAAGC; 1.95410; 2.05835;
  
Sequence; STAT4 [T01577]; 305; 310; 2.941176; GGAAGT; 2.93115; 3.08471;
  
Sequence; STAT4 [T01577]; 311; 316; 2.941176; GGAACA; 2.93115; 3.08471;
  
Sequence; STAT4 [T01577]; 352; 357; 4.411765; TCTTCC; 1.95410; 2.05835;
  
Sequence; STAT4 [T01577]; 648; 653; 2.941176; GATTCC; 2.93115; 3.08471;
  
Sequence; STAT4 [T01577]; 690; 695; 1.470588; GGAAAA; 1.95410; 2.11563;
  
Sequence; STAT4 [T01577]; 713; 718; 5.882353; GGAAGG; 0.48853; 0.53907;
  
Sequence; STAT4 [T01577]; 718; 723; 5.882353; GGAAGG; 0.48853; 0.53907;
  
Sequence; STAT4 [T01577]; 972; 977; 1.470588; TTTTCC; 1.95410; 2.11563;
  
Sequence; STAT4 [T01577]; 1000; 1005; 2.941176; GGTTCC; 2.93115; 3.08471;
  
Sequence; STAT4 [T01577]; 1020; 1025; 4.411765; CATTCC; 1.95410; 2.05835;
  
Sequence; STAT4 [T01577]; 1100; 1105; 1.470588; GGAATT; 1.95410; 2.11563;
  
Sequence; STAT4 [T01577]; 1331; 1336; 4.411765; CATTCC; 1.95410; 2.05835;
  
Sequence; STAT4 [T01577]; 1369; 1374; 4.411765; GCTTCC; 1.95410; 2.05835;
  
Sequence; STAT4 [T01577]; 1543; 1548; 1.470588; TTTTCC; 1.95410; 2.11563;
  
Sequence; STAT4 [T01577]; 1560; 1565; 1.470588; GTTTCC; 1.95410; 2.11563;
  
Sequence; STAT4 [T01577]; 1584; 1589; 4.411765; GGAAGA; 1.95410; 2.05835;
  
Sequence; STAT4 [T01577]; 1639; 1644; 2.941176; CTTTCC; 2.93115; 3.08471;
  
Sequence; STAT4 [T01577]; 1764; 1769; 0.000000; ATTTCC; 0.48853; 0.54617;
  
Sequence; STAT4 [T01577]; 1810; 1815; 1.470588; TTTTCC; 1.95410; 2.11563;
  
Sequence; STAT4 [T01577]; 1834; 1839; 2.941176; GGAACA; 2.93115; 3.08471;
  
Sequence; STAT4 [T01577]; 1871; 1876; 4.411765; GGAAGA; 1.95410; 2.05835;
  
Sequence; STAT4 [T01577]; 1923; 1928; 0.000000; ATTTCC; 0.48853; 0.54617;
  
Sequence; STAT4 [T01577]; 1941; 1946; 1.470588; TTTTCC; 1.95410; 2.11563;
  
Sequence; c-Ets-1 [T00112]; 181; 187; 5.430224; CGGGAAG; 0.36639; 0.40890;
  
Sequence; c-Ets-1 [T00112]; 303; 309; 5.558311; GGGGAAG; 0.36639; 0.40890;
  
Sequence; c-Ets-1 [T00112]; 309; 315; 8.244941; GTGGAAC; 0.24426; 0.23368;
  
Sequence; c-Ets-1 [T00112]; 353; 359; 4.654478; CTTCCAG; 0.85492; 0.92763;
  
Sequence; c-Ets-1 [T00112]; 649; 655; 9.065503; ATTCCAT; 0.85492; 0.87658;
  
Sequence; c-Ets-1 [T00112]; 688; 694; 7.071349; AGGGAAA; 0.73279; 0.80642;
  
Sequence; c-Ets-1 [T00112]; 711; 717; 5.686398; AGGGAAG; 0.36639; 0.40890;
  
Sequence; c-Ets-1 [T00112]; 716; 722; 5.686398; AGGGAAG; 0.36639; 0.40890;
  
Sequence; c-Ets-1 [T00112]; 973; 979; 1.769212; TTTCCTA; 0.12213; 0.14678;
  
Sequence; c-Ets-1 [T00112]; 1001; 1007; 8.373028; GTTCCAT; 0.24426; 0.24788;
  
Sequence; c-Ets-1 [T00112]; 1021; 1027; 4.154851; ATTCCTG; 0.24426; 0.25361;
  
Sequence; c-Ets-1 [T00112]; 1098; 1104; 4.154851; CAGGAAT; 0.24426; 0.25361;
  
Sequence; c-Ets-1 [T00112]; 1332; 1338; 4.154851; ATTCCTG; 0.24426; 0.25361;
  
Sequence; c-Ets-1 [T00112]; 1370; 1376; 0.256174; CTTCCTT; 0.24426; 0.29471;
  
Sequence; c-Ets-1 [T00112]; 1544; 1550; 6.295602; TTTCCAT; 0.48853; 0.50162;
  
Sequence; c-Ets-1 [T00112]; 1561; 1567; 6.423689; TTTCCAA; 0.48853; 0.50162;
  
Sequence; c-Ets-1 [T00112]; 1582; 1588; 5.038739; TTGGAAG; 0.48853; 0.52180;
  
Sequence; c-Ets-1 [T00112]; 1640; 1646; 6.943262; TTTCCCC; 0.73279; 0.80642;
  
Sequence; c-Ets-1 [T00112]; 1765; 1771; 1.769212; TTTCCTA; 0.12213; 0.14678;
  
Sequence; c-Ets-1 [T00112]; 1811; 1817; 1.769212; TTTCCTA; 0.12213; 0.14678;
  
Sequence; c-Ets-1 [T00112]; 1832; 1838; 3.462376; CAGGAAC; 0.61066; 0.63879;
  
Sequence; c-Ets-1 [T00112]; 1869; 1875; 4.654478; CTGGAAG; 0.85492; 0.92763;
  
Sequence; c-Ets-1 [T00112]; 1924; 1930; 6.423689; TTTCCAA; 0.48853; 0.50162;
  
Sequence; c-Ets-1 [T00112]; 1942; 1948; 7.071349; TTTCCCT; 0.73279; 0.80642;
  
Sequence; Elk-1 [T00250]; 179; 187; 12.179139; CTCGGGAAG; 0.24426; 0.26773;
  
Sequence; Elk-1 [T00250]; 301; 309; 10.962309; GCGGGGAAG; 0.26716; 0.27992;
  
Sequence; Elk-1 [T00250]; 353; 361; 12.179139; CTTCCAGAA; 0.24426; 0.26773;
  
Sequence; Elk-1 [T00250]; 709; 717; 11.919333; CAAGGGAAG; 0.24426; 0.26773;
  
Sequence; Elk-1 [T00250]; 714; 722; 11.784986; GAAGGGAAG; 0.24426; 0.26773;
  
Sequence; Elk-1 [T00250]; 1370; 1378; 4.204473; CTTCCTTAT; 0.09160; 0.10674;
  
Sequence; Elk-1 [T00250]; 1580; 1588; 12.742011; ACTTGGAAG; 0.12213; 0.12861;
  
Sequence; Elk-1 [T00250]; 1867; 1875; 11.096657; TCCTGGAAG; 0.26716; 0.27992;
  
Sequence; PPAR-alpha:RXR-alpha [T05221]; 374; 384; 8.384593; GAGGCCCAGGC; 0.02004; 0.01780;
  
Sequence; PPAR-alpha:RXR-alpha [T05221]; 536; 546; 6.515440; TAGTCCCAGCT; 0.03721; 0.03206;
  
Sequence; PPAR-alpha:RXR-alpha [T05221]; 808; 818; 12.041567; AGGGCCCAGGC; 0.00572; 0.00481;
  
Sequence; PPAR-alpha:RXR-alpha [T05221]; 1399; 1409; 10.013453; TGCTGGGACCC; 0.08587; 0.07058;
  
Sequence; PPAR-alpha:RXR-alpha [T05221]; 1471; 1481; 14.659739; GTCCCCCAGTG; 0.15457; 0.15059;
  
Sequence; PPAR-alpha:RXR-alpha [T05221]; 1528; 1538; 6.515440; GAGCCCCAGAT; 0.03721; 0.03206;
  
Sequence; AhR [T01795]; 392; 402; 8.184723; CTCACGCCTGT; 0.04866; 0.04717;
  
Sequence; AhR [T01795]; 611; 621; 9.058053; ATCACGCCACT; 0.03244; 0.03181;
  
Sequence; AhR:Arnt [T05394]; 144; 153; 8.006459; CCAGGCGTGG; 0.01717; 0.01458;
  
Sequence; AhR:Arnt [T05394]; 393; 402; 10.816794; TCACGCCTGT; 0.15457; 0.13460;
  
Sequence; AhR:Arnt [T05394]; 513; 522; 6.928166; CCGGGCGTGG; 0.05152; 0.04188;
  
Sequence; AhR:Arnt [T05394]; 612; 621; 12.548836; TCACGCCACT; 0.25762; 0.22360;
  
Sequence; AhR:Arnt [T05394]; 1187; 1196; 12.548836; GCACGCACCT; 0.25762; 0.22360;
  
Sequence; c-Jun [T00133]; 265; 271; 6.787369; TGACAGA; 0.73279; 0.71774;
  
Sequence; c-Jun [T00133]; 441; 447; 8.242207; GAGGTCA; 0.48853; 0.49014;
  
Sequence; c-Jun [T00133]; 637; 643; 6.787369; TGACAGA; 0.73279; 0.71774;
  
Sequence; c-Jun [T00133]; 697; 703; 8.832178; AAGGTCA; 0.61066; 0.59738;
  
Sequence; c-Jun [T00133]; 929; 935; 7.937147; TGACTAG; 0.48853; 0.49014;
  
Sequence; c-Jun [T00133]; 1289; 1295; 6.787369; TGACAGA; 0.73279; 0.71774;
  
Sequence; c-Jun [T00133]; 1326; 1332; 5.783074; AAAGTCA; 0.36639; 0.39597;
  
Sequence; c-Jun [T00133]; 1550; 1556; 10.917170; TGACAGG; 0.36639; 0.34625;
  
Sequence; c-Jun [T00133]; 1898; 1904; 4.441904; TGACTGA; 0.12213; 0.12108;
  
Sequence; c-Jun [T00133]; 1902; 1908; 9.397655; TGACAAT; 0.73279; 0.69974;
  
Sequence; c-Jun [T00133]; 1986; 1992; 6.152811; TTTGTCA; 0.36639; 0.39597;
  
Sequence; COUP-TF1 [T00149]; 438; 450; 14.375592; CACGAGGTCAGGA; 0.05009; 0.04917;
  
Sequence; COUP-TF1 [T00149]; 694; 706; 13.640068; AAGAAGGTCAGAG; 0.04186; 0.04121;
  
Sequence; COUP-TF1 [T00149]; 1678; 1690; 14.883141; CTGTGGCCTCTCC; 0.01610; 0.01646;
  
Sequence; PEA3 [T00685]; 457; 465; 7.421728; GACCATCCT; 0.34349; 0.32887;
  
Sequence; PEA3 [T00685]; 988; 996; 1.194633; AGGATGTGT; 0.06870; 0.06829;
  
Sequence; HNF-1C [T01951]; 464; 472; 12.499603; CTGGCTAAC; 0.09160; 0.09849;
  
Sequence; HNF-1C [T01951]; 1503; 1511; 13.908254; GTTATTCTG; 0.32823; 0.34268;
  
Sequence; HNF-1C [T01951]; 1596; 1604; 13.826644; GTTAAAATA; 0.32823; 0.34268;
  
Sequence; HNF-1C [T01951]; 1787; 1795; 7.229698; GTTAATTTT; 0.08397; 0.09322;
  
Sequence; HOXD9 [T01424]; 498; 507; 10.220007; AATACAAAAA; 0.23281; 0.28298;
  
Sequence; HOXD9 [T01424]; 896; 905; 5.275652; AATAAAAAAC; 0.04580; 0.06171;
  
Sequence; HOXD9 [T01424]; 1152; 1161; 0.000000; AATAAAAATA; 0.01145; 0.01549;
  
Sequence; HOXD9 [T01424]; 1158; 1167; 0.954221; AATAAAAATG; 0.01527; 0.01985;
  
Sequence; HOXD9 [T01424]; 1499; 1508; 9.356547; GCCTGTTATT; 0.02290; 0.02700;
  
Sequence; HOXD9 [T01424]; 1622; 1631; 10.220007; TCTTGATATT; 0.23281; 0.28298;
  
Sequence; HOXD10 [T01425]; 498; 507; 10.220007; AATACAAAAA; 0.23281; 0.28298;
  
Sequence; HOXD10 [T01425]; 896; 905; 5.275652; AATAAAAAAC; 0.04580; 0.06171;
  
Sequence; HOXD10 [T01425]; 1152; 1161; 0.000000; AATAAAAATA; 0.01145; 0.01549;
  
Sequence; HOXD10 [T01425]; 1158; 1167; 0.954221; AATAAAAATG; 0.01527; 0.01985;
  
Sequence; HOXD10 [T01425]; 1499; 1508; 9.356547; GCCTGTTATT; 0.02290; 0.02700;
  
Sequence; HOXD10 [T01425]; 1622; 1631; 10.220007; TCTTGATATT; 0.23281; 0.28298;
  
Sequence; VDR [T00885]; 9; 17; 4.617121; GTTCACGCC; 0.37403; 0.37003;
  
Sequence; VDR [T00885]; 573; 581; 6.925682; GGCATGAAC; 0.42746; 0.43308;
  
Sequence; VDR [T00885]; 598; 606; 4.617121; GCAGTGAAC; 0.37403; 0.37003;
  
Sequence; VDR [T00885]; 845; 853; 4.617121; GTTCACTGC; 0.37403; 0.37003;
  
Sequence; PXR-1:RXR-alpha [T05671]; 6; 13; 5.726524; GTGGTTCA; 0.06107; 0.05875;
  
Sequence; PXR-1:RXR-alpha [T05671]; 577; 584; 3.395883; TGAACCCA; 0.12213; 0.12115;
  
Sequence; PXR-1:RXR-alpha [T05671]; 602; 609; 0.000000; TGAACTAA; 0.12213; 0.12844;
  
Sequence; PXR-1:RXR-alpha [T05671]; 842; 849; 6.544600; TACGTTCA; 0.24426; 0.26139;
  
Sequence; c-Myb [T00137]; 68; 75; 12.979731; AGGAGTTC; 0.54959; 0.56540;
  
Sequence; c-Myb [T00137]; 603; 610; 14.459319; GAACTAAG; 0.36639; 0.38094;
  
Sequence; c-Myb [T00137]; 948; 955; 6.157321; GTAAGTTG; 0.21373; 0.21711;
  
Sequence; c-Myb [T00137]; 1410; 1417; 13.142679; AAACTAGG; 0.54959; 0.56540;
  
Sequence; c-Myb [T00137]; 1454; 1461; 9.110773; AAACTTGG; 0.39693; 0.40824;
  
Sequence; c-Myb [T00137]; 1556; 1563; 6.040180; GACAGTTT; 0.21373; 0.21711;
  
Sequence; c-Myb [T00137]; 1616; 1623; 8.728118; AAACTTTC; 0.30533; 0.31835;
  
Sequence; c-Myb [T00137]; 1777; 1784; 11.712811; AAACTTTG; 0.42746; 0.44215;
  
Sequence; c-Myb [T00137]; 1783; 1790; 12.979731; TGTAGTTA; 0.54959; 0.56540;
  
Sequence; IRF-1 [T00423]; 686; 694; 1.616539; ATAGGGAAA; 0.04580; 0.06498;
  
Sequence; IRF-1 [T00423]; 973; 981; 5.823972; TTTCCTAAT; 0.16793; 0.19558;
  
Sequence; IRF-1 [T00423]; 1544; 1552; 5.564062; TTTCCATGA; 0.22900; 0.26692;
  
Sequence; IRF-1 [T00423]; 1561; 1569; 8.497322; TTTCCAAGA; 0.20610; 0.22317;
  
Sequence; IRF-1 [T00423]; 1640; 1648; 7.041849; TTTCCCCAG; 0.12976; 0.14725;
  
Sequence; IRF-1 [T00423]; 1765; 1773; 6.280446; TTTCCTATG; 0.19083; 0.21114;
  
Sequence; IRF-1 [T00423]; 1811; 1819; 4.207433; TTTCCTATT; 0.12976; 0.15966;
  
Sequence; IRF-1 [T00423]; 1924; 1932; 6.535281; TTTCCAATG; 0.19083; 0.21114;
  
Sequence; IRF-1 [T00423]; 1942; 1950; 3.689552; TTTCCCTCG; 0.06870; 0.08767;
  
Sequence; NF-AT1 [T00550]; 690; 698; 7.095752; GGAAAAGAA; 0.15266; 0.20017;
  
Sequence; NF-AT1 [T00550]; 969; 977; 6.562181; TCTTTTTCC; 0.03817; 0.05050;
  
Sequence; NF-AT1 [T00550]; 1540; 1548; 2.449764; AGCTTTTCC; 0.09160; 0.10221;
  
Sequence; NF-AT1 [T00550]; 1557; 1565; 6.003834; ACAGTTTCC; 0.06870; 0.08564;
  
Sequence; NF-AT1 [T00550]; 1636; 1644; 5.774030; TATCTTTCC; 0.06870; 0.08564;
  
Sequence; NF-AT1 [T00550]; 1761; 1769; 10.067740; GTAATTTCC; 0.12213; 0.12542;
  
Sequence; NF-AT1 [T00550]; 1807; 1815; 8.532897; TCATTTTCC; 0.10686; 0.12787;
  
Sequence; NF-AT1 [T00550]; 1920; 1928; 3.917696; AAAATTTCC; 0.07633; 0.08494;
  
Sequence; NF-AT1 [T00550]; 1938; 1946; 8.532897; TCATTTTCC; 0.10686; 0.12787;
  
Sequence; NFI/CTF [T00094]; 29; 36; 10.579747; CACTTTGG; 0.67172; 0.65084;
  
Sequence; NFI/CTF [T00094]; 411; 418; 10.579747; CACTTTGG; 0.67172; 0.65084;
  
Sequence; NFI/CTF [T00094]; 708; 715; 5.021086; CCAAGGGA; 0.24426; 0.23114;
  
Sequence; NFI/CTF [T00094]; 755; 762; 13.146004; CCAAGAAT; 1.09918; 1.12358;
  
Sequence; NFI/CTF [T00094]; 1061; 1068; 9.352332; AGCTTTGG; 0.54959; 0.52387;
  
Sequence; NFI/CTF [T00094]; 1363; 1370; 3.793671; CCAAGGGC; 0.18320; 0.16872;
  
Sequence; NFI/CTF [T00094]; 1408; 1415; 6.786076; CCAAACTA; 0.73279; 0.70115;
  
Sequence; NFI/CTF [T00094]; 1454; 1461; 13.146004; AAACTTGG; 1.09918; 1.12358;
  
Sequence; NFI/CTF [T00094]; 1564; 1571; 14.373419; CCAAGATG; 1.46558; 1.49169;
  
Sequence; NFI/CTF [T00094]; 1578; 1585; 13.146004; GTACTTGG; 1.09918; 1.12358;
  
Sequence; NFI/CTF [T00094]; 1723; 1730; 6.786076; CCAAGCTG; 0.73279; 0.70115;
  
Sequence; NFI/CTF [T00094]; 1814; 1821; 8.814757; CCTATTGG; 0.48853; 0.48192;
  
Sequence; NFI/CTF [T00094]; 1883; 1890; 6.786076; CTGATTGG; 0.73279; 0.70115;
  
Sequence; NFI/CTF [T00094]; 1893; 1900; 8.814757; CCAAGTGA; 0.48853; 0.48192;
  
Sequence; NFI/CTF [T00094]; 1927; 1934; 10.579747; CCAATGTA; 0.67172; 0.65084;
  
Sequence; EBF [T05427]; 803; 813; 10.637427; TGCACAGGGCC; 0.07061; 0.06429;
  
Sequence; USF2 [T00878]; 526; 535; 5.052423; CAGGTGCCTG; 0.10305; 0.09783;
  
Sequence; USF2 [T00878]; 814; 823; 5.052423; CAGGCACCTG; 0.10305; 0.09783;
  
Sequence; USF2 [T00878]; 1042; 1051; 9.056375; CTCACACCTG; 0.15457; 0.14558;
  
Sequence; USF2 [T00878]; 1417; 1426; 5.052423; GCCTCACCTG; 0.10305; 0.09783;
  
Sequence; USF2 [T00878]; 1569; 1578; 4.528187; ATGCCACCTG; 0.06870; 0.06294;
  
Sequence; USF2 [T00878]; 1671; 1680; 4.528187; CGGACACCTG; 0.06870; 0.06294;
  
Sequence; C/EBPalpha [T00105]; 862; 868; 6.460799; GGCAATG; 0.48853; 0.49007;
  
Sequence; C/EBPalpha [T00105]; 876; 882; 1.761449; TATTGTG; 0.48853; 0.50496;
  
Sequence; C/EBPalpha [T00105]; 893; 899; 6.245236; TTCAATA; 0.97705; 1.02558;
  
Sequence; C/EBPalpha [T00105]; 1270; 1276; 7.465744; CATTGCA; 0.48853; 0.52003;
  
Sequence; C/EBPalpha [T00105]; 1467; 1473; 7.001740; CATTGTC; 0.73279; 0.77665;
  
Sequence; C/EBPalpha [T00105]; 1816; 1822; 4.776286; TATTGGT; 0.97705; 1.04662;
  
Sequence; C/EBPalpha [T00105]; 1885; 1891; 4.560723; GATTGGC; 0.24426; 0.23529;
  
Sequence; C/EBPalpha [T00105]; 1903; 1909; 4.560723; GACAATC; 0.24426; 0.23529;
  
Sequence; C/EBPalpha [T00105]; 1926; 1932; 8.006685; TCCAATG; 0.24426; 0.24951;
  
Sequence; C/EBPalpha [T00105]; 1956; 1962; 4.845599; AGCAATT; 0.97705; 1.04662;
  
Sequence; C/EBPalpha [T00105]; 1994; 2000; 7.465744; CATTGCA; 0.48853; 0.52003;
  
Sequence; AR [T00040]; 877; 885; 7.623968; ATTGTGTCC; 0.25190; 0.25405;
  
Sequence; AR [T00040]; 1431; 1439; 5.953985; GACCTGTCC; 0.24426; 0.24417;
  
Sequence; AR [T00040]; 1466; 1474; 2.376830; ACATTGTCC; 0.11450; 0.11595;
  
Sequence; AR [T00040]; 1555; 1563; 2.733525; GGACAGTTT; 0.11450; 0.11595;
  
Sequence; AR [T00040]; 1672; 1680; 8.055836; GGACACCTG; 0.19846; 0.19684;
  
Sequence; NF-AT2 [T01945]; 690; 699; 13.964020; GGAAAAGAAG; 0.09732; 0.11126;
  
Sequence; NF-AT2 [T01945]; 968; 977; 9.999272; CTCTTTTTCC; 0.08778; 0.10655;
  
Sequence; NF-AT2 [T01945]; 1539; 1548; 7.779688; CAGCTTTTCC; 0.08969; 0.10181;
  
Sequence; NF-AT2 [T01945]; 1556; 1565; 6.245826; GACAGTTTCC; 0.04198; 0.04722;
  
Sequence; NF-AT2 [T01945]; 1635; 1644; 7.726527; CTATCTTTCC; 0.01717; 0.02073;
  
Sequence; NF-AT2 [T01945]; 1760; 1769; 13.476987; TGTAATTTCC; 0.07633; 0.08681;
  
Sequence; NF-AT2 [T01945]; 1806; 1815; 12.430157; TTCATTTTCC; 0.07633; 0.08924;
  
Sequence; NF-AT2 [T01945]; 1919; 1928; 1.659787; GAAAATTTCC; 0.01717; 0.01967;
  
Sequence; NF-AT2 [T01945]; 1937; 1946; 11.659060; CTCATTTTCC; 0.07252; 0.08657;
  
Sequence; STAT1beta [T01573]; 972; 981; 14.492168; TTTTCCTAAT; 0.20610; 0.24618;
  
Sequence; STAT1beta [T01573]; 1543; 1552; 6.908963; TTTTCCATGA; 0.10305; 0.11408;
  
Sequence; STAT1beta [T01573]; 1560; 1569; 5.796867; GTTTCCAAGA; 0.15457; 0.16958;
  
Sequence; STAT1beta [T01573]; 1639; 1648; 14.492168; CTTTCCCCAG; 0.20610; 0.24618;
  
Sequence; STAT1beta [T01573]; 1764; 1773; 11.593735; ATTTCCTATG; 0.30914; 0.35496;
  
Sequence; STAT1beta [T01573]; 1810; 1819; 14.492168; TTTTCCTATT; 0.20610; 0.24618;
  
Sequence; STAT1beta [T01573]; 1923; 1932; 11.593735; ATTTCCAATG; 0.30914; 0.35496;
  
Sequence; c-Ets-2 [T00113]; 974; 982; 7.841160; TTCCTAATT; 0.32059; 0.35201;
  
Sequence; c-Ets-2 [T00113]; 1022; 1030; 8.912323; TTCCTGGCC; 0.27480; 0.27956;
  
Sequence; c-Ets-2 [T00113]; 1095; 1103; 8.912323; GCCCAGGAA; 0.27480; 0.27956;
  
Sequence; c-Ets-2 [T00113]; 1333; 1341; 7.841160; TTCCTGAAA; 0.32059; 0.35201;
  
Sequence; c-Ets-2 [T00113]; 1371; 1379; 4.091811; TTCCTTATG; 0.16030; 0.20108;
  
Sequence; c-Ets-2 [T00113]; 1766; 1774; 7.766350; TTCCTATGC; 0.32059; 0.35201;
  
Sequence; c-Ets-2 [T00113]; 1812; 1820; 6.695187; TTCCTATTG; 0.09160; 0.10954;
  
Sequence; c-Ets-2 [T00113]; 1829; 1837; 6.695187; TCACAGGAA; 0.09160; 0.10954;
  
Sequence; GATA-1 [T00306]; 1138; 1143; 0.105011; TATCTC; 0.97705; 1.05714;
  
Sequence; GATA-1 [T00306]; 1144; 1149; 1.038567; TATCAA; 1.95410; 2.08017;
  
Sequence; GATA-1 [T00306]; 1424; 1429; 0.758539; CTGATA; 1.95410; 2.08017;
  
Sequence; GATA-1 [T00306]; 1624; 1629; 1.038567; TTGATA; 1.95410; 2.08017;
  
Sequence; GATA-1 [T00306]; 1636; 1641; 0.280028; TATCTT; 0.97705; 1.12137;
  
Sequence; GATA-1 [T00306]; 1728; 1733; 0.758539; CTGATA; 1.95410; 2.08017;
  
Sequence; GATA-1 [T00306]; 1978; 1983; 0.105011; GAGATA; 0.97705; 1.05714;
  
Sequence; GATA-1 [T00306]; 1982; 1987; 0.280028; TATCTT; 0.97705; 1.12137;
  
Sequence; GATA-2 [T00308]; 1140; 1148; 3.333333; TCTCTATCA; 0.30533; 0.33302;
  
Sequence; GATA-2 [T00308]; 1632; 1640; 2.222222; ACTCTATCT; 0.22900; 0.25177;
  
Sequence; AP-1 [T00029]; 1324; 1332; 13.922717; AAAAAGTCA; 0.33586; 0.34306;
  
Sequence; AP-1 [T00029]; 1898; 1906; 9.343400; TGACTGACA; 0.09160; 0.09670;
  
Sequence; NF-AT1 [T01948]; 1540; 1549; 4.823485; AGCTTTTCCA; 0.07633; 0.08541;
  
Sequence; NF-AT1 [T01948]; 1557; 1566; 4.134416; ACAGTTTCCA; 0.08397; 0.09365;
  
Sequence; NF-AT1 [T01948]; 1920; 1929; 2.067208; AAAATTTCCA; 0.03817; 0.04236;
  
Sequence; RBP-Jkappa [T01616]; 1542; 1553; 14.966990; CTTTTCCATGAC; 0.07967; 0.08045;
  
Sequence; SRY [T00997]; 31; 39; 13.352057; CTTTGGGAG; 0.24426; 0.24219;
  
Sequence; SRY [T00997]; 413; 421; 13.352057; CTTTGGGAG; 0.24426; 0.24219;
  
Sequence; SRY [T00997]; 1063; 1071; 13.352057; CTTTGGGAG; 0.24426; 0.24219;
  
Sequence; SRY [T00997]; 1695; 1703; 11.263007; TATGCAAAG; 0.12213; 0.12117;
  
Sequence; SRY [T00997]; 1780; 1788; 4.087393; CTTTGTAGT; 0.12213; 0.13465;
  
Sequence; SRY [T00997]; 1985; 1993; 7.175614; CTTTGTCAG; 0.30533; 0.32999;
  
Sequence; TCF-4E [T02878]; 31; 37; 12.604771; CTTTGGG; 0.48853; 0.49957;
  
Sequence; TCF-4E [T02878]; 413; 419; 12.604771; CTTTGGG; 0.48853; 0.49957;
  
Sequence; TCF-4E [T02878]; 1063; 1069; 12.604771; CTTTGGG; 0.48853; 0.49957;
  
Sequence; TCF-4E [T02878]; 1697; 1703; 3.151193; TGCAAAG; 0.24426; 0.26139;
  
Sequence; TCF-4E [T02878]; 1780; 1786; 9.453578; CTTTGTA; 0.48853; 0.50369;
  
Sequence; TCF-4E [T02878]; 1985; 1991; 12.604771; CTTTGTC; 0.48853; 0.49957;
  
Sequence; NF-Y [T00150]; 1817; 1824; 0.680578; ATTGGTCT; 0.09160; 0.09397;
  
Sequence; NF-Y [T00150]; 1886; 1893; 1.749852; ATTGGCCC; 0.18320; 0.18427;
  
Sequence; NF-Y [T00150]; 1924; 1931; 4.186615; TTTCCAAT; 0.18320; 0.19598;
  
Sequence; CTF [T00174]; 860; 871; 14.231922; GAGGCAATGAGC; 0.03864; 0.03683;
  
Sequence; CTF [T00174]; 1882; 1893; 6.560434; GCTGATTGGCCC; 0.02004; 0.01952;
  
Sequence; IRF-2 [T01491]; 1087; 1092; 0.000000; TCACTT; 0.48853; 0.54617;
  
Sequence; IRF-2 [T01491]; 1224; 1229; 0.000000; TCACTT; 0.48853; 0.54617;
  
Sequence; IRF-2 [T01491]; 1895; 1900; 0.000000; AAGTGA; 0.48853; 0.54617;
  
Sequence; RelA [T00594]; 1939; 1949; 14.045590; CATTTTCCCTC; 0.04580; 0.04772;
  
Sequence; HNF-1A [T00368]; 1596; 1603; 0.000000; GTTAAAAT; 0.24426; 0.28371;
  
Sequence; HNF-1A [T00368]; 1787; 1794; 0.143882; GTTAATTT; 0.24426; 0.28371;
  
Sequence; HNF-1B [T01950]; 1595; 1603; 14.123286; GGTTAAAAT; 0.12213; 0.12789;
  
Sequence; HNF-1B [T01950]; 1786; 1794; 8.241969; AGTTAATTT; 0.06870; 0.07386;
  
Sequence; POU2F1 [T00641]; 1693; 1703; 13.078878; TCTATGCAAAG; 0.22900; 0.25015;
  
Sequence; POU2F2 (Oct-2.1) [T00646]; 1655; 1665; 9.350233; TGCATTACAAC; 0.03435; 0.03746;
  
Sequence; GATA-3 [T00311]; 1631; 1642; 10.254584; TACTCTATCTTT; 0.01407; 0.01587;
  
Sequence; ETF [T00270]; 292; 302; 3.630042; GAGGGGGGGGC; 0.00239; 0.00224;
  
Sequence; ETF [T00270]; 744; 754; 10.493812; GCAGGCAGGGC; 0.15028; 0.12683;
  
Sequence; ETF [T00270]; 916; 926; 13.117265; GGTGGTGGGGC; 0.23043; 0.19617;
  
Sequence; ETF [T00270]; 1200; 1210; 10.493812; GCTAGGGGGGC; 0.15028; 0.12683;
  
Sequence; E2F-1 [T01542]; 301; 308; 5.476857; GCGGGGAA; 0.03053; 0.03138;
  
Sequence; E2F-1 [T01542]; 432; 439; 10.134154; GCGGATCA; 0.33586; 0.29208;
  
Sequence; E2F-1 [T01542]; 589; 596; 12.316610; GCGGAGCT; 0.54959; 0.47396;
  
Sequence; E2F-1 [T01542]; 913; 920; 11.888116; GCGGGTGG; 0.45799; 0.38864;
  
Sequence; E2F-1 [T01542]; 1181; 1188; 13.578236; GCGGTGGC; 0.73279; 0.62340;
  
Sequence; PU.1 [T02068]; 984; 996; 14.895952; GCTGAGGATGTGT; 0.04395; 0.05012;
  
Sequence; Sp1 [T00759]; 298; 307; 0.574521; GGGGCGGGGA; 0.00763; 0.00699;
  
Sequence; Sp1 [T00759]; 910; 919; 2.379823; AGGGCGGGTG; 0.04007; 0.03792;
  
Sequence; NF-kappaB [T00590]; 716; 727; 14.301366; AGGGAAGGTTCT; 0.04091; 0.03863;
  
Sequence; ER-alpha [T00261]; 443; 447; 0.000000; GGTCA; 1.95410; 1.84975;
  
Sequence; ER-alpha [T00261]; 699; 703; 0.000000; GGTCA; 1.95410; 1.84975;
  
Sequence; NF-kappaB1 [T00593]; 96; 106; 14.733751; GGTGAAACCCC; 0.01717; 0.01458;
  
Sequence; NF-kappaB1 [T00593]; 475; 485; 14.733751; GGTGAAACCCC; 0.01717; 0.01458;
  
Sequence; MAZ [T00490]; 286; 298; 14.918658; GGTGGTGAGGGGG; 0.06387; 0.07487;
  
Sequence; MAZ [T00490]; 295; 307; 14.134899; GGGGGGGCGGGGA; 0.03006; 0.03582;
  
Sequence; WT1 [T00899]; 295; 303; 11.111111; GGGGGGGCG; 0.20610; 0.17411;
  
Sequence; Egr-3 [T00243]; 42; 54; 11.366723; TGAGGTGGGTGGA; 0.02880; 0.02804;
  
  
-- END ---------------------------------------------------------------
  
