# Supplementary material for: CDK5RAP3 acts as a tumour suppressor in gastric cancer through the infiltration and polarization of tumour-associated macrophages
Source: Cancer Gene Ther. 2022 Aug 23;30(1):22–37. doi: 10.1038/s41417-022-00515-9 (PMC9842504; doi:10.1038/s41417-022-00515-9)
Supplement: Supplementary file 6 — Predicted binding sites for IL10 [file 41417_2022_515_MOESM6_ESM.html]

searchRes.txt
-- Input sequence ---------------------------------------------------
  
>Sequence
  
GTTAGACCTGCAGGCTAACACAGACACAGCCCAGAAAACCCAGGAGCCGAGGGGGAAGGAGAAAGGTGCAAGAAGGGGAAACCCAGGTCCTGGTCCCCTTCTCTCTGCTTCCTGGCAGCAGAACTCAGACAGAACCCTTAAGCCAGTCTAAGTCTGGCAGGACCAGTAAGTTCTGAGTTAGCTCCATACTAGTTTCTAGCAGGCTCTTTCTCACTTCCTGATTCTTAGGTTTCTACATTGACACTCCCTGAAGAGTTGGGAAGAGACACCACAGTCCCCTGACCCTGATCCATAGGTCACACAGCAGGGACATCCACAGGGTGGGCGTGGGCCCTCTCATCCCTCCCTCCCACTCACTTCACGCTGGCTGGGCCCCAAGGTGTTTGCACCCCTTGCAGTGAGTGACCTTCTCTAGTGCAGCAAGCTCAGAACCTGCTGCCACTGGAGTTGTCCCATTGCTGATGCAGAAAGGTGAAGAACTAGCAGAACACTGGAAATGCCCTCCATCTGGGTCCATGGCTACTTAAGCTCAATGCTCCCTGGCAGGCAGGAGGACAGGTGCTATTGCCCTGTTGGGACAGATGAAAAACAGACACAGGGAGGATGAGTGATTTGCCCTGACTATAGAGTGGCAGGGCCAAGGCAGAGCCCAGGCCTCCTGCACCTAGGTCAGTGTTCCTCCCAGTTACAGTCTAAACTGGAATGGCAGGCAAAGCCCCTGTGGAAGGGGAAGGTGAAGGCTCAATCAAAGGATCCCCAGAGACTTTCCAGATATCTGAAGAAGTCCTGATGTCACTGCCCCGGTCCTTCCCCAGGTAGAGCAACACTCCTCGCCGCAACCCAACTGGCTCCCCTTACCTTCTACACACACACACACACACACACACACACACACACACACACACACAAATCCAAGACAACACTACTAAGGCTTCTTTGGGAAGGGGAAGTAGGGATAGGTAAGAGGAAAGTAAGGGACCTCCTATCCAGCCTCCATGGAATCCTGACTTCTTTTCCTTGTTA  
TTTCAACTTCTTCCACCCCATCTTTTAAACTTTAGACTCCAGCCACAGAAGCTTACAACTAAAAGAAACTCTAAGGCCAATTTAATCCAAGGTTTCATTCTATGTGCTGGAGATGGTGTACAGTAGGGTGAGGAAACCAAATTCTCAGTTGGCACTGGTGTACCCTTGTACAGGTGATGTAATATCTCTGTGCCTCAGTTTGCTCACTATAAAATAGAGACGGTAGGGGTCATGGTGAGCACTACCTGACTAGCATATAAGAAGCTTTCAGCAAGTGCAGACTACTCTTACCCACTTCCCCCAAGCACAGTTGGGGTGGGGGACAGCTGAAGAGGTGGAAACATGTGCCTGAGAATCCTAATGAAATCGGGGTAAAGGAGCCTGGAACACATCCTGTGACCCCGCCTGTACTGTAGGAAGCCAGTCTCTGGAAAGTAAAATGGAAGGGCTGCTTGGGAACTTTGAGGATATTTAGCCCACCCCCTCATTTTTACTTGGGGAAACTAAGGCCCAGAGACCTAAGGTGACTGCCTAAGTTAGCAAGGAGAAGTCTTGGGTATTCATCCCAGGTTGGGGGGACCCAATTATTTCTCAATCCCATTGTATTCTGGAATGGGCAATTTGTCCACGTCACTGTGACCTAGGAACACGCGAATGAGAACCCACAGCTGAGGGCCTCTGCGCACAGAACAGCTGTTCTCCCCAGGAAATCAACTTTTTTTAATTGAGAAGCTAAAAAATTATTCTAAGAGAGGTAGCCCATCCTAAAAATAGCTGTAATGCAGAAGTTCATGTTCAACCAATCATTTTTGCTTACGATGCAAAAATTGAAAACTAAGTTTATTAGAGAGGTTAGAGAAGGAGGAGCTCTAAGCAGAAAAAATCCTGTGCCGGGAAACCTTGATTGTGGCTTTTTAATGAATGAAGAGGCCTCCCTGAGCTTACAATATAAAAGGGGGACAGAGAGGTGAAGGTCTA
  
  
-- Factors predicted by PROMO in this sequence ----------------------
  
NAME; MATRIX\_WIDTH;
  
GR-alpha [T00337]; 5
  
AP-2alphaA [T00035]; 6
  
PR B [T00696]; 7
  
PR A [T01661]; 7
  
Pax-5 [T00070]; 7
  
p53 [T00671]; 7
  
FOXP3 [T04280]; 6
  
RXR-alpha [T01345]; 7
  
RAR-beta [T00721]; 10
  
IRF-1 [T00423]; 9
  
NF-AT1 [T00550]; 9
  
TFII-I [T00824]; 6
  
STAT4 [T01577]; 6
  
c-Ets-1 [T00112]; 7
  
Elk-1 [T00250]; 9
  
ELF-1 [T01113]; 13
  
ENKTF-1 [T00255]; 8
  
c-Myb [T00137]; 8
  
YY1 [T00915]; 4
  
PU.1 [T02068]; 13
  
C/EBPbeta [T00581]; 4
  
C/EBPalpha [T00105]; 7
  
NF-1 [T00539]; 8
  
ER-alpha [T00261]; 5
  
RAR-alpha1 [T00719]; 13
  
c-Jun [T00133]; 7
  
COUP-TF1 [T00149]; 13
  
PEA3 [T00685]; 9
  
EBF [T05427]; 11
  
MAZ [T00490]; 13
  
Egr-3 [T00243]; 13
  
Ik-1 [T02702]; 13
  
AhR [T01795]; 11
  
AhR:Arnt [T05394]; 10
  
NFI/CTF [T00094]; 8
  
AR [T00040]; 9
  
PPAR-alpha:RXR-alpha [T05221]; 11
  
XBP-1 [T00902]; 6
  
HNF-3alpha [T02512]; 8
  
GR-beta [T01920]; 5
  
TFIID [T00820]; 7
  
ETF [T00270]; 11
  
SRY [T00997]; 9
  
TCF-4E [T02878]; 7
  
GR [T05076]; 7
  
RAR-beta:RXR-alpha [T05420]; 12
  
NF-AT2 [T01945]; 10
  
NF-AT1 [T01948]; 10
  
STAT1beta [T01573]; 10
  
GATA-1 [T00306]; 6
  
NF-kappaB [T00590]; 12
  
NF-kappaB1 [T00593]; 11
  
RelA [T00594]; 11
  
E2F-1 [T01542]; 8
  
LEF-1 [T02905]; 8
  
c-Ets-2 [T00113]; 9
  
STAT5A [T04683]; 13
  
POU2F2 (Oct-2.1) [T00646]; 11
  
TBP [T00794]; 10
  
MEF-2A [T01005]; 11
  
HNF-4alpha [T03828]; 13
  
TCF-4 [T02918]; 10
  
CREB [T00163]; 9
  
ATF-2 [T00167]; 10
  
ATF [T00051]; 12
  
ATF3 [T01313]; 8
  
GCF [T00320]; 9
  
HOXD9 [T01424]; 10
  
HOXD10 [T01425]; 10
  
T3R-beta1 [T00851]; 9
  
HNF-1C [T01951]; 9
  
HNF-1B [T01950]; 9
  
POU2F1 [T00641]; 11
  
NF-Y [T00150]; 8
  
VDR [T00885]; 9
  
PXR-1:RXR-alpha [T05671]; 8
  
ATF-1 [T00968]; 11
  
AP-1 [T00029]; 9
  
RBP-Jkappa [T01616]; 12
  
USF2 [T00878]; 10
  
CTF [T00174]; 12
  
IRF-2 [T01491]; 6
  
  
-- PROMO predictions detail ------------------------------------------
  
  
Sequence name; Factor name; Start position; End position; Dissimilarity; String; RE equally; RE query
  
Sequence; GR-alpha [T00337]; 6; 10; 8.073878; CCTGC; 7.81641; 7.49230;
  
Sequence; GR-alpha [T00337]; 9; 13; 8.073878; GCAGG; 7.81641; 7.49230;
  
Sequence; GR-alpha [T00337]; 39; 43; 8.073878; CCAGG; 7.81641; 7.49230;
  
Sequence; GR-alpha [T00337]; 47; 51; 8.281568; CGAGG; 7.81641; 7.53757;
  
Sequence; GR-alpha [T00337]; 54; 58; 8.281568; GAAGG; 7.81641; 7.53757;
  
Sequence; GR-alpha [T00337]; 61; 65; 0.207689; AAAGG; 7.81641; 7.83842;
  
Sequence; GR-alpha [T00337]; 71; 75; 8.281568; GAAGG; 7.81641; 7.53757;
  
Sequence; GR-alpha [T00337]; 82; 86; 8.073878; CCAGG; 7.81641; 7.49230;
  
Sequence; GR-alpha [T00337]; 88; 92; 8.073878; CCTGG; 7.81641; 7.49230;
  
Sequence; GR-alpha [T00337]; 96; 100; 8.281568; CCTTC; 7.81641; 7.53757;
  
Sequence; GR-alpha [T00337]; 110; 114; 8.073878; CCTGG; 7.81641; 7.49230;
  
Sequence; GR-alpha [T00337]; 135; 139; 6.263098; CCTTA; 3.90820; 3.85665;
  
Sequence; GR-alpha [T00337]; 156; 160; 8.073878; GCAGG; 7.81641; 7.49230;
  
Sequence; GR-alpha [T00337]; 198; 202; 8.073878; GCAGG; 7.81641; 7.49230;
  
Sequence; GR-alpha [T00337]; 216; 220; 6.055408; CCTGA; 3.90820; 3.86993;
  
Sequence; GR-alpha [T00337]; 224; 228; 6.055408; TTAGG; 3.90820; 3.86993;
  
Sequence; GR-alpha [T00337]; 246; 250; 6.055408; CCTGA; 3.90820; 3.86993;
  
Sequence; GR-alpha [T00337]; 277; 281; 6.055408; CCTGA; 3.90820; 3.86993;
  
Sequence; GR-alpha [T00337]; 283; 287; 6.055408; CCTGA; 3.90820; 3.86993;
  
Sequence; GR-alpha [T00337]; 291; 295; 0.000000; ATAGG; 7.81641; 7.83842;
  
Sequence; GR-alpha [T00337]; 303; 307; 8.073878; GCAGG; 7.81641; 7.49230;
  
Sequence; GR-alpha [T00337]; 315; 319; 0.000000; ACAGG; 7.81641; 7.83842;
  
Sequence; GR-alpha [T00337]; 332; 336; 0.207689; CCTCT; 7.81641; 7.83842;
  
Sequence; GR-alpha [T00337]; 341; 345; 8.281568; CCTCC; 7.81641; 7.53757;
  
Sequence; GR-alpha [T00337]; 345; 349; 8.281568; CCTCC; 7.81641; 7.53757;
  
Sequence; GR-alpha [T00337]; 375; 379; 8.281568; CAAGG; 7.81641; 7.53757;
  
Sequence; GR-alpha [T00337]; 390; 394; 8.281568; CCTTG; 7.81641; 7.53757;
  
Sequence; GR-alpha [T00337]; 405; 409; 8.281568; CCTTC; 7.81641; 7.53757;
  
Sequence; GR-alpha [T00337]; 431; 435; 8.073878; CCTGC; 7.81641; 7.49230;
  
Sequence; GR-alpha [T00337]; 467; 471; 0.207689; AAAGG; 7.81641; 7.83842;
  
Sequence; GR-alpha [T00337]; 500; 504; 8.281568; CCTCC; 7.81641; 7.53757;
  
Sequence; GR-alpha [T00337]; 538; 542; 8.073878; CCTGG; 7.81641; 7.49230;
  
Sequence; GR-alpha [T00337]; 542; 546; 8.073878; GCAGG; 7.81641; 7.49230;
  
Sequence; GR-alpha [T00337]; 546; 550; 8.073878; GCAGG; 7.81641; 7.49230;
  
Sequence; GR-alpha [T00337]; 549; 553; 8.281568; GGAGG; 7.81641; 7.53757;
  
Sequence; GR-alpha [T00337]; 554; 558; 0.000000; ACAGG; 7.81641; 7.83842;
  
Sequence; GR-alpha [T00337]; 568; 572; 0.000000; CCTGT; 7.81641; 7.83842;
  
Sequence; GR-alpha [T00337]; 594; 598; 0.000000; ACAGG; 7.81641; 7.83842;
  
Sequence; GR-alpha [T00337]; 598; 602; 8.281568; GGAGG; 7.81641; 7.53757;
  
Sequence; GR-alpha [T00337]; 616; 620; 6.055408; CCTGA; 3.90820; 3.86993;
  
Sequence; GR-alpha [T00337]; 631; 635; 8.073878; GCAGG; 7.81641; 7.49230;
  
Sequence; GR-alpha [T00337]; 638; 642; 8.281568; CAAGG; 7.81641; 7.53757;
  
Sequence; GR-alpha [T00337]; 649; 653; 8.073878; CCAGG; 7.81641; 7.49230;
  
Sequence; GR-alpha [T00337]; 654; 658; 8.281568; CCTCC; 7.81641; 7.53757;
  
Sequence; GR-alpha [T00337]; 657; 661; 8.073878; CCTGC; 7.81641; 7.49230;
  
Sequence; GR-alpha [T00337]; 663; 667; 8.073878; CCTAG; 7.81641; 7.49230;
  
Sequence; GR-alpha [T00337]; 664; 668; 8.073878; CTAGG; 7.81641; 7.49230;
  
Sequence; GR-alpha [T00337]; 677; 681; 8.281568; CCTCC; 7.81641; 7.53757;
  
Sequence; GR-alpha [T00337]; 705; 709; 8.073878; GCAGG; 7.81641; 7.49230;
  
Sequence; GR-alpha [T00337]; 717; 721; 0.000000; CCTGT; 7.81641; 7.83842;
  
Sequence; GR-alpha [T00337]; 723; 727; 8.281568; GAAGG; 7.81641; 7.53757;
  
Sequence; GR-alpha [T00337]; 729; 733; 8.281568; GAAGG; 7.81641; 7.53757;
  
Sequence; GR-alpha [T00337]; 735; 739; 8.281568; GAAGG; 7.81641; 7.53757;
  
Sequence; GR-alpha [T00337]; 747; 751; 0.207689; AAAGG; 7.81641; 7.83842;
  
Sequence; GR-alpha [T00337]; 785; 789; 6.055408; CCTGA; 3.90820; 3.86993;
  
Sequence; GR-alpha [T00337]; 805; 809; 8.281568; CCTTC; 7.81641; 7.53757;
  
Sequence; GR-alpha [T00337]; 811; 815; 8.073878; CCAGG; 7.81641; 7.49230;
  
Sequence; GR-alpha [T00337]; 828; 832; 8.281568; CCTCG; 7.81641; 7.53757;
  
Sequence; GR-alpha [T00337]; 852; 856; 6.263098; CCTTA; 3.90820; 3.85665;
  
Sequence; GR-alpha [T00337]; 857; 861; 8.281568; CCTTC; 7.81641; 7.53757;
  
Sequence; GR-alpha [T00337]; 926; 930; 6.263098; TAAGG; 3.90820; 3.85665;
  
Sequence; GR-alpha [T00337]; 940; 944; 8.281568; GAAGG; 7.81641; 7.53757;
  
Sequence; GR-alpha [T00337]; 949; 953; 8.073878; GTAGG; 7.81641; 7.49230;
  
Sequence; GR-alpha [T00337]; 955; 959; 0.000000; ATAGG; 7.81641; 7.83842;
  
Sequence; GR-alpha [T00337]; 962; 966; 0.207689; AGAGG; 7.81641; 7.83842;
  
Sequence; GR-alpha [T00337]; 971; 975; 6.263098; TAAGG; 3.90820; 3.85665;
  
Sequence; GR-alpha [T00337]; 978; 982; 8.281568; CCTCC; 7.81641; 7.53757;
  
Sequence; GR-alpha [T00337]; 981; 985; 0.000000; CCTAT; 7.81641; 7.83842;
  
Sequence; GR-alpha [T00337]; 990; 994; 8.281568; CCTCC; 7.81641; 7.53757;
  
Sequence; GR-alpha [T00337]; 1002; 1006; 6.055408; CCTGA; 3.90820; 3.86993;
  
Sequence; GR-alpha [T00337]; 1015; 1019; 8.281568; CCTTG; 7.81641; 7.53757;
  
Sequence; GR-alpha [T00337]; 1094; 1098; 6.263098; TAAGG; 3.90820; 3.85665;
  
Sequence; GR-alpha [T00337]; 1110; 1114; 8.281568; CAAGG; 7.81641; 7.53757;
  
Sequence; GR-alpha [T00337]; 1145; 1149; 8.073878; GTAGG; 7.81641; 7.49230;
  
Sequence; GR-alpha [T00337]; 1151; 1155; 6.263098; TGAGG; 3.90820; 3.85665;
  
Sequence; GR-alpha [T00337]; 1186; 1190; 8.281568; CCTTG; 7.81641; 7.53757;
  
Sequence; GR-alpha [T00337]; 1192; 1196; 0.000000; ACAGG; 7.81641; 7.83842;
  
Sequence; GR-alpha [T00337]; 1215; 1219; 6.263098; CCTCA; 3.90820; 3.85665;
  
Sequence; GR-alpha [T00337]; 1245; 1249; 8.073878; GTAGG; 7.81641; 7.49230;
  
Sequence; GR-alpha [T00337]; 1267; 1271; 6.055408; CCTGA; 3.90820; 3.86993;
  
Sequence; GR-alpha [T00337]; 1353; 1357; 0.207689; AGAGG; 7.81641; 7.83842;
  
Sequence; GR-alpha [T00337]; 1370; 1374; 6.055408; CCTGA; 3.90820; 3.86993;
  
Sequence; GR-alpha [T00337]; 1379; 1383; 6.055408; CCTAA; 3.90820; 3.86993;
  
Sequence; GR-alpha [T00337]; 1396; 1400; 0.207689; AAAGG; 7.81641; 7.83842;
  
Sequence; GR-alpha [T00337]; 1403; 1407; 8.073878; CCTGG; 7.81641; 7.49230;
  
Sequence; GR-alpha [T00337]; 1415; 1419; 0.000000; CCTGT; 7.81641; 7.83842;
  
Sequence; GR-alpha [T00337]; 1427; 1431; 0.000000; CCTGT; 7.81641; 7.83842;
  
Sequence; GR-alpha [T00337]; 1435; 1439; 8.073878; GTAGG; 7.81641; 7.49230;
  
Sequence; GR-alpha [T00337]; 1465; 1469; 8.281568; GAAGG; 7.81641; 7.53757;
  
Sequence; GR-alpha [T00337]; 1485; 1489; 6.263098; TGAGG; 3.90820; 3.85665;
  
Sequence; GR-alpha [T00337]; 1505; 1509; 6.263098; CCTCA; 3.90820; 3.85665;
  
Sequence; GR-alpha [T00337]; 1527; 1531; 6.263098; TAAGG; 3.90820; 3.85665;
  
Sequence; GR-alpha [T00337]; 1540; 1544; 6.055408; CCTAA; 3.90820; 3.86993;
  
Sequence; GR-alpha [T00337]; 1542; 1546; 6.263098; TAAGG; 3.90820; 3.85665;
  
Sequence; GR-alpha [T00337]; 1553; 1557; 6.055408; CCTAA; 3.90820; 3.86993;
  
Sequence; GR-alpha [T00337]; 1563; 1567; 8.281568; CAAGG; 7.81641; 7.53757;
  
Sequence; GR-alpha [T00337]; 1588; 1592; 8.073878; CCAGG; 7.81641; 7.49230;
  
Sequence; GR-alpha [T00337]; 1662; 1666; 8.073878; CCTAG; 7.81641; 7.49230;
  
Sequence; GR-alpha [T00337]; 1663; 1667; 8.073878; CTAGG; 7.81641; 7.49230;
  
Sequence; GR-alpha [T00337]; 1692; 1696; 6.263098; TGAGG; 3.90820; 3.85665;
  
Sequence; GR-alpha [T00337]; 1698; 1702; 0.207689; CCTCT; 7.81641; 7.83842;
  
Sequence; GR-alpha [T00337]; 1725; 1729; 8.073878; CCAGG; 7.81641; 7.49230;
  
Sequence; GR-alpha [T00337]; 1773; 1777; 0.207689; AGAGG; 7.81641; 7.83842;
  
Sequence; GR-alpha [T00337]; 1786; 1790; 6.055408; CCTAA; 3.90820; 3.86993;
  
Sequence; GR-alpha [T00337]; 1870; 1874; 0.207689; AGAGG; 7.81641; 7.83842;
  
Sequence; GR-alpha [T00337]; 1880; 1884; 8.281568; GAAGG; 7.81641; 7.53757;
  
Sequence; GR-alpha [T00337]; 1883; 1887; 8.281568; GGAGG; 7.81641; 7.53757;
  
Sequence; GR-alpha [T00337]; 1907; 1911; 0.000000; CCTGT; 7.81641; 7.83842;
  
Sequence; GR-alpha [T00337]; 1921; 1925; 8.281568; CCTTG; 7.81641; 7.53757;
  
Sequence; GR-alpha [T00337]; 1948; 1952; 0.207689; AGAGG; 7.81641; 7.83842;
  
Sequence; GR-alpha [T00337]; 1953; 1957; 8.281568; CCTCC; 7.81641; 7.53757;
  
Sequence; GR-alpha [T00337]; 1957; 1961; 6.055408; CCTGA; 3.90820; 3.86993;
  
Sequence; GR-alpha [T00337]; 1974; 1978; 0.207689; AAAGG; 7.81641; 7.83842;
  
Sequence; GR-alpha [T00337]; 1986; 1990; 0.207689; AGAGG; 7.81641; 7.83842;
  
Sequence; GR-alpha [T00337]; 1992; 1996; 8.281568; GAAGG; 7.81641; 7.53757;
  
Sequence; AP-2alphaA [T00035]; 9; 14; 0.000000; GCAGGC; 0.97705; 0.90951;
  
Sequence; AP-2alphaA [T00035]; 198; 203; 0.000000; GCAGGC; 0.97705; 0.90951;
  
Sequence; AP-2alphaA [T00035]; 542; 547; 0.000000; GCAGGC; 0.97705; 0.90951;
  
Sequence; AP-2alphaA [T00035]; 638; 643; 3.970052; CAAGGC; 0.97705; 0.94224;
  
Sequence; AP-2alphaA [T00035]; 649; 654; 0.226186; CCAGGC; 0.97705; 0.90951;
  
Sequence; AP-2alphaA [T00035]; 653; 658; 1.871933; GCCTCC; 0.97705; 0.90645;
  
Sequence; AP-2alphaA [T00035]; 705; 710; 0.000000; GCAGGC; 0.97705; 0.90951;
  
Sequence; AP-2alphaA [T00035]; 735; 740; 3.743866; GAAGGC; 0.48853; 0.47274;
  
Sequence; AP-2alphaA [T00035]; 926; 931; 4.422424; TAAGGC; 0.97705; 0.94706;
  
Sequence; AP-2alphaA [T00035]; 989; 994; 1.871933; GCCTCC; 0.97705; 0.90645;
  
Sequence; AP-2alphaA [T00035]; 1094; 1099; 4.422424; TAAGGC; 0.97705; 0.94706;
  
Sequence; AP-2alphaA [T00035]; 1214; 1219; 2.550491; GCCTCA; 0.48853; 0.46599;
  
Sequence; AP-2alphaA [T00035]; 1369; 1374; 0.678558; GCCTGA; 0.48853; 0.46564;
  
Sequence; AP-2alphaA [T00035]; 1402; 1407; 0.226186; GCCTGG; 0.97705; 0.90951;
  
Sequence; AP-2alphaA [T00035]; 1426; 1431; 1.357116; GCCTGT; 0.48853; 0.47624;
  
Sequence; AP-2alphaA [T00035]; 1527; 1532; 4.422424; TAAGGC; 0.97705; 0.94706;
  
Sequence; AP-2alphaA [T00035]; 1552; 1557; 4.890408; GCCTAA; 0.97705; 0.98475;
  
Sequence; AP-2alphaA [T00035]; 1697; 1702; 3.229049; GCCTCT; 0.48853; 0.47274;
  
Sequence; AP-2alphaA [T00035]; 1948; 1953; 3.229049; AGAGGC; 0.48853; 0.47274;
  
Sequence; AP-2alphaA [T00035]; 1952; 1957; 1.871933; GCCTCC; 0.97705; 0.90645;
  
Sequence; PR B [T00696]; 16; 22; 9.743489; AACACAG; 1.09918; 1.18637;
  
Sequence; PR B [T00696]; 377; 383; 10.231719; AGGTGTT; 0.36639; 0.38342;
  
Sequence; PR B [T00696]; 486; 492; 1.404665; AACACTG; 0.36639; 0.38342;
  
Sequence; PR B [T00696]; 567; 573; 8.338824; CCCTGTT; 1.09918; 1.16864;
  
Sequence; PR B [T00696]; 587; 593; 8.338824; AACAGAC; 1.09918; 1.16864;
  
Sequence; PR B [T00696]; 670; 676; 1.404665; CAGTGTT; 0.36639; 0.38342;
  
Sequence; PR B [T00696]; 822; 828; 1.404665; AACACTC; 0.36639; 0.38342;
  
Sequence; PR B [T00696]; 918; 924; 1.404665; AACACTA; 0.36639; 0.38342;
  
Sequence; PR B [T00696]; 1015; 1021; 11.148154; CCTTGTT; 2.19836; 2.43885;
  
Sequence; PR B [T00696]; 1362; 1368; 11.636384; AACATGT; 0.73279; 0.79014;
  
Sequence; PR B [T00696]; 1408; 1414; 10.231719; AACACAT; 0.36639; 0.38342;
  
Sequence; PR B [T00696]; 1668; 1674; 9.743489; AACACGC; 1.09918; 1.18637;
  
Sequence; PR B [T00696]; 1711; 1717; 8.827054; AACAGCT; 0.36639; 0.37955;
  
Sequence; PR B [T00696]; 1714; 1720; 8.827054; AGCTGTT; 0.36639; 0.37955;
  
Sequence; PR B [T00696]; 1812; 1818; 11.148154; TCATGTT; 2.19836; 2.43885;
  
Sequence; PR A [T01661]; 16; 22; 9.743489; AACACAG; 1.09918; 1.18637;
  
Sequence; PR A [T01661]; 377; 383; 10.231719; AGGTGTT; 0.36639; 0.38342;
  
Sequence; PR A [T01661]; 486; 492; 1.404665; AACACTG; 0.36639; 0.38342;
  
Sequence; PR A [T01661]; 567; 573; 8.338824; CCCTGTT; 1.09918; 1.16864;
  
Sequence; PR A [T01661]; 587; 593; 8.338824; AACAGAC; 1.09918; 1.16864;
  
Sequence; PR A [T01661]; 670; 676; 1.404665; CAGTGTT; 0.36639; 0.38342;
  
Sequence; PR A [T01661]; 822; 828; 1.404665; AACACTC; 0.36639; 0.38342;
  
Sequence; PR A [T01661]; 918; 924; 1.404665; AACACTA; 0.36639; 0.38342;
  
Sequence; PR A [T01661]; 1015; 1021; 11.148154; CCTTGTT; 2.19836; 2.43885;
  
Sequence; PR A [T01661]; 1362; 1368; 11.636384; AACATGT; 0.73279; 0.79014;
  
Sequence; PR A [T01661]; 1408; 1414; 10.231719; AACACAT; 0.36639; 0.38342;
  
Sequence; PR A [T01661]; 1668; 1674; 9.743489; AACACGC; 1.09918; 1.18637;
  
Sequence; PR A [T01661]; 1711; 1717; 8.827054; AACAGCT; 0.36639; 0.37955;
  
Sequence; PR A [T01661]; 1714; 1720; 8.827054; AGCTGTT; 0.36639; 0.37955;
  
Sequence; PR A [T01661]; 1812; 1818; 11.148154; TCATGTT; 2.19836; 2.43885;
  
Sequence; Pax-5 [T00070]; 25; 31; 4.007279; ACAGCCC; 1.09918; 1.05070;
  
Sequence; Pax-5 [T00070]; 322; 328; 1.537547; GGGCGTG; 0.73279; 0.67075;
  
Sequence; Pax-5 [T00070]; 327; 333; 8.014558; TGGGCCC; 2.19836; 2.04146;
  
Sequence; Pax-5 [T00070]; 328; 334; 4.007279; GGGCCCT; 1.09918; 1.05070;
  
Sequence; Pax-5 [T00070]; 368; 374; 8.014558; TGGGCCC; 2.19836; 2.04146;
  
Sequence; Pax-5 [T00070]; 369; 375; 8.014558; GGGCCCC; 2.19836; 2.04146;
  
Sequence; Pax-5 [T00070]; 495; 501; 4.007279; AATGCCC; 1.09918; 1.05070;
  
Sequence; Pax-5 [T00070]; 563; 569; 5.544826; ATTGCCC; 0.73279; 0.69513;
  
Sequence; Pax-5 [T00070]; 611; 617; 9.552105; TTTGCCC; 1.46558; 1.36445;
  
Sequence; Pax-5 [T00070]; 634; 640; 9.552105; GGGCCAA; 1.46558; 1.36445;
  
Sequence; Pax-5 [T00070]; 644; 650; 4.007279; AGAGCCC; 1.09918; 1.05070;
  
Sequence; Pax-5 [T00070]; 711; 717; 4.007279; AAAGCCC; 1.09918; 1.05070;
  
Sequence; Pax-5 [T00070]; 794; 800; 4.007279; ACTGCCC; 1.09918; 1.05070;
  
Sequence; Pax-5 [T00070]; 1468; 1474; 8.014558; GGGCTGC; 2.19836; 2.04146;
  
Sequence; Pax-5 [T00070]; 1494; 1500; 9.552105; TTAGCCC; 1.46558; 1.36445;
  
Sequence; Pax-5 [T00070]; 1528; 1534; 4.007279; AAGGCCC; 1.09918; 1.05070;
  
Sequence; Pax-5 [T00070]; 1637; 1643; 5.544826; GGGCAAT; 0.73279; 0.69513;
  
Sequence; Pax-5 [T00070]; 1695; 1701; 8.014558; GGGCCTC; 2.19836; 2.04146;
  
Sequence; Pax-5 [T00070]; 1777; 1783; 9.552105; GTAGCCC; 1.46558; 1.36445;
  
Sequence; p53 [T00671]; 25; 31; 7.266844; ACAGCCC; 0.73279; 0.70048;
  
Sequence; p53 [T00671]; 322; 328; 3.375208; GGGCGTG; 0.73279; 0.68390;
  
Sequence; p53 [T00671]; 327; 333; 7.153797; TGGGCCC; 1.09918; 1.01757;
  
Sequence; p53 [T00671]; 328; 334; 8.912104; GGGCCCT; 0.12213; 0.11166;
  
Sequence; p53 [T00671]; 368; 374; 7.153797; TGGGCCC; 1.09918; 1.01757;
  
Sequence; p53 [T00671]; 369; 375; 8.208781; GGGCCCC; 0.48853; 0.44605;
  
Sequence; p53 [T00671]; 495; 501; 3.516613; AATGCCC; 0.73279; 0.68390;
  
Sequence; p53 [T00671]; 563; 569; 3.728319; ATTGCCC; 0.73279; 0.68390;
  
Sequence; p53 [T00671]; 611; 617; 1.970013; TTTGCCC; 0.36639; 0.35623;
  
Sequence; p53 [T00671]; 634; 640; 6.095267; GGGCCAA; 0.61066; 0.56234;
  
Sequence; p53 [T00671]; 644; 650; 8.537081; AGAGCCC; 0.12213; 0.11745;
  
Sequence; p53 [T00671]; 711; 717; 7.266844; AAAGCCC; 0.73279; 0.70048;
  
Sequence; p53 [T00671]; 794; 800; 3.516613; ACTGCCC; 0.73279; 0.68390;
  
Sequence; p53 [T00671]; 1468; 1474; 6.563521; GGGCTGC; 0.48853; 0.45222;
  
Sequence; p53 [T00671]; 1494; 1500; 5.720243; TTAGCCC; 0.61066; 0.57517;
  
Sequence; p53 [T00671]; 1528; 1534; 7.641867; AAGGCCC; 0.73279; 0.70048;
  
Sequence; p53 [T00671]; 1637; 1643; 3.728319; GGGCAAT; 0.73279; 0.68390;
  
Sequence; p53 [T00671]; 1695; 1701; 6.938545; GGGCCTC; 1.09918; 1.01757;
  
Sequence; p53 [T00671]; 1777; 1783; 6.775228; GTAGCCC; 1.09918; 1.01757;
  
Sequence; FOXP3 [T04280]; 33; 38; 4.756447; GAAAAC; 2.93115; 3.15504;
  
Sequence; FOXP3 [T04280]; 76; 81; 14.269340; GGAAAC; 5.86230; 6.08905;
  
Sequence; FOXP3 [T04280]; 118; 123; 9.512894; CAGAAC; 7.32788; 7.59854;
  
Sequence; FOXP3 [T04280]; 129; 134; 9.512894; CAGAAC; 7.32788; 7.59854;
  
Sequence; FOXP3 [T04280]; 169; 174; 9.512894; GTTCTG; 7.32788; 7.59854;
  
Sequence; FOXP3 [T04280]; 191; 196; 14.269340; GTTTCT; 5.86230; 6.08905;
  
Sequence; FOXP3 [T04280]; 228; 233; 14.269340; GTTTCT; 5.86230; 6.08905;
  
Sequence; FOXP3 [T04280]; 254; 259; 9.512894; GTTGGG; 7.32788; 7.59854;
  
Sequence; FOXP3 [T04280]; 381; 386; 14.269340; GTTTGC; 5.86230; 6.08905;
  
Sequence; FOXP3 [T04280]; 426; 431; 9.512894; CAGAAC; 7.32788; 7.59854;
  
Sequence; FOXP3 [T04280]; 446; 451; 0.000000; GTTGTC; 1.46558; 1.56979;
  
Sequence; FOXP3 [T04280]; 474; 479; 9.512894; AAGAAC; 7.32788; 7.59854;
  
Sequence; FOXP3 [T04280]; 483; 488; 9.512894; CAGAAC; 7.32788; 7.59854;
  
Sequence; FOXP3 [T04280]; 571; 576; 9.512894; GTTGGG; 7.32788; 7.59854;
  
Sequence; FOXP3 [T04280]; 584; 589; 4.756447; AAAAAC; 2.93115; 3.15504;
  
Sequence; FOXP3 [T04280]; 692; 697; 9.512894; CTAAAC; 7.32788; 7.59854;
  
Sequence; FOXP3 [T04280]; 819; 824; 9.512894; AGCAAC; 7.32788; 7.59854;
  
Sequence; FOXP3 [T04280]; 834; 839; 9.512894; CGCAAC; 7.32788; 7.59854;
  
Sequence; FOXP3 [T04280]; 839; 844; 9.512894; CCCAAC; 7.32788; 7.59854;
  
Sequence; FOXP3 [T04280]; 915; 920; 0.000000; GACAAC; 1.46558; 1.56979;
  
Sequence; FOXP3 [T04280]; 1019; 1024; 9.512894; GTTATT; 7.32788; 7.59854;
  
Sequence; FOXP3 [T04280]; 1024; 1029; 6.581441; TTCAAC; 0.97705; 1.03256;
  
Sequence; FOXP3 [T04280]; 1047; 1052; 11.337888; TTAAAC; 2.44263; 2.50026;
  
Sequence; FOXP3 [T04280]; 1076; 1081; 1.824994; TACAAC; 0.48853; 0.50968;
  
Sequence; FOXP3 [T04280]; 1086; 1091; 14.269340; AGAAAC; 5.86230; 6.08905;
  
Sequence; FOXP3 [T04280]; 1154; 1159; 14.269340; GGAAAC; 5.86230; 6.08905;
  
Sequence; FOXP3 [T04280]; 1170; 1175; 9.512894; GTTGGC; 7.32788; 7.59854;
  
Sequence; FOXP3 [T04280]; 1220; 1225; 14.269340; GTTTGC; 5.86230; 6.08905;
  
Sequence; FOXP3 [T04280]; 1332; 1337; 9.512894; GTTGGG; 7.32788; 7.59854;
  
Sequence; FOXP3 [T04280]; 1359; 1364; 14.269340; GGAAAC; 5.86230; 6.08905;
  
Sequence; FOXP3 [T04280]; 1521; 1526; 14.269340; GGAAAC; 5.86230; 6.08905;
  
Sequence; FOXP3 [T04280]; 1592; 1597; 9.512894; GTTGGG; 7.32788; 7.59854;
  
Sequence; FOXP3 [T04280]; 1679; 1684; 9.512894; GAGAAC; 7.32788; 7.59854;
  
Sequence; FOXP3 [T04280]; 1708; 1713; 9.512894; CAGAAC; 7.32788; 7.59854;
  
Sequence; FOXP3 [T04280]; 1718; 1723; 9.512894; GTTCTC; 7.32788; 7.59854;
  
Sequence; FOXP3 [T04280]; 1732; 1737; 4.756447; ATCAAC; 2.93115; 3.15504;
  
Sequence; FOXP3 [T04280]; 1810; 1815; 14.269340; GTTCAT; 5.86230; 6.08905;
  
Sequence; FOXP3 [T04280]; 1817; 1822; 6.581441; TTCAAC; 0.97705; 1.03256;
  
Sequence; FOXP3 [T04280]; 1852; 1857; 4.756447; GAAAAC; 2.93115; 3.15504;
  
Sequence; FOXP3 [T04280]; 1861; 1866; 9.512894; GTTTAT; 7.32788; 7.59854;
  
Sequence; FOXP3 [T04280]; 1916; 1921; 14.269340; GGAAAC; 5.86230; 6.08905;
  
Sequence; RXR-alpha [T01345]; 34; 40; 3.170788; AAAACCC; 0.24426; 0.25557;
  
Sequence; RXR-alpha [T01345]; 77; 83; 4.019014; GAAACCC; 0.97705; 0.95062;
  
Sequence; RXR-alpha [T01345]; 130; 136; 2.544678; AGAACCC; 0.85492; 0.83658;
  
Sequence; RXR-alpha [T01345]; 278; 284; 1.696452; CTGACCC; 0.48853; 0.47294;
  
Sequence; RXR-alpha [T01345]; 318; 324; 5.271235; GGGTGGG; 0.61066; 0.59594;
  
Sequence; RXR-alpha [T01345]; 384; 390; 2.544678; TGCACCC; 0.85492; 0.83658;
  
Sequence; RXR-alpha [T01345]; 509; 515; 0.848226; GGGTCCA; 0.48853; 0.47078;
  
Sequence; RXR-alpha [T01345]; 835; 841; 5.271235; GCAACCC; 0.61066; 0.59594;
  
Sequence; RXR-alpha [T01345]; 1034; 1040; 4.423008; TCCACCC; 0.24426; 0.24206;
  
Sequence; RXR-alpha [T01345]; 1148; 1154; 3.392904; GGGTGAG; 1.09918; 1.05447;
  
Sequence; RXR-alpha [T01345]; 1181; 1187; 3.392904; TGTACCC; 1.09918; 1.05447;
  
Sequence; RXR-alpha [T01345]; 1249; 1255; 3.392904; GGGTCAT; 1.09918; 1.05447;
  
Sequence; RXR-alpha [T01345]; 1309; 1315; 4.241130; CTTACCC; 0.97705; 0.95062;
  
Sequence; RXR-alpha [T01345]; 1336; 1342; 5.271235; GGGTGGG; 0.61066; 0.59594;
  
Sequence; RXR-alpha [T01345]; 1392; 1398; 3.392904; GGGTAAA; 1.09918; 1.05447;
  
Sequence; RXR-alpha [T01345]; 1418; 1424; 4.241130; GTGACCC; 0.97705; 0.95062;
  
Sequence; RXR-alpha [T01345]; 1498; 1504; 5.271235; CCCACCC; 0.61066; 0.59594;
  
Sequence; RXR-alpha [T01345]; 1577; 1583; 6.563693; GGGTATT; 0.24426; 0.24436;
  
Sequence; RXR-alpha [T01345]; 1598; 1604; 4.241130; GGGACCC; 0.97705; 0.95062;
  
Sequence; RXR-alpha [T01345]; 1680; 1686; 2.544678; AGAACCC; 0.85492; 0.83658;
  
Sequence; RAR-beta [T00721]; 32; 41; 7.478240; AGAAAACCCA; 0.24426; 0.24603;
  
Sequence; RAR-beta [T00721]; 75; 84; 6.396730; GGGAAACCCA; 0.18320; 0.18436;
  
Sequence; RAR-beta [T00721]; 128; 137; 6.396730; ACAGAACCCT; 0.18320; 0.18436;
  
Sequence; RAR-beta [T00721]; 833; 842; 12.811926; CCGCAACCCA; 0.12976; 0.13145;
  
Sequence; RAR-beta [T00721]; 1678; 1687; 8.559750; TGAGAACCCA; 0.26716; 0.27138;
  
Sequence; IRF-1 [T00423]; 72; 80; 3.352297; AAGGGGAAA; 0.06870; 0.07634;
  
Sequence; IRF-1 [T00423]; 488; 496; 6.116243; CACTGGAAA; 0.16793; 0.17682;
  
Sequence; IRF-1 [T00423]; 764; 772; 5.659770; TTTCCAGAT; 0.22900; 0.24108;
  
Sequence; IRF-1 [T00423]; 961; 969; 4.626471; AAGAGGAAA; 0.05343; 0.05720;
  
Sequence; IRF-1 [T00423]; 1012; 1020; 2.890712; TTTCCTTGT; 0.07633; 0.08621;
  
Sequence; IRF-1 [T00423]; 1150; 1158; 8.661524; GTGAGGAAA; 0.20610; 0.20682;
  
Sequence; IRF-1 [T00423]; 1355; 1363; 6.497844; AGGTGGAAA; 0.19083; 0.19486;
  
Sequence; IRF-1 [T00423]; 1448; 1456; 7.732782; CTCTGGAAA; 0.14503; 0.14450;
  
Sequence; IRF-1 [T00423]; 1517; 1525; 7.387351; TTGGGGAAA; 0.14503; 0.14450;
  
Sequence; IRF-1 [T00423]; 1724; 1732; 7.477948; CCCAGGAAA; 0.14503; 0.14450;
  
Sequence; IRF-1 [T00423]; 1912; 1920; 6.549276; GCCGGGAAA; 0.19083; 0.19486;
  
Sequence; NF-AT1 [T00550]; 76; 84; 10.170774; GGAAACCCA; 0.12213; 0.12280;
  
Sequence; NF-AT1 [T00550]; 492; 500; 10.546788; GGAAATGCC; 0.11450; 0.11475;
  
Sequence; NF-AT1 [T00550]; 760; 768; 3.098758; AGACTTTCC; 0.03053; 0.03033;
  
Sequence; NF-AT1 [T00550]; 965; 973; 7.744746; GGAAAGTAA; 0.19846; 0.20990;
  
Sequence; NF-AT1 [T00550]; 1008; 1016; 7.095752; TTCTTTTCC; 0.15266; 0.16675;
  
Sequence; NF-AT1 [T00550]; 1154; 1162; 9.691726; GGAAACCAA; 0.16793; 0.17266;
  
Sequence; NF-AT1 [T00550]; 1359; 1367; 7.721010; GGAAACATG; 0.19846; 0.20990;
  
Sequence; NF-AT1 [T00550]; 1452; 1460; 7.744746; GGAAAGTAA; 0.19846; 0.20990;
  
Sequence; NF-AT1 [T00550]; 1521; 1529; 9.691726; GGAAACTAA; 0.16793; 0.17266;
  
Sequence; NF-AT1 [T00550]; 1728; 1736; 9.042733; GGAAATCAA; 0.22900; 0.23296;
  
Sequence; NF-AT1 [T00550]; 1916; 1924; 4.566689; GGAAACCTT; 0.06870; 0.06945;
  
Sequence; TFII-I [T00824]; 53; 58; 9.512894; GGAAGG; 7.32788; 7.26287;
  
Sequence; TFII-I [T00824]; 57; 62; 11.337888; GGAGAA; 2.44263; 2.35967;
  
Sequence; TFII-I [T00824]; 76; 81; 9.512894; GGAAAC; 7.32788; 7.26287;
  
Sequence; TFII-I [T00824]; 84; 89; 14.269340; AGGTCC; 5.86230; 5.77036;
  
Sequence; TFII-I [T00824]; 212; 217; 14.269340; ACTTCC; 5.86230; 5.77036;
  
Sequence; TFII-I [T00824]; 241; 246; 6.581441; CACTCC; 0.97705; 0.94719;
  
Sequence; TFII-I [T00824]; 271; 276; 4.756447; CAGTCC; 2.93115; 2.90946;
  
Sequence; TFII-I [T00824]; 307; 312; 4.756447; GGACAT; 2.93115; 2.90946;
  
Sequence; TFII-I [T00824]; 309; 314; 14.269340; ACATCC; 5.86230; 5.77036;
  
Sequence; TFII-I [T00824]; 340; 345; 11.337888; CCCTCC; 2.44263; 2.35967;
  
Sequence; TFII-I [T00824]; 344; 349; 11.337888; CCCTCC; 2.44263; 2.35967;
  
Sequence; TFII-I [T00824]; 443; 448; 11.337888; GGAGTT; 2.44263; 2.35967;
  
Sequence; TFII-I [T00824]; 447; 452; 9.512894; TTGTCC; 7.32788; 7.26287;
  
Sequence; TFII-I [T00824]; 492; 497; 4.756447; GGAAAT; 2.93115; 2.90946;
  
Sequence; TFII-I [T00824]; 499; 504; 11.337888; CCCTCC; 2.44263; 2.35967;
  
Sequence; TFII-I [T00824]; 552; 557; 0.000000; GGACAG; 1.46558; 1.44897;
  
Sequence; TFII-I [T00824]; 575; 580; 0.000000; GGACAG; 1.46558; 1.44897;
  
Sequence; TFII-I [T00824]; 699; 704; 4.756447; GGAATG; 2.93115; 2.90946;
  
Sequence; TFII-I [T00824]; 722; 727; 9.512894; GGAAGG; 7.32788; 7.26287;
  
Sequence; TFII-I [T00824]; 728; 733; 9.512894; GGAAGG; 7.32788; 7.26287;
  
Sequence; TFII-I [T00824]; 763; 768; 0.000000; CTTTCC; 1.46558; 1.44897;
  
Sequence; TFII-I [T00824]; 781; 786; 9.512894; AAGTCC; 7.32788; 7.26287;
  
Sequence; TFII-I [T00824]; 801; 806; 9.512894; CGGTCC; 7.32788; 7.26287;
  
Sequence; TFII-I [T00824]; 805; 810; 9.512894; CCTTCC; 7.32788; 7.26287;
  
Sequence; TFII-I [T00824]; 824; 829; 6.581441; CACTCC; 0.97705; 0.94719;
  
Sequence; TFII-I [T00824]; 907; 912; 9.512894; AAATCC; 7.32788; 7.26287;
  
Sequence; TFII-I [T00824]; 939; 944; 9.512894; GGAAGG; 7.32788; 7.26287;
  
Sequence; TFII-I [T00824]; 945; 950; 14.269340; GGAAGT; 5.86230; 5.77036;
  
Sequence; TFII-I [T00824]; 953; 958; 0.000000; GGATAG; 1.46558; 1.44897;
  
Sequence; TFII-I [T00824]; 965; 970; 0.000000; GGAAAG; 1.46558; 1.44897;
  
Sequence; TFII-I [T00824]; 975; 980; 14.269340; GGACCT; 5.86230; 5.77036;
  
Sequence; TFII-I [T00824]; 982; 987; 0.000000; CTATCC; 1.46558; 1.44897;
  
Sequence; TFII-I [T00824]; 997; 1002; 14.269340; GGAATC; 5.86230; 5.77036;
  
Sequence; TFII-I [T00824]; 998; 1003; 14.269340; GAATCC; 5.86230; 5.77036;
  
Sequence; TFII-I [T00824]; 1011; 1016; 9.512894; TTTTCC; 7.32788; 7.26287;
  
Sequence; TFII-I [T00824]; 1105; 1110; 14.269340; TAATCC; 5.86230; 5.77036;
  
Sequence; TFII-I [T00824]; 1131; 1136; 6.581441; GGAGAT; 0.97705; 0.94719;
  
Sequence; TFII-I [T00824]; 1154; 1159; 9.512894; GGAAAC; 7.32788; 7.26287;
  
Sequence; TFII-I [T00824]; 1316; 1321; 14.269340; ACTTCC; 5.86230; 5.77036;
  
Sequence; TFII-I [T00824]; 1343; 1348; 0.000000; GGACAG; 1.46558; 1.44897;
  
Sequence; TFII-I [T00824]; 1359; 1364; 9.512894; GGAAAC; 7.32788; 7.26287;
  
Sequence; TFII-I [T00824]; 1375; 1380; 14.269340; GAATCC; 5.86230; 5.77036;
  
Sequence; TFII-I [T00824]; 1411; 1416; 14.269340; ACATCC; 5.86230; 5.77036;
  
Sequence; TFII-I [T00824]; 1452; 1457; 0.000000; GGAAAG; 1.46558; 1.44897;
  
Sequence; TFII-I [T00824]; 1464; 1469; 9.512894; GGAAGG; 7.32788; 7.26287;
  
Sequence; TFII-I [T00824]; 1478; 1483; 14.269340; GGAACT; 5.86230; 5.77036;
  
Sequence; TFII-I [T00824]; 1488; 1493; 4.756447; GGATAT; 2.93115; 2.90946;
  
Sequence; TFII-I [T00824]; 1521; 1526; 9.512894; GGAAAC; 7.32788; 7.26287;
  
Sequence; TFII-I [T00824]; 1566; 1571; 11.337888; GGAGAA; 2.44263; 2.35967;
  
Sequence; TFII-I [T00824]; 1615; 1620; 4.756447; CAATCC; 2.93115; 2.90946;
  
Sequence; TFII-I [T00824]; 1632; 1637; 4.756447; GGAATG; 2.93115; 2.90946;
  
Sequence; TFII-I [T00824]; 1644; 1649; 9.512894; TTGTCC; 7.32788; 7.26287;
  
Sequence; TFII-I [T00824]; 1719; 1724; 11.337888; TTCTCC; 2.44263; 2.35967;
  
Sequence; TFII-I [T00824]; 1728; 1733; 4.756447; GGAAAT; 2.93115; 2.90946;
  
Sequence; TFII-I [T00824]; 1782; 1787; 9.512894; CCATCC; 7.32788; 7.26287;
  
Sequence; TFII-I [T00824]; 1903; 1908; 9.512894; AAATCC; 7.32788; 7.26287;
  
Sequence; TFII-I [T00824]; 1916; 1921; 9.512894; GGAAAC; 7.32788; 7.26287;
  
Sequence; TFII-I [T00824]; 1980; 1985; 0.000000; GGACAG; 1.46558; 1.44897;
  
Sequence; STAT4 [T01577]; 53; 58; 5.882353; GGAAGG; 0.48853; 0.46994;
  
Sequence; STAT4 [T01577]; 76; 81; 1.470588; GGAAAC; 1.95410; 2.00857;
  
Sequence; STAT4 [T01577]; 106; 111; 4.411765; GCTTCC; 1.95410; 1.92171;
  
Sequence; STAT4 [T01577]; 212; 217; 2.941176; ACTTCC; 2.93115; 2.93673;
  
Sequence; STAT4 [T01577]; 258; 263; 4.411765; GGAAGA; 1.95410; 1.92171;
  
Sequence; STAT4 [T01577]; 492; 497; 0.000000; GGAAAT; 0.48853; 0.50148;
  
Sequence; STAT4 [T01577]; 673; 678; 2.941176; TGTTCC; 2.93115; 2.93673;
  
Sequence; STAT4 [T01577]; 699; 704; 4.411765; GGAATG; 1.95410; 1.92171;
  
Sequence; STAT4 [T01577]; 722; 727; 5.882353; GGAAGG; 0.48853; 0.46994;
  
Sequence; STAT4 [T01577]; 728; 733; 5.882353; GGAAGG; 0.48853; 0.46994;
  
Sequence; STAT4 [T01577]; 763; 768; 2.941176; CTTTCC; 2.93115; 2.93673;
  
Sequence; STAT4 [T01577]; 805; 810; 5.882353; CCTTCC; 0.48853; 0.46994;
  
Sequence; STAT4 [T01577]; 939; 944; 5.882353; GGAAGG; 0.48853; 0.46994;
  
Sequence; STAT4 [T01577]; 945; 950; 2.941176; GGAAGT; 2.93115; 2.93673;
  
Sequence; STAT4 [T01577]; 965; 970; 2.941176; GGAAAG; 2.93115; 2.93673;
  
Sequence; STAT4 [T01577]; 997; 1002; 2.941176; GGAATC; 2.93115; 2.93673;
  
Sequence; STAT4 [T01577]; 1011; 1016; 1.470588; TTTTCC; 1.95410; 2.00857;
  
Sequence; STAT4 [T01577]; 1031; 1036; 4.411765; TCTTCC; 1.95410; 1.92171;
  
Sequence; STAT4 [T01577]; 1154; 1159; 1.470588; GGAAAC; 1.95410; 2.00857;
  
Sequence; STAT4 [T01577]; 1316; 1321; 2.941176; ACTTCC; 2.93115; 2.93673;
  
Sequence; STAT4 [T01577]; 1359; 1364; 1.470588; GGAAAC; 1.95410; 2.00857;
  
Sequence; STAT4 [T01577]; 1406; 1411; 2.941176; GGAACA; 2.93115; 2.93673;
  
Sequence; STAT4 [T01577]; 1438; 1443; 4.411765; GGAAGC; 1.95410; 1.92171;
  
Sequence; STAT4 [T01577]; 1452; 1457; 2.941176; GGAAAG; 2.93115; 2.93673;
  
Sequence; STAT4 [T01577]; 1464; 1469; 5.882353; GGAAGG; 0.48853; 0.46994;
  
Sequence; STAT4 [T01577]; 1478; 1483; 1.470588; GGAACT; 1.95410; 2.00857;
  
Sequence; STAT4 [T01577]; 1521; 1526; 1.470588; GGAAAC; 1.95410; 2.00857;
  
Sequence; STAT4 [T01577]; 1632; 1637; 4.411765; GGAATG; 1.95410; 1.92171;
  
Sequence; STAT4 [T01577]; 1666; 1671; 2.941176; GGAACA; 2.93115; 2.93673;
  
Sequence; STAT4 [T01577]; 1728; 1733; 0.000000; GGAAAT; 0.48853; 0.50148;
  
Sequence; STAT4 [T01577]; 1916; 1921; 1.470588; GGAAAC; 1.95410; 2.00857;
  
Sequence; c-Ets-1 [T00112]; 51; 57; 5.558311; GGGGAAG; 0.36639; 0.35211;
  
Sequence; c-Ets-1 [T00112]; 74; 80; 6.943262; GGGGAAA; 0.73279; 0.73837;
  
Sequence; c-Ets-1 [T00112]; 107; 113; 0.000000; CTTCCTG; 0.24426; 0.24370;
  
Sequence; c-Ets-1 [T00112]; 213; 219; 0.000000; CTTCCTG; 0.24426; 0.24370;
  
Sequence; c-Ets-1 [T00112]; 256; 262; 5.814485; TGGGAAG; 0.36639; 0.36418;
  
Sequence; c-Ets-1 [T00112]; 490; 496; 6.039428; CTGGAAA; 0.36639; 0.36418;
  
Sequence; c-Ets-1 [T00112]; 674; 680; 3.590463; GTTCCTC; 0.61066; 0.61827;
  
Sequence; c-Ets-1 [T00112]; 697; 703; 8.809329; CTGGAAT; 0.85492; 0.84901;
  
Sequence; c-Ets-1 [T00112]; 720; 726; 4.782565; GTGGAAG; 0.48853; 0.48569;
  
Sequence; c-Ets-1 [T00112]; 726; 732; 5.558311; GGGGAAG; 0.36639; 0.35211;
  
Sequence; c-Ets-1 [T00112]; 764; 770; 6.039428; TTTCCAG; 0.36639; 0.36418;
  
Sequence; c-Ets-1 [T00112]; 806; 812; 5.558311; CTTCCCC; 0.36639; 0.35211;
  
Sequence; c-Ets-1 [T00112]; 937; 943; 5.814485; TGGGAAG; 0.36639; 0.36418;
  
Sequence; c-Ets-1 [T00112]; 943; 949; 5.558311; GGGGAAG; 0.36639; 0.35211;
  
Sequence; c-Ets-1 [T00112]; 963; 969; 1.513038; GAGGAAA; 0.36639; 0.40157;
  
Sequence; c-Ets-1 [T00112]; 995; 1001; 9.065503; ATGGAAT; 0.85492; 0.84901;
  
Sequence; c-Ets-1 [T00112]; 1012; 1018; 1.641124; TTTCCTT; 0.36639; 0.40157;
  
Sequence; c-Ets-1 [T00112]; 1032; 1038; 4.782565; CTTCCAC; 0.48853; 0.48569;
  
Sequence; c-Ets-1 [T00112]; 1152; 1158; 1.513038; GAGGAAA; 0.36639; 0.40157;
  
Sequence; c-Ets-1 [T00112]; 1317; 1323; 5.558311; CTTCCCC; 0.36639; 0.35211;
  
Sequence; c-Ets-1 [T00112]; 1357; 1363; 6.167515; GTGGAAA; 0.36639; 0.36418;
  
Sequence; c-Ets-1 [T00112]; 1404; 1410; 8.116854; CTGGAAC; 0.24426; 0.23650;
  
Sequence; c-Ets-1 [T00112]; 1436; 1442; 0.384261; TAGGAAG; 0.24426; 0.25238;
  
Sequence; c-Ets-1 [T00112]; 1450; 1456; 6.039428; CTGGAAA; 0.36639; 0.36418;
  
Sequence; c-Ets-1 [T00112]; 1462; 1468; 4.910652; ATGGAAG; 0.48853; 0.48569;
  
Sequence; c-Ets-1 [T00112]; 1476; 1482; 9.276861; TGGGAAC; 0.36639; 0.35393;
  
Sequence; c-Ets-1 [T00112]; 1519; 1525; 6.943262; GGGGAAA; 0.73279; 0.73837;
  
Sequence; c-Ets-1 [T00112]; 1630; 1636; 8.809329; CTGGAAT; 0.85492; 0.84901;
  
Sequence; c-Ets-1 [T00112]; 1664; 1670; 3.846637; TAGGAAC; 0.24426; 0.24718;
  
Sequence; c-Ets-1 [T00112]; 1726; 1732; 1.384951; CAGGAAA; 0.36639; 0.40157;
  
Sequence; c-Ets-1 [T00112]; 1914; 1920; 6.815175; CGGGAAA; 0.73279; 0.73837;
  
Sequence; Elk-1 [T00250]; 49; 57; 8.931691; AGGGGGAAG; 0.24426; 0.23183;
  
Sequence; Elk-1 [T00250]; 107; 115; 2.164966; CTTCCTGGC; 0.05343; 0.05293;
  
Sequence; Elk-1 [T00250]; 213; 221; 3.381796; CTTCCTGAT; 0.04580; 0.04554;
  
Sequence; Elk-1 [T00250]; 254; 262; 13.690145; GTTGGGAAG; 0.01527; 0.01501;
  
Sequence; Elk-1 [T00250]; 718; 726; 12.179139; CTGTGGAAG; 0.24426; 0.24110;
  
Sequence; Elk-1 [T00250]; 724; 732; 11.096657; AAGGGGAAG; 0.26716; 0.25756;
  
Sequence; Elk-1 [T00250]; 806; 814; 12.179139; CTTCCCCAG; 0.24426; 0.24110;
  
Sequence; Elk-1 [T00250]; 935; 943; 13.824493; TTTGGGAAG; 0.04580; 0.04597;
  
Sequence; Elk-1 [T00250]; 941; 949; 11.096657; AAGGGGAAG; 0.26716; 0.25756;
  
Sequence; Elk-1 [T00250]; 1032; 1040; 8.797343; CTTCCACCC; 0.24426; 0.23183;
  
Sequence; Elk-1 [T00250]; 1317; 1325; 8.931691; CTTCCCCCA; 0.24426; 0.23183;
  
Sequence; Elk-1 [T00250]; 1434; 1442; 1.779702; TGTAGGAAG; 0.05343; 0.05293;
  
Sequence; Elk-1 [T00250]; 1460; 1468; 11.919333; AAATGGAAG; 0.24426; 0.24110;
  
Sequence; ELF-1 [T01113]; 104; 116; 14.267792; CTGCTTCCTGGCA; 0.04914; 0.04883;
  
Sequence; ELF-1 [T01113]; 210; 222; 8.385761; TCACTTCCTGATT; 0.00668; 0.00665;
  
Sequence; ENKTF-1 [T00255]; 112; 119; 13.885529; TGGCAGCA; 1.09918; 1.04923;
  
Sequence; ENKTF-1 [T00255]; 137; 144; 11.374018; TTAAGCCA; 1.09918; 1.09754;
  
Sequence; ENKTF-1 [T00255]; 154; 161; 13.885529; TGGCAGGA; 1.09918; 1.04923;
  
Sequence; ENKTF-1 [T00255]; 364; 371; 8.198520; TGGCTGGG; 0.73279; 0.68458;
  
Sequence; ENKTF-1 [T00255]; 433; 440; 13.885529; TGCTGCCA; 1.09918; 1.04923;
  
Sequence; ENKTF-1 [T00255]; 516; 523; 13.885529; TGGCTACT; 1.09918; 1.04923;
  
Sequence; ENKTF-1 [T00255]; 540; 547; 13.885529; TGGCAGGC; 1.09918; 1.04923;
  
Sequence; ENKTF-1 [T00255]; 629; 636; 8.198520; TGGCAGGG; 0.73279; 0.68458;
  
Sequence; ENKTF-1 [T00255]; 632; 639; 5.687009; CAGGGCCA; 0.73279; 0.71771;
  
Sequence; ENKTF-1 [T00255]; 703; 710; 13.885529; TGGCAGGC; 1.09918; 1.04923;
  
Sequence; ENKTF-1 [T00255]; 845; 852; 12.629773; TGGCTCCC; 2.19836; 2.14640;
  
Sequence; ENKTF-1 [T00255]; 1060; 1067; 13.885529; TCCAGCCA; 1.09918; 1.04923;
  
Sequence; ENKTF-1 [T00255]; 1094; 1101; 11.374018; TAAGGCCA; 1.09918; 1.09754;
  
Sequence; ENKTF-1 [T00255]; 1172; 1179; 5.687009; TGGCACTG; 0.73279; 0.71771;
  
Sequence; ENKTF-1 [T00255]; 1438; 1445; 12.629773; GGAAGCCA; 2.19836; 2.14640;
  
Sequence; ENKTF-1 [T00255]; 1930; 1937; 11.374018; TGGCTTTT; 1.09918; 1.09754;
  
Sequence; c-Myb [T00137]; 120; 127; 14.459319; GAACTCAG; 0.36639; 0.38046;
  
Sequence; c-Myb [T00137]; 165; 172; 7.442719; GTAAGTTC; 0.42746; 0.43248;
  
Sequence; c-Myb [T00137]; 172; 179; 14.459319; CTGAGTTA; 0.36639; 0.38046;
  
Sequence; c-Myb [T00137]; 442; 449; 11.694333; TGGAGTTG; 0.42746; 0.43884;
  
Sequence; c-Myb [T00137]; 476; 483; 8.872587; GAACTAGC; 0.39693; 0.40120;
  
Sequence; c-Myb [T00137]; 680; 687; 5.137438; CCCAGTTA; 0.30533; 0.30190;
  
Sequence; c-Myb [T00137]; 694; 701; 8.412632; AAACTGGA; 0.30533; 0.31395;
  
Sequence; c-Myb [T00137]; 841; 848; 0.867346; CAACTGGC; 0.03053; 0.02915;
  
Sequence; c-Myb [T00137]; 1026; 1033; 7.662426; CAACTTCT; 0.42746; 0.43248;
  
Sequence; c-Myb [T00137]; 1049; 1056; 13.702607; AAACTTTA; 0.12213; 0.13272;
  
Sequence; c-Myb [T00137]; 1166; 1173; 6.454077; CTCAGTTG; 0.30533; 0.30286;
  
Sequence; c-Myb [T00137]; 1216; 1223; 9.024874; CTCAGTTT; 0.39693; 0.40120;
  
Sequence; c-Myb [T00137]; 1328; 1335; 6.454077; CACAGTTG; 0.30533; 0.30286;
  
Sequence; c-Myb [T00137]; 1479; 1486; 10.427413; GAACTTTG; 0.18320; 0.18527;
  
Sequence; c-Myb [T00137]; 1554; 1561; 10.427413; CTAAGTTA; 0.18320; 0.18527;
  
Sequence; c-Myb [T00137]; 1734; 1741; 11.131810; CAACTTTT; 0.42746; 0.44298;
  
Sequence; c-Myb [T00137]; 1806; 1813; 8.947824; AGAAGTTC; 0.39693; 0.40120;
  
Sequence; c-Myb [T00137]; 1857; 1864; 11.712811; CTAAGTTT; 0.42746; 0.43884;
  
Sequence; YY1 [T00915]; 183; 186; 0.000000; CCAT; 7.81641; 7.72659;
  
Sequence; YY1 [T00915]; 289; 292; 0.000000; CCAT; 7.81641; 7.72659;
  
Sequence; YY1 [T00915]; 452; 455; 0.000000; CCAT; 7.81641; 7.72659;
  
Sequence; YY1 [T00915]; 503; 506; 0.000000; CCAT; 7.81641; 7.72659;
  
Sequence; YY1 [T00915]; 513; 516; 0.000000; CCAT; 7.81641; 7.72659;
  
Sequence; YY1 [T00915]; 515; 518; 0.000000; ATGG; 7.81641; 7.72659;
  
Sequence; YY1 [T00915]; 702; 705; 0.000000; ATGG; 7.81641; 7.72659;
  
Sequence; YY1 [T00915]; 993; 996; 0.000000; CCAT; 7.81641; 7.72659;
  
Sequence; YY1 [T00915]; 995; 998; 0.000000; ATGG; 7.81641; 7.72659;
  
Sequence; YY1 [T00915]; 1040; 1043; 0.000000; CCAT; 7.81641; 7.72659;
  
Sequence; YY1 [T00915]; 1135; 1138; 0.000000; ATGG; 7.81641; 7.72659;
  
Sequence; YY1 [T00915]; 1254; 1257; 0.000000; ATGG; 7.81641; 7.72659;
  
Sequence; YY1 [T00915]; 1462; 1465; 0.000000; ATGG; 7.81641; 7.72659;
  
Sequence; YY1 [T00915]; 1620; 1623; 0.000000; CCAT; 7.81641; 7.72659;
  
Sequence; YY1 [T00915]; 1635; 1638; 0.000000; ATGG; 7.81641; 7.72659;
  
Sequence; YY1 [T00915]; 1782; 1785; 0.000000; CCAT; 7.81641; 7.72659;
  
Sequence; PU.1 [T02068]; 210; 222; 6.520619; TCACTTCCTGATT; 0.00417; 0.00406;
  
Sequence; PU.1 [T02068]; 1314; 1326; 14.151155; CCACTTCCCCCAA; 0.03602; 0.03533;
  
Sequence; C/EBPbeta [T00581]; 67; 70; 0.000000; GCAA; 15.63281; 16.30946;
  
Sequence; C/EBPbeta [T00581]; 237; 240; 1.366559; TTGA; 15.63281; 15.98168;
  
Sequence; C/EBPbeta [T00581]; 255; 258; 1.639871; TTGG; 15.63281; 15.98168;
  
Sequence; C/EBPbeta [T00581]; 374; 377; 1.639871; CCAA; 15.63281; 15.98168;
  
Sequence; C/EBPbeta [T00581]; 383; 386; 0.000000; TTGC; 15.63281; 16.30946;
  
Sequence; C/EBPbeta [T00581]; 392; 395; 0.000000; TTGC; 15.63281; 16.30946;
  
Sequence; C/EBPbeta [T00581]; 419; 422; 0.000000; GCAA; 15.63281; 16.30946;
  
Sequence; C/EBPbeta [T00581]; 447; 450; 0.000000; TTGT; 15.63281; 16.30946;
  
Sequence; C/EBPbeta [T00581]; 455; 458; 0.000000; TTGC; 15.63281; 16.30946;
  
Sequence; C/EBPbeta [T00581]; 529; 532; 1.366559; TCAA; 15.63281; 15.98168;
  
Sequence; C/EBPbeta [T00581]; 564; 567; 0.000000; TTGC; 15.63281; 16.30946;
  
Sequence; C/EBPbeta [T00581]; 572; 575; 1.639871; TTGG; 15.63281; 15.98168;
  
Sequence; C/EBPbeta [T00581]; 612; 615; 0.000000; TTGC; 15.63281; 16.30946;
  
Sequence; C/EBPbeta [T00581]; 637; 640; 1.639871; CCAA; 15.63281; 15.98168;
  
Sequence; C/EBPbeta [T00581]; 709; 712; 0.000000; GCAA; 15.63281; 16.30946;
  
Sequence; C/EBPbeta [T00581]; 741; 744; 1.366559; TCAA; 15.63281; 15.98168;
  
Sequence; C/EBPbeta [T00581]; 745; 748; 1.366559; TCAA; 15.63281; 15.98168;
  
Sequence; C/EBPbeta [T00581]; 820; 823; 0.000000; GCAA; 15.63281; 16.30946;
  
Sequence; C/EBPbeta [T00581]; 835; 838; 0.000000; GCAA; 15.63281; 16.30946;
  
Sequence; C/EBPbeta [T00581]; 840; 843; 1.639871; CCAA; 15.63281; 15.98168;
  
Sequence; C/EBPbeta [T00581]; 905; 908; 0.000000; ACAA; 15.63281; 16.30946;
  
Sequence; C/EBPbeta [T00581]; 911; 914; 1.639871; CCAA; 15.63281; 15.98168;
  
Sequence; C/EBPbeta [T00581]; 916; 919; 0.000000; ACAA; 15.63281; 16.30946;
  
Sequence; C/EBPbeta [T00581]; 936; 939; 1.639871; TTGG; 15.63281; 15.98168;
  
Sequence; C/EBPbeta [T00581]; 1017; 1020; 0.000000; TTGT; 15.63281; 16.30946;
  
Sequence; C/EBPbeta [T00581]; 1025; 1028; 1.366559; TCAA; 15.63281; 15.98168;
  
Sequence; C/EBPbeta [T00581]; 1077; 1080; 0.000000; ACAA; 15.63281; 16.30946;
  
Sequence; C/EBPbeta [T00581]; 1099; 1102; 1.639871; CCAA; 15.63281; 15.98168;
  
Sequence; C/EBPbeta [T00581]; 1109; 1112; 1.639871; CCAA; 15.63281; 15.98168;
  
Sequence; C/EBPbeta [T00581]; 1159; 1162; 1.639871; CCAA; 15.63281; 15.98168;
  
Sequence; C/EBPbeta [T00581]; 1171; 1174; 1.639871; TTGG; 15.63281; 15.98168;
  
Sequence; C/EBPbeta [T00581]; 1188; 1191; 0.000000; TTGT; 15.63281; 16.30946;
  
Sequence; C/EBPbeta [T00581]; 1222; 1225; 0.000000; TTGC; 15.63281; 16.30946;
  
Sequence; C/EBPbeta [T00581]; 1293; 1296; 0.000000; GCAA; 15.63281; 16.30946;
  
Sequence; C/EBPbeta [T00581]; 1323; 1326; 1.639871; CCAA; 15.63281; 15.98168;
  
Sequence; C/EBPbeta [T00581]; 1333; 1336; 1.639871; TTGG; 15.63281; 15.98168;
  
Sequence; C/EBPbeta [T00581]; 1475; 1478; 1.639871; TTGG; 15.63281; 15.98168;
  
Sequence; C/EBPbeta [T00581]; 1484; 1487; 1.366559; TTGA; 15.63281; 15.98168;
  
Sequence; C/EBPbeta [T00581]; 1517; 1520; 1.639871; TTGG; 15.63281; 15.98168;
  
Sequence; C/EBPbeta [T00581]; 1562; 1565; 0.000000; GCAA; 15.63281; 16.30946;
  
Sequence; C/EBPbeta [T00581]; 1575; 1578; 1.639871; TTGG; 15.63281; 15.98168;
  
Sequence; C/EBPbeta [T00581]; 1593; 1596; 1.639871; TTGG; 15.63281; 15.98168;
  
Sequence; C/EBPbeta [T00581]; 1603; 1606; 1.639871; CCAA; 15.63281; 15.98168;
  
Sequence; C/EBPbeta [T00581]; 1614; 1617; 1.366559; TCAA; 15.63281; 15.98168;
  
Sequence; C/EBPbeta [T00581]; 1623; 1626; 0.000000; TTGT; 15.63281; 16.30946;
  
Sequence; C/EBPbeta [T00581]; 1639; 1642; 0.000000; GCAA; 15.63281; 16.30946;
  
Sequence; C/EBPbeta [T00581]; 1644; 1647; 0.000000; TTGT; 15.63281; 16.30946;
  
Sequence; C/EBPbeta [T00581]; 1733; 1736; 1.366559; TCAA; 15.63281; 15.98168;
  
Sequence; C/EBPbeta [T00581]; 1747; 1750; 1.366559; TTGA; 15.63281; 15.98168;
  
Sequence; C/EBPbeta [T00581]; 1818; 1821; 1.366559; TCAA; 15.63281; 15.98168;
  
Sequence; C/EBPbeta [T00581]; 1822; 1825; 1.639871; CCAA; 15.63281; 15.98168;
  
Sequence; C/EBPbeta [T00581]; 1832; 1835; 0.000000; TTGC; 15.63281; 16.30946;
  
Sequence; C/EBPbeta [T00581]; 1843; 1846; 0.000000; GCAA; 15.63281; 16.30946;
  
Sequence; C/EBPbeta [T00581]; 1850; 1853; 1.366559; TTGA; 15.63281; 15.98168;
  
Sequence; C/EBPbeta [T00581]; 1923; 1926; 1.366559; TTGA; 15.63281; 15.98168;
  
Sequence; C/EBPbeta [T00581]; 1927; 1930; 0.000000; TTGT; 15.63281; 16.30946;
  
Sequence; C/EBPbeta [T00581]; 1966; 1969; 0.000000; ACAA; 15.63281; 16.30946;
  
Sequence; C/EBPalpha [T00105]; 235; 241; 6.460799; CATTGAC; 0.48853; 0.48636;
  
Sequence; C/EBPalpha [T00105]; 453; 459; 5.455853; CATTGCT; 0.73279; 0.75544;
  
Sequence; C/EBPalpha [T00105]; 528; 534; 2.441016; CTCAATG; 0.48853; 0.48755;
  
Sequence; C/EBPalpha [T00105]; 562; 568; 5.240291; TATTGCC; 0.97705; 1.02484;
  
Sequence; C/EBPalpha [T00105]; 740; 746; 0.000000; CTCAATC; 0.24426; 0.24053;
  
Sequence; C/EBPalpha [T00105]; 1098; 1104; 6.391486; GCCAATT; 0.48853; 0.48636;
  
Sequence; C/EBPalpha [T00105]; 1602; 1608; 2.371703; CCCAATT; 0.48853; 0.48755;
  
Sequence; C/EBPalpha [T00105]; 1613; 1619; 0.000000; CTCAATC; 0.24426; 0.24053;
  
Sequence; C/EBPalpha [T00105]; 1621; 1627; 8.006685; CATTGTA; 0.24426; 0.24698;
  
Sequence; C/EBPalpha [T00105]; 1638; 1644; 5.850545; GGCAATT; 0.97705; 1.01326;
  
Sequence; C/EBPalpha [T00105]; 1745; 1751; 1.830762; AATTGAG; 0.48853; 0.50538;
  
Sequence; C/EBPalpha [T00105]; 1821; 1827; 3.555778; ACCAATC; 0.24426; 0.25883;
  
Sequence; C/EBPalpha [T00105]; 1848; 1854; 6.855490; AATTGAA; 0.73279; 0.75722;
  
Sequence; C/EBPalpha [T00105]; 1925; 1931; 0.540941; GATTGTG; 0.24426; 0.24556;
  
Sequence; C/EBPalpha [T00105]; 1965; 1971; 6.786177; TACAATA; 0.73279; 0.75722;
  
Sequence; NF-1 [T00539]; 255; 262; 8.790071; TTGGGAAG; 0.24426; 0.23974;
  
Sequence; NF-1 [T00539]; 370; 377; 6.948522; GGCCCCAA; 0.48853; 0.49381;
  
Sequence; NF-1 [T00539]; 572; 579; 2.067686; TTGGGACA; 0.12213; 0.11807;
  
Sequence; NF-1 [T00539]; 633; 640; 2.813149; AGGGCCAA; 0.24426; 0.23808;
  
Sequence; NF-1 [T00539]; 836; 843; 9.513281; CAACCCAA; 0.73279; 0.73819;
  
Sequence; NF-1 [T00539]; 936; 943; 8.790071; TTGGGAAG; 0.24426; 0.23974;
  
Sequence; NF-1 [T00539]; 1095; 1102; 8.191058; AAGGCCAA; 0.24426; 0.25363;
  
Sequence; NF-1 [T00539]; 1105; 1112; 12.326430; TAATCCAA; 0.73279; 0.77824;
  
Sequence; NF-1 [T00539]; 1171; 1178; 4.880836; TTGGCACT; 0.24426; 0.24167;
  
Sequence; NF-1 [T00539]; 1319; 1326; 10.857758; TCCCCCAA; 0.61066; 0.61529;
  
Sequence; NF-1 [T00539]; 1333; 1340; 9.513281; TTGGGGTG; 0.73279; 0.73819;
  
Sequence; NF-1 [T00539]; 1475; 1482; 11.603221; TTGGGAAC; 0.48853; 0.49017;
  
Sequence; NF-1 [T00539]; 1517; 1524; 10.857758; TTGGGGAA; 0.61066; 0.61529;
  
Sequence; NF-1 [T00539]; 1575; 1582; 13.670907; TTGGGTAT; 0.97705; 0.99747;
  
Sequence; NF-1 [T00539]; 1593; 1600; 10.857758; TTGGGGGG; 0.61066; 0.61529;
  
Sequence; NF-1 [T00539]; 1599; 1606; 6.948522; GGACCCAA; 0.48853; 0.49381;
  
Sequence; NF-1 [T00539]; 1818; 1825; 13.670907; TCAACCAA; 0.97705; 0.99747;
  
Sequence; ER-alpha [T00261]; 279; 283; 0.000000; TGACC; 1.95410; 1.89644;
  
Sequence; ER-alpha [T00261]; 294; 298; 0.000000; GGTCA; 1.95410; 1.89644;
  
Sequence; ER-alpha [T00261]; 402; 406; 0.000000; TGACC; 1.95410; 1.89644;
  
Sequence; ER-alpha [T00261]; 667; 671; 0.000000; GGTCA; 1.95410; 1.89644;
  
Sequence; ER-alpha [T00261]; 1250; 1254; 0.000000; GGTCA; 1.95410; 1.89644;
  
Sequence; ER-alpha [T00261]; 1419; 1423; 0.000000; TGACC; 1.95410; 1.89644;
  
Sequence; ER-alpha [T00261]; 1659; 1663; 0.000000; TGACC; 1.95410; 1.89644;
  
Sequence; RAR-alpha1 [T00719]; 273; 285; 12.559902; GTCCCCTGACCCT; 0.05430; 0.05169;
  
Sequence; RAR-alpha1 [T00719]; 1248; 1260; 4.596281; GGGGTCATGGTGA; 0.00376; 0.00348;
  
Sequence; RAR-alpha1 [T00719]; 1413; 1425; 12.372473; ATCCTGTGACCCC; 0.05430; 0.05169;
  
Sequence; c-Jun [T00133]; 238; 244; 5.590308; TGACACT; 0.48853; 0.47565;
  
Sequence; c-Jun [T00133]; 279; 285; 6.293948; TGACCCT; 0.61066; 0.59680;
  
Sequence; c-Jun [T00133]; 292; 298; 5.587335; TAGGTCA; 0.48853; 0.47565;
  
Sequence; c-Jun [T00133]; 402; 408; 8.832178; TGACCTT; 0.61066; 0.60698;
  
Sequence; c-Jun [T00133]; 618; 624; 7.052189; TGACTAT; 0.73279; 0.72470;
  
Sequence; c-Jun [T00133]; 665; 671; 5.587335; TAGGTCA; 0.48853; 0.47565;
  
Sequence; c-Jun [T00133]; 788; 794; 7.538568; GATGTCA; 0.48853; 0.48282;
  
Sequence; c-Jun [T00133]; 1004; 1010; 5.193102; TGACTTC; 0.61066; 0.60396;
  
Sequence; c-Jun [T00133]; 1248; 1254; 5.703976; GGGGTCA; 0.48853; 0.47565;
  
Sequence; c-Jun [T00133]; 1269; 1275; 7.937147; TGACTAG; 0.48853; 0.48962;
  
Sequence; c-Jun [T00133]; 1419; 1425; 5.703976; TGACCCC; 0.48853; 0.47565;
  
Sequence; c-Jun [T00133]; 1547; 1553; 7.096776; TGACTGC; 0.73279; 0.72470;
  
Sequence; c-Jun [T00133]; 1649; 1655; 9.013496; CACGTCA; 0.61066; 0.60698;
  
Sequence; c-Jun [T00133]; 1659; 1665; 5.587335; TGACCTA; 0.48853; 0.47565;
  
Sequence; COUP-TF1 [T00149]; 289; 301; 7.555558; CCATAGGTCACAC; 0.01193; 0.01180;
  
Sequence; PEA3 [T00685]; 307; 315; 4.308180; GGACATCCA; 0.13740; 0.13895;
  
Sequence; PEA3 [T00685]; 334; 342; 9.937959; TCTCATCCC; 0.18320; 0.17864;
  
Sequence; PEA3 [T00685]; 600; 608; 4.308180; AGGATGAGT; 0.13740; 0.13895;
  
Sequence; PEA3 [T00685]; 1409; 1417; 1.194633; ACACATCCT; 0.06870; 0.06793;
  
Sequence; PEA3 [T00685]; 1581; 1589; 9.937959; ATTCATCCC; 0.18320; 0.17864;
  
Sequence; PEA3 [T00685]; 1780; 1788; 7.421728; GCCCATCCT; 0.34349; 0.33328;
  
Sequence; EBF [T05427]; 243; 253; 14.532033; CTCCCTGAAGA; 0.06107; 0.05931;
  
Sequence; EBF [T05427]; 312; 322; 10.413145; TCCACAGGGTG; 0.07061; 0.06691;
  
Sequence; EBF [T05427]; 565; 575; 14.096609; TGCCCTGTTGG; 0.06297; 0.05767;
  
Sequence; EBF [T05427]; 591; 601; 14.947733; GACACAGGGAG; 0.03244; 0.03060;
  
Sequence; EBF [T05427]; 714; 724; 6.595238; GCCCCTGTGGA; 0.01908; 0.01753;
  
Sequence; EBF [T05427]; 1954; 1964; 11.702104; CTCCCTGAGCT; 0.03817; 0.03581;
  
Sequence; MAZ [T00490]; 48; 60; 14.134899; GAGGGGGAAGGAG; 0.03006; 0.02742;
  
Sequence; MAZ [T00490]; 338; 350; 4.524062; ATCCCTCCCTCCC; 0.00188; 0.00168;
  
Sequence; MAZ [T00490]; 342; 354; 7.184780; CTCCCTCCCACTC; 0.00918; 0.00848;
  
Sequence; MAZ [T00490]; 723; 735; 14.121284; GAAGGGGAAGGTG; 0.03006; 0.02742;
  
Sequence; MAZ [T00490]; 1496; 1508; 14.932273; AGCCCACCCCCTC; 0.06387; 0.05874;
  
Sequence; Egr-3 [T00243]; 316; 328; 12.102248; CAGGGTGGGCGTG; 0.03840; 0.03683;
  
Sequence; Egr-3 [T00243]; 344; 356; 13.640068; CCCTCCCACTCAC; 0.04186; 0.04075;
  
Sequence; Ik-1 [T02702]; 248; 260; 14.245790; TGAAGAGTTGGGA; 0.12004; 0.11440;
  
Sequence; Ik-1 [T02702]; 347; 359; 11.871492; TCCCACTCACTTC; 0.06449; 0.06096;
  
Sequence; AhR [T01795]; 357; 367; 11.463981; TTCACGCTGGC; 0.03053; 0.02859;
  
Sequence; AhR:Arnt [T05394]; 320; 329; 8.431005; GTGGGCGTGG; 0.07442; 0.06841;
  
Sequence; AhR:Arnt [T05394]; 358; 367; 11.241339; TCACGCTGGC; 0.15457; 0.14516;
  
Sequence; NFI/CTF [T00094]; 251; 258; 14.601592; AGAGTTGG; 1.46558; 1.53919;
  
Sequence; NFI/CTF [T00094]; 374; 381; 10.579747; CCAAGGTG; 0.67172; 0.68330;
  
Sequence; NFI/CTF [T00094]; 568; 575; 10.270346; CCTGTTGG; 0.67172; 0.68330;
  
Sequence; NFI/CTF [T00094]; 637; 644; 10.579747; CCAAGGCA; 0.67172; 0.68330;
  
Sequence; NFI/CTF [T00094]; 840; 847; 10.270346; CCAACTGG; 0.67172; 0.68330;
  
Sequence; NFI/CTF [T00094]; 911; 918; 14.373419; CCAAGACA; 1.46558; 1.53919;
  
Sequence; NFI/CTF [T00094]; 932; 939; 10.579747; TTCTTTGG; 0.67172; 0.68330;
  
Sequence; NFI/CTF [T00094]; 1099; 1106; 14.373419; CCAATTTA; 1.46558; 1.53919;
  
Sequence; NFI/CTF [T00094]; 1109; 1116; 9.352332; CCAAGGTT; 0.54959; 0.54849;
  
Sequence; NFI/CTF [T00094]; 1159; 1166; 13.146004; CCAAATTC; 1.09918; 1.14054;
  
Sequence; NFI/CTF [T00094]; 1167; 1174; 10.270346; TCAGTTGG; 0.67172; 0.68330;
  
Sequence; NFI/CTF [T00094]; 1323; 1330; 5.558661; CCAAGCAC; 0.54959; 0.55284;
  
Sequence; NFI/CTF [T00094]; 1329; 1336; 9.042931; ACAGTTGG; 0.48853; 0.49774;
  
Sequence; NFI/CTF [T00094]; 1471; 1478; 6.786076; CTGCTTGG; 0.73279; 0.74706;
  
Sequence; NFI/CTF [T00094]; 1513; 1520; 14.373419; TTACTTGG; 1.46558; 1.53919;
  
Sequence; NFI/CTF [T00094]; 1571; 1578; 13.146004; AGTCTTGG; 1.09918; 1.14054;
  
Sequence; NFI/CTF [T00094]; 1589; 1596; 8.241664; CAGGTTGG; 0.18320; 0.18576;
  
Sequence; NFI/CTF [T00094]; 1603; 1610; 13.146004; CCAATTAT; 1.09918; 1.14054;
  
Sequence; NFI/CTF [T00094]; 1822; 1829; 5.558661; CCAATCAT; 0.54959; 0.55284;
  
Sequence; AR [T00040]; 307; 315; 6.725174; GGACATCCA; 0.19846; 0.19530;
  
Sequence; AR [T00040]; 444; 452; 5.896500; GAGTTGTCC; 0.24426; 0.24509;
  
Sequence; AR [T00040]; 552; 560; 8.113320; GGACAGGTG; 0.19846; 0.19445;
  
Sequence; AR [T00040]; 575; 583; 7.406474; GGACAGATG; 0.25190; 0.24966;
  
Sequence; AR [T00040]; 1343; 1351; 7.406474; GGACAGCTG; 0.25190; 0.24966;
  
Sequence; AR [T00040]; 1641; 1649; 7.623968; AATTTGTCC; 0.25190; 0.24966;
  
Sequence; AR [T00040]; 1980; 1988; 5.104816; GGACAGAGA; 0.11450; 0.11128;
  
Sequence; PPAR-alpha:RXR-alpha [T05221]; 365; 375; 11.027510; GGCTGGGCCCC; 0.04007; 0.03722;
  
Sequence; PPAR-alpha:RXR-alpha [T05221]; 447; 457; 14.659739; TTGTCCCATTG; 0.15457; 0.15196;
  
Sequence; PPAR-alpha:RXR-alpha [T05221]; 505; 515; 9.158357; ATCTGGGTCCA; 0.05152; 0.04653;
  
Sequence; PPAR-alpha:RXR-alpha [T05221]; 1212; 1222; 14.659739; GTGCCTCAGTT; 0.15457; 0.15196;
  
Sequence; PPAR-alpha:RXR-alpha [T05221]; 1528; 1538; 8.543553; AAGGCCCAGAG; 0.02004; 0.01976;
  
Sequence; XBP-1 [T00902]; 307; 312; 11.373637; GGACAT; 1.95410; 1.92243;
  
Sequence; XBP-1 [T00902]; 334; 339; 8.756040; TCTCAT; 2.93115; 3.02458;
  
Sequence; XBP-1 [T00902]; 461; 466; 12.067266; ATGCAG; 2.93115; 2.91335;
  
Sequence; XBP-1 [T00902]; 496; 501; 6.478682; ATGCCC; 0.97705; 0.94938;
  
Sequence; XBP-1 [T00902]; 515; 520; 9.789909; ATGGCT; 1.95410; 1.93798;
  
Sequence; XBP-1 [T00902]; 532; 537; 13.650994; ATGCTC; 2.93115; 2.91102;
  
Sequence; XBP-1 [T00902]; 581; 586; 8.756040; ATGAAA; 2.93115; 3.02458;
  
Sequence; XBP-1 [T00902]; 603; 608; 7.172312; ATGAGT; 2.93115; 2.99376;
  
Sequence; XBP-1 [T00902]; 702; 707; 11.373637; ATGGCA; 1.95410; 1.92243;
  
Sequence; XBP-1 [T00902]; 789; 794; 11.373637; ATGTCA; 1.95410; 1.92243;
  
Sequence; XBP-1 [T00902]; 1115; 1120; 8.756040; TTTCAT; 2.93115; 3.02458;
  
Sequence; XBP-1 [T00902]; 1250; 1255; 1.583727; GGTCAT; 0.97705; 0.98860;
  
Sequence; XBP-1 [T00902]; 1273; 1278; 13.650994; TAGCAT; 2.93115; 2.91102;
  
Sequence; XBP-1 [T00902]; 1383; 1388; 8.756040; ATGAAA; 2.93115; 3.02458;
  
Sequence; XBP-1 [T00902]; 1505; 1510; 7.172312; CCTCAT; 2.93115; 2.99376;
  
Sequence; XBP-1 [T00902]; 1581; 1586; 7.172312; ATTCAT; 2.93115; 2.99376;
  
Sequence; XBP-1 [T00902]; 1677; 1682; 8.756040; ATGAGA; 2.93115; 3.02458;
  
Sequence; XBP-1 [T00902]; 1802; 1807; 12.067266; ATGCAG; 2.93115; 2.91335;
  
Sequence; XBP-1 [T00902]; 1810; 1815; 8.756040; GTTCAT; 2.93115; 3.02458;
  
Sequence; XBP-1 [T00902]; 1824; 1829; 7.172312; AATCAT; 2.93115; 2.99376;
  
Sequence; XBP-1 [T00902]; 1841; 1846; 13.650994; ATGCAA; 2.93115; 2.91102;
  
Sequence; XBP-1 [T00902]; 1940; 1945; 7.172312; ATGAAT; 2.93115; 2.99376;
  
Sequence; XBP-1 [T00902]; 1944; 1949; 7.172312; ATGAAG; 2.93115; 2.99376;
  
Sequence; HNF-3alpha [T02512]; 491; 498; 14.000258; TGGAAATG; 1.64877; 1.75654;
  
Sequence; HNF-3alpha [T02512]; 1021; 1028; 8.343064; TATTTCAA; 0.27480; 0.30228;
  
Sequence; HNF-3alpha [T02512]; 1101; 1108; 11.843128; AATTTAAT; 0.39693; 0.43236;
  
Sequence; HNF-3alpha [T02512]; 1158; 1165; 14.000258; ACCAAATT; 1.64877; 1.75654;
  
Sequence; HNF-3alpha [T02512]; 1231; 1238; 4.842999; ATAAAATA; 0.09160; 0.10366;
  
Sequence; HNF-3alpha [T02512]; 1491; 1498; 10.500194; TATTTAGC; 1.19078; 1.30454;
  
Sequence; HNF-3alpha [T02512]; 1508; 1515; 7.000129; CATTTTTA; 0.82439; 0.91740;
  
Sequence; HNF-3alpha [T02512]; 1608; 1615; 14.000258; TATTTCTC; 1.64877; 1.75654;
  
Sequence; HNF-3alpha [T02512]; 1757; 1764; 7.000129; AAAAAATT; 0.82439; 0.91740;
  
Sequence; HNF-3alpha [T02512]; 1788; 1795; 0.000000; TAAAAATA; 0.09160; 0.10507;
  
Sequence; HNF-3alpha [T02512]; 1827; 1834; 14.000258; CATTTTTG; 1.64877; 1.75654;
  
Sequence; HNF-3alpha [T02512]; 1844; 1851; 10.500194; CAAAAATT; 1.19078; 1.30454;
  
Sequence; HNF-3alpha [T02512]; 1900; 1907; 10.500194; AAAAAATC; 1.19078; 1.30454;
  
Sequence; GR-beta [T01920]; 218; 222; 4.201913; TGATT; 7.81641; 8.25732;
  
Sequence; GR-beta [T01920]; 234; 238; 0.000000; ACATT; 3.90820; 4.09514;
  
Sequence; GR-beta [T01920]; 452; 456; 0.840383; CCATT; 7.81641; 8.10172;
  
Sequence; GR-beta [T01920]; 495; 499; 1.680765; AATGC; 3.90820; 3.97572;
  
Sequence; GR-beta [T01920]; 531; 535; 1.680765; AATGC; 3.90820; 3.97572;
  
Sequence; GR-beta [T01920]; 561; 565; 4.201913; CTATT; 7.81641; 8.25732;
  
Sequence; GR-beta [T01920]; 608; 612; 4.201913; TGATT; 7.81641; 8.25732;
  
Sequence; GR-beta [T01920]; 701; 705; 0.840383; AATGG; 7.81641; 8.10172;
  
Sequence; GR-beta [T01920]; 743; 747; 4.201913; AATCA; 7.81641; 8.25732;
  
Sequence; GR-beta [T01920]; 908; 912; 5.042296; AATCC; 3.90820; 4.03939;
  
Sequence; GR-beta [T01920]; 999; 1003; 5.042296; AATCC; 3.90820; 4.03939;
  
Sequence; GR-beta [T01920]; 1020; 1024; 4.201913; TTATT; 7.81641; 8.25732;
  
Sequence; GR-beta [T01920]; 1100; 1104; 0.840383; CAATT; 7.81641; 8.10172;
  
Sequence; GR-beta [T01920]; 1101; 1105; 0.000000; AATTT; 3.90820; 4.09514;
  
Sequence; GR-beta [T01920]; 1106; 1110; 5.042296; AATCC; 3.90820; 4.03939;
  
Sequence; GR-beta [T01920]; 1117; 1121; 0.840383; TCATT; 7.81641; 8.10172;
  
Sequence; GR-beta [T01920]; 1161; 1165; 0.000000; AAATT; 3.90820; 4.09514;
  
Sequence; GR-beta [T01920]; 1162; 1166; 1.680765; AATTC; 3.90820; 3.97572;
  
Sequence; GR-beta [T01920]; 1203; 1207; 3.361531; AATAT; 3.90820; 4.09514;
  
Sequence; GR-beta [T01920]; 1235; 1239; 4.201913; AATAG; 7.81641; 8.25732;
  
Sequence; GR-beta [T01920]; 1376; 1380; 5.042296; AATCC; 3.90820; 4.03939;
  
Sequence; GR-beta [T01920]; 1382; 1386; 0.840383; AATGA; 7.81641; 8.10172;
  
Sequence; GR-beta [T01920]; 1387; 1391; 4.201913; AATCG; 7.81641; 8.25732;
  
Sequence; GR-beta [T01920]; 1461; 1465; 0.840383; AATGG; 7.81641; 8.10172;
  
Sequence; GR-beta [T01920]; 1490; 1494; 3.361531; ATATT; 3.90820; 4.09514;
  
Sequence; GR-beta [T01920]; 1507; 1511; 0.840383; TCATT; 7.81641; 8.10172;
  
Sequence; GR-beta [T01920]; 1579; 1583; 5.042296; GTATT; 3.90820; 4.03939;
  
Sequence; GR-beta [T01920]; 1604; 1608; 0.840383; CAATT; 7.81641; 8.10172;
  
Sequence; GR-beta [T01920]; 1605; 1609; 0.840383; AATTA; 7.81641; 8.10172;
  
Sequence; GR-beta [T01920]; 1607; 1611; 4.201913; TTATT; 7.81641; 8.25732;
  
Sequence; GR-beta [T01920]; 1616; 1620; 5.042296; AATCC; 3.90820; 4.03939;
  
Sequence; GR-beta [T01920]; 1620; 1624; 0.840383; CCATT; 7.81641; 8.10172;
  
Sequence; GR-beta [T01920]; 1625; 1629; 5.042296; GTATT; 3.90820; 4.03939;
  
Sequence; GR-beta [T01920]; 1634; 1638; 0.840383; AATGG; 7.81641; 8.10172;
  
Sequence; GR-beta [T01920]; 1640; 1644; 0.840383; CAATT; 7.81641; 8.10172;
  
Sequence; GR-beta [T01920]; 1641; 1645; 0.000000; AATTT; 3.90820; 4.09514;
  
Sequence; GR-beta [T01920]; 1676; 1680; 0.840383; AATGA; 7.81641; 8.10172;
  
Sequence; GR-beta [T01920]; 1731; 1735; 4.201913; AATCA; 7.81641; 8.25732;
  
Sequence; GR-beta [T01920]; 1744; 1748; 0.840383; TAATT; 7.81641; 8.10172;
  
Sequence; GR-beta [T01920]; 1745; 1749; 0.840383; AATTG; 7.81641; 8.10172;
  
Sequence; GR-beta [T01920]; 1760; 1764; 0.000000; AAATT; 3.90820; 4.09514;
  
Sequence; GR-beta [T01920]; 1761; 1765; 0.840383; AATTA; 7.81641; 8.10172;
  
Sequence; GR-beta [T01920]; 1763; 1767; 4.201913; TTATT; 7.81641; 8.25732;
  
Sequence; GR-beta [T01920]; 1792; 1796; 4.201913; AATAG; 7.81641; 8.25732;
  
Sequence; GR-beta [T01920]; 1801; 1805; 1.680765; AATGC; 3.90820; 3.97572;
  
Sequence; GR-beta [T01920]; 1824; 1828; 4.201913; AATCA; 7.81641; 8.25732;
  
Sequence; GR-beta [T01920]; 1826; 1830; 0.840383; TCATT; 7.81641; 8.10172;
  
Sequence; GR-beta [T01920]; 1847; 1851; 0.000000; AAATT; 3.90820; 4.09514;
  
Sequence; GR-beta [T01920]; 1848; 1852; 0.840383; AATTG; 7.81641; 8.10172;
  
Sequence; GR-beta [T01920]; 1863; 1867; 4.201913; TTATT; 7.81641; 8.25732;
  
Sequence; GR-beta [T01920]; 1904; 1908; 5.042296; AATCC; 3.90820; 4.03939;
  
Sequence; GR-beta [T01920]; 1924; 1928; 4.201913; TGATT; 7.81641; 8.25732;
  
Sequence; GR-beta [T01920]; 1939; 1943; 0.840383; AATGA; 7.81641; 8.10172;
  
Sequence; GR-beta [T01920]; 1943; 1947; 0.840383; AATGA; 7.81641; 8.10172;
  
Sequence; GR-beta [T01920]; 1968; 1972; 3.361531; AATAT; 3.90820; 4.09514;
  
Sequence; TFIID [T00820]; 206; 212; 9.552105; TTTCTCA; 1.46558; 1.52520;
  
Sequence; TFIID [T00820]; 582; 588; 1.537547; TGAAAAA; 0.73279; 0.80733;
  
Sequence; TFIID [T00820]; 935; 941; 9.552105; TTTGGGA; 1.46558; 1.52520;
  
Sequence; TFIID [T00820]; 1045; 1051; 4.007279; TTTTAAA; 1.09918; 1.18556;
  
Sequence; TFIID [T00820]; 1230; 1236; 0.000000; TATAAAA; 1.09918; 1.23954;
  
Sequence; TFIID [T00820]; 1610; 1616; 9.552105; TTTCTCA; 1.46558; 1.52520;
  
Sequence; TFIID [T00820]; 1739; 1745; 0.000000; TTTTTTA; 1.09918; 1.23954;
  
Sequence; TFIID [T00820]; 1740; 1746; 0.000000; TTTTTAA; 1.09918; 1.23954;
  
Sequence; TFIID [T00820]; 1756; 1762; 0.000000; TAAAAAA; 1.09918; 1.23954;
  
Sequence; TFIID [T00820]; 1785; 1791; 5.544826; TCCTAAA; 0.73279; 0.77217;
  
Sequence; TFIID [T00820]; 1842; 1848; 3.075094; TGCAAAA; 0.12213; 0.13137;
  
Sequence; TFIID [T00820]; 1850; 1856; 0.000000; TTGAAAA; 1.09918; 1.23954;
  
Sequence; TFIID [T00820]; 1862; 1868; 4.007279; TTTATTA; 1.09918; 1.18556;
  
Sequence; TFIID [T00820]; 1934; 1940; 0.000000; TTTTTAA; 1.09918; 1.23954;
  
Sequence; TFIID [T00820]; 1970; 1976; 0.000000; TATAAAA; 1.09918; 1.23954;
  
Sequence; ETF [T00270]; 647; 657; 14.123853; GCCCAGGCCTC; 0.07681; 0.06996;
  
Sequence; SRY [T00997]; 706; 714; 14.351228; CAGGCAAAG; 0.12213; 0.12426;
  
Sequence; SRY [T00997]; 742; 750; 5.086565; CAATCAAAG; 0.06107; 0.06589;
  
Sequence; SRY [T00997]; 934; 942; 13.352057; CTTTGGGAA; 0.24426; 0.24847;
  
Sequence; SRY [T00997]; 1482; 1490; 11.263007; CTTTGAGGA; 0.12213; 0.12424;
  
Sequence; TCF-4E [T02878]; 708; 714; 6.302385; GGCAAAG; 0.61066; 0.64855;
  
Sequence; TCF-4E [T02878]; 744; 750; 3.151193; ATCAAAG; 0.24426; 0.25496;
  
Sequence; TCF-4E [T02878]; 934; 940; 12.604771; CTTTGGG; 0.48853; 0.51426;
  
Sequence; TCF-4E [T02878]; 1482; 1488; 9.453578; CTTTGAG; 0.48853; 0.50415;
  
Sequence; GR [T05076]; 379; 385; 8.971049; GTGTTTG; 0.61066; 0.66082;
  
Sequence; GR [T05076]; 608; 614; 11.290546; TGATTTG; 1.46558; 1.52901;
  
Sequence; GR [T05076]; 710; 716; 8.971049; CAAAGCC; 0.61066; 0.66082;
  
Sequence; GR [T05076]; 746; 752; 11.290546; CAAAGGA; 1.46558; 1.52901;
  
Sequence; GR [T05076]; 906; 912; 12.734565; CAAATCC; 0.48853; 0.50140;
  
Sequence; GR [T05076]; 932; 938; 3.763516; TTCTTTG; 0.73279; 0.81837;
  
Sequence; GR [T05076]; 1480; 1486; 11.290546; AACTTTG; 1.46558; 1.52901;
  
Sequence; GR [T05076]; 1828; 1834; 0.000000; ATTTTTG; 0.36639; 0.44231;
  
Sequence; GR [T05076]; 1844; 1850; 0.000000; CAAAAAT; 0.36639; 0.44231;
  
Sequence; RAR-beta:RXR-alpha [T05420]; 745; 756; 14.955991; TCAAAGGATCCC; 0.20967; 0.19578;
  
Sequence; RAR-beta:RXR-alpha [T05420]; 1341; 1352; 9.970660; GGGGACAGCTGA; 0.06977; 0.06456;
  
Sequence; RAR-beta:RXR-alpha [T05420]; 1414; 1425; 8.434402; TCCTGTGACCCC; 0.00954; 0.00907;
  
Sequence; NF-AT2 [T01945]; 759; 768; 4.460114; GAGACTTTCC; 0.04198; 0.04348;
  
Sequence; NF-AT2 [T01945]; 965; 974; 11.415543; GGAAAGTAAG; 0.07252; 0.07704;
  
Sequence; NF-AT2 [T01945]; 1007; 1016; 13.964020; CTTCTTTTCC; 0.09732; 0.09988;
  
Sequence; NF-AT2 [T01945]; 1359; 1368; 9.787971; GGAAACATGT; 0.08778; 0.09545;
  
Sequence; NF-AT2 [T01945]; 1452; 1461; 12.186641; GGAAAGTAAA; 0.11450; 0.12055;
  
Sequence; NF-AT2 [T01945]; 1521; 1530; 12.186641; GGAAACTAAG; 0.11450; 0.12055;
  
Sequence; NF-AT2 [T01945]; 1728; 1737; 13.192922; GGAAATCAAC; 0.10686; 0.11134;
  
Sequence; NF-AT2 [T01945]; 1916; 1925; 7.292655; GGAAACCTTG; 0.01717; 0.01830;
  
Sequence; NF-AT1 [T01948]; 491; 500; 4.134416; TGGAAATGCC; 0.08397; 0.08593;
  
Sequence; NF-AT1 [T01948]; 760; 769; 4.134416; AGACTTTCCA; 0.08397; 0.08593;
  
Sequence; NF-AT1 [T01948]; 1358; 1367; 3.445347; TGGAAACATG; 0.07633; 0.07813;
  
Sequence; NF-AT1 [T01948]; 1451; 1460; 5.512555; TGGAAAGTAA; 0.05916; 0.06059;
  
Sequence; STAT1beta [T01573]; 72; 81; 14.492168; AAGGGGAAAC; 0.20610; 0.21418;
  
Sequence; STAT1beta [T01573]; 488; 497; 11.593735; CACTGGAAAT; 0.30914; 0.31770;
  
Sequence; STAT1beta [T01573]; 763; 772; 11.593735; CTTTCCAGAT; 0.30914; 0.31770;
  
Sequence; STAT1beta [T01573]; 961; 970; 11.593735; AAGAGGAAAG; 0.30914; 0.31770;
  
Sequence; STAT1beta [T01573]; 1011; 1020; 9.807397; TTTTCCTTGT; 0.14885; 0.15536;
  
Sequence; STAT1beta [T01573]; 1355; 1364; 11.593735; AGGTGGAAAC; 0.30914; 0.31770;
  
Sequence; STAT1beta [T01573]; 1448; 1457; 14.492168; CTCTGGAAAG; 0.20610; 0.21418;
  
Sequence; STAT1beta [T01573]; 1517; 1526; 11.593735; TTGGGGAAAC; 0.30914; 0.31770;
  
Sequence; STAT1beta [T01573]; 1724; 1733; 5.796867; CCCAGGAAAT; 0.15457; 0.15666;
  
Sequence; STAT1beta [T01573]; 1912; 1921; 11.593735; GCCGGGAAAC; 0.30914; 0.31770;
  
Sequence; GATA-1 [T00306]; 768; 773; 0.000000; CAGATA; 0.97705; 1.00668;
  
Sequence; GATA-1 [T00306]; 772; 777; 0.000000; TATCTG; 0.97705; 1.00668;
  
Sequence; GATA-1 [T00306]; 952; 957; 2.001358; GGGATA; 3.90820; 3.92134;
  
Sequence; GATA-1 [T00306]; 983; 988; 2.176375; TATCCA; 3.90820; 3.92134;
  
Sequence; GATA-1 [T00306]; 1205; 1210; 0.105011; TATCTC; 0.97705; 1.00668;
  
Sequence; GATA-1 [T00306]; 1487; 1492; 2.176375; AGGATA; 3.90820; 3.92134;
  
Sequence; NF-kappaB [T00590]; 801; 812; 9.869072; CGGTCCTTCCCC; 0.02314; 0.02235;
  
Sequence; NF-kappaB1 [T00593]; 74; 84; 0.987329; GGGGAAACCCA; 0.00239; 0.00226;
  
Sequence; NF-kappaB1 [T00593]; 802; 812; 7.313747; GGTCCTTCCCC; 0.04437; 0.04132;
  
Sequence; RelA [T00594]; 73; 83; 5.044619; AGGGGAAACCC; 0.01002; 0.00969;
  
Sequence; RelA [T00594]; 803; 813; 13.772886; GTCCTTCCCCA; 0.04580; 0.04503;
  
Sequence; RelA [T00594]; 1518; 1528; 10.834373; TGGGGAAACTA; 0.03721; 0.03690;
  
Sequence; E2F-1 [T01542]; 829; 836; 11.323028; CTCGCCGC; 0.48853; 0.45044;
  
Sequence; E2F-1 [T01542]; 1420; 1427; 10.518902; GACCCCGC; 0.61066; 0.56316;
  
Sequence; LEF-1 [T02905]; 707; 714; 9.724040; AGGCAAAG; 0.21373; 0.22140;
  
Sequence; LEF-1 [T02905]; 743; 750; 1.362541; AATCAAAG; 0.09160; 0.09929;
  
Sequence; LEF-1 [T02905]; 934; 941; 8.973041; CTTTGGGA; 0.54959; 0.57731;
  
Sequence; LEF-1 [T02905]; 1482; 1489; 8.457856; CTTTGAGG; 0.15266; 0.15387;
  
Sequence; c-Ets-2 [T00113]; 108; 116; 7.841160; TTCCTGGCA; 0.32059; 0.33409;
  
Sequence; c-Ets-2 [T00113]; 214; 222; 7.841160; TTCCTGATT; 0.32059; 0.33409;
  
Sequence; c-Ets-2 [T00113]; 675; 683; 1.644150; TTCCTCCCA; 0.04580; 0.04617;
  
Sequence; c-Ets-2 [T00113]; 960; 968; 0.000000; TAAGAGGAA; 0.00763; 0.00829;
  
Sequence; c-Ets-2 [T00113]; 1013; 1021; 4.091811; TTCCTTGTT; 0.16030; 0.17753;
  
Sequence; c-Ets-2 [T00113]; 1149; 1157; 3.288300; GGTGAGGAA; 0.18320; 0.18551;
  
Sequence; c-Ets-2 [T00113]; 1433; 1441; 8.339336; CTGTAGGAA; 0.13740; 0.13615;
  
Sequence; c-Ets-2 [T00113]; 1661; 1669; 8.912323; ACCTAGGAA; 0.27480; 0.27553;
  
Sequence; c-Ets-2 [T00113]; 1723; 1731; 8.912323; CCCCAGGAA; 0.27480; 0.27553;
  
Sequence; STAT5A [T04683]; 1081; 1093; 12.222268; CTAAAAGAAACTC; 0.02880; 0.03154;
  
Sequence; POU2F2 (Oct-2.1) [T00646]; 1101; 1111; 8.848549; AATTTAATCCA; 0.01145; 0.01228;
  
Sequence; POU2F2 (Oct-2.1) [T00646]; 1796; 1806; 8.346865; GCTGTAATGCA; 0.05152; 0.05330;
  
Sequence; TBP [T00794]; 1226; 1235; 3.743085; TCACTATAAA; 0.03053; 0.03292;
  
Sequence; TBP [T00794]; 1966; 1975; 1.871542; ACAATATAAA; 0.18320; 0.20114;
  
Sequence; MEF-2A [T01005]; 1228; 1238; 11.664036; ACTATAAAATA; 0.02338; 0.02954;
  
Sequence; MEF-2A [T01005]; 1785; 1795; 6.559479; TCCTAAAAATA; 0.02385; 0.02822;
  
Sequence; HNF-4alpha [T03828]; 1474; 1486; 12.669538; CTTGGGAACTTTG; 0.02755; 0.02847;
  
Sequence; TCF-4 [T02918]; 742; 751; 0.226120; CAATCAAAGG; 0.01145; 0.01168;
  
Sequence; TCF-4 [T02918]; 1481; 1490; 6.262917; ACTTTGAGGA; 0.04962; 0.05198;
  
Sequence; CREB [T00163]; 1647; 1655; 4.299974; TCCACGTCA; 0.04580; 0.04458;
  
Sequence; ATF-2 [T00167]; 1647; 1656; 3.454345; TCCACGTCAC; 0.01908; 0.01847;
  
Sequence; ATF [T00051]; 1648; 1659; 8.419848; CCACGTCACTGT; 0.07729; 0.07537;
  
Sequence; ATF3 [T01313]; 291; 298; 11.686201; ATAGGTCA; 0.82439; 0.81440;
  
Sequence; ATF3 [T01313]; 664; 671; 11.686201; CTAGGTCA; 0.82439; 0.81440;
  
Sequence; ATF3 [T01313]; 787; 794; 6.744803; TGATGTCA; 0.27480; 0.27813;
  
Sequence; ATF3 [T01313]; 1648; 1655; 11.686201; CCACGTCA; 0.82439; 0.81440;
  
Sequence; ATF3 [T01313]; 1659; 1666; 11.686201; TGACCTAG; 0.82439; 0.81440;
  
Sequence; GCF [T00320]; 1698; 1706; 7.186486; CCTCTGCGC; 0.45799; 0.42796;
  
Sequence; HOXD9 [T01424]; 556; 565; 13.169294; AGGTGCTATT; 0.19846; 0.21724;
  
Sequence; HOXD9 [T01424]; 1015; 1024; 8.224939; CCTTGTTATT; 0.08778; 0.09923;
  
Sequence; HOXD9 [T01424]; 1620; 1629; 11.174228; CCATTGTATT; 0.18701; 0.20989;
  
Sequence; HOXD9 [T01424]; 1968; 1977; 8.224939; AATATAAAAG; 0.08778; 0.09923;
  
Sequence; HOXD10 [T01425]; 556; 565; 13.169294; AGGTGCTATT; 0.19846; 0.21724;
  
Sequence; HOXD10 [T01425]; 1015; 1024; 8.224939; CCTTGTTATT; 0.08778; 0.09923;
  
Sequence; HOXD10 [T01425]; 1620; 1629; 11.174228; CCATTGTATT; 0.18701; 0.20989;
  
Sequence; HOXD10 [T01425]; 1968; 1977; 8.224939; AATATAAAAG; 0.08778; 0.09923;
  
Sequence; T3R-beta1 [T00851]; 466; 474; 5.591999; GAAAGGTGA; 0.21373; 0.20793;
  
Sequence; T3R-beta1 [T00851]; 728; 736; 4.481316; GGAAGGTGA; 0.27480; 0.27225;
  
Sequence; T3R-beta1 [T00851]; 1145; 1153; 10.034728; GTAGGGTGA; 0.13740; 0.13189;
  
Sequence; T3R-beta1 [T00851]; 1191; 1199; 3.351341; TACAGGTGA; 0.27480; 0.27267;
  
Sequence; T3R-beta1 [T00851]; 1252; 1260; 5.572705; TCATGGTGA; 0.21373; 0.20793;
  
Sequence; T3R-beta1 [T00851]; 1541; 1549; 3.332047; CTAAGGTGA; 0.27480; 0.27267;
  
Sequence; T3R-beta1 [T00851]; 1985; 1993; 3.370634; GAGAGGTGA; 0.27480; 0.27267;
  
Sequence; HNF-1C [T01951]; 1558; 1566; 11.598163; GTTAGCAAG; 0.18320; 0.19329;
  
Sequence; HNF-1C [T01951]; 1874; 1882; 8.067744; GTTAGAGAA; 0.19846; 0.20589;
  
Sequence; HNF-1B [T01950]; 1557; 1565; 11.681862; AGTTAGCAA; 0.08397; 0.08851;
  
Sequence; HNF-1B [T01950]; 1873; 1881; 8.482557; GGTTAGAGA; 0.11450; 0.11959;
  
Sequence; POU2F1 [T00641]; 1838; 1848; 13.231217; ACGATGCAAAA; 0.22900; 0.24344;
  
Sequence; NF-Y [T00150]; 1096; 1103; 1.749852; AGGCCAAT; 0.18320; 0.18605;
  
Sequence; NF-Y [T00150]; 1600; 1607; 3.732121; GACCCAAT; 0.48853; 0.48664;
  
Sequence; NF-Y [T00150]; 1819; 1826; 1.285795; CAACCAAT; 0.18320; 0.18605;
  
Sequence; VDR [T00885]; 1810; 1818; 8.079962; GTTCATGTT; 0.24426; 0.25117;
  
Sequence; VDR [T00885]; 1816; 1824; 3.462841; GTTCAACCA; 0.21373; 0.21122;
  
Sequence; PXR-1:RXR-alpha [T05671]; 1807; 1814; 2.454225; GAAGTTCA; 0.12213; 0.12133;
  
Sequence; PXR-1:RXR-alpha [T05671]; 1813; 1820; 7.362674; CATGTTCA; 0.24426; 0.24164;
  
Sequence; ATF-1 [T00968]; 1648; 1658; 10.493812; CCACGTCACTG; 0.15028; 0.14769;
  
Sequence; AP-1 [T00029]; 618; 626; 14.228908; TGACTATAG; 0.33586; 0.34421;
  
Sequence; AP-1 [T00029]; 1004; 1012; 13.416718; TGACTTCTT; 0.24426; 0.24321;
  
Sequence; AP-1 [T00029]; 1547; 1555; 13.810148; TGACTGCCT; 0.33586; 0.34421;
  
Sequence; RBP-Jkappa [T01616]; 1472; 1483; 11.751025; TGCTTGGGAACT; 0.02290; 0.02261;
  
Sequence; USF2 [T00878]; 555; 564; 8.532138; CAGGTGCTAT; 0.10305; 0.09880;
  
Sequence; USF2 [T00878]; 1193; 1202; 4.528187; CAGGTGATGT; 0.06870; 0.06543;
  
Sequence; CTF [T00174]; 1096; 1107; 14.459692; AGGCCAATTTAA; 0.06870; 0.06952;
  
Sequence; IRF-2 [T01491]; 210; 215; 0.000000; TCACTT; 0.48853; 0.50148;
  
Sequence; IRF-2 [T01491]; 353; 358; 0.000000; TCACTT; 0.48853; 0.50148;
  
  
-- END ---------------------------------------------------------------
  
